# Therapeutic Options for Alzheimer’s Disease and Aging-Associated Cognitive Decline: State of the Art in the ACH2.0 Paradigm

**DOI:** 10.3390/ijms27031486

**Published:** 2026-02-02

**Authors:** Vladimir Volloch, Sophia Rits-Volloch

**Affiliations:** 1 Department of Developmental Biology, Harvard School of Dental Medicine, Boston, MA 02115, USA; 2 Division of Molecular Medicine, Children’s Hospital, Boston, MA 02115, USA; 3 Department of Biological Chemistry and Molecular Pharmacology, Harvard Medical School, Boston, MA 02115, USA

**Keywords:** Alzheimer’s disease, Amyloid Cascade Hypothesis 2.0 (ACH2.0), intraneuronal Aβ (iAβ), C99, neuronal integrated stress response (ISR), AβPP-independent C99 production pathway, RNA-dependent amplification of AβPP mRNA, neuronal ISR inhibitors, targeted degradation of iAβ and C99, RNA-based therapies for AD

## Abstract

In the Amyloid Cascade Hypothesis (ACH2.0) paradigm, Alzheimer’s disease (AD) is defined as a disorder triggered by a sustained neuronal integrated stress response (ISR) and driven by the C99 fragment of amyloid-beta protein precursor (AβPP) generated in the autonomous AβPP-independent pathway. This implies that AD can be initiated by any stressor capable of activating one or more of the four eIF2α kinases and accumulated intraneuronally to sufficient levels. In most instances of AD, such a stressor is intraneuronal Aβ (iAβ) accumulated to a PKR- and/or HRI-activating concentration and designated, in terms of the ACH2.0, as a “conventional stressor”. The ensuing disease is referred to, accordingly, as “conventional AD”. Any stressor other than iAβ, which is capable of activating one or more eIF2α kinases in neuronal cells, is designated as an “unconventional stressor”. At a sufficient concentration, it triggers elicitation of the neuronal ISR and initiates the disease, referred to in terms of the ACH2.0 as “unconventional AD”, at levels of iAβ below those required for activation of PKR and/or HRI. In both forms of AD, the neuronal ISR activates production of components essential for, and, consequently, enables operation of, the RNA-dependent mRNA amplification pathway. Human AβPP mRNA is eligible for this process, and its asymmetric amplification yields 5′-truncated mRNA molecules that are translated into C99 at rates that are orders of magnitude greater than those seen in AβPP proteolysis. The resulting C99 drives AD pathology; it also propagates the ISR state and perpetuates both its own production and the progression of the disease. Thus, the neuronal ISR-enabled amplification of AβPP mRNA constitutes the active core of AD. It follows that the essence of any effective therapy for AD, in both conventional and unconventional forms, is to either prevent activation or suppress the operation of the AβPP mRNA amplification process. The present perspective considers therapeutic options capable of accomplishing these objectives. They include inhibition of the neuronal ISR, targeted degradation of iAβ and C99, anti-antisense oligonucleotides (AASO) for AβPP RNA, and the restructuring of the 5′ terminus of AβPP mRNA. Collectively, these therapeutic approaches constitute the state of the art in the ACH2.0 paradigm; if successful, they would render both AD and aging-associated cognitive decline (AACD) obsolete. This study also describes transgenic animal and human neuronal cell-based models of both conventional and unconventional forms of AD that are suitable for testing the proposed therapeutic strategies.

## 1. Introduction: Alzheimer’s Disease Is a Disorder of the Sustained Neuronal Integrated Stress Response (ISR)

Amyloid Cascade Hypothesis 2.0 (ACH2.0) was introduced, and its various aspects have been analyzed in our previous publications [1,2,3,4,5,6,7,8,9,10,11]. The principal tenet of the ACH2.0 is that Alzheimer’s disease is a disorder of the sustained neuronal integrated stress response [12]. The ISR in general, and neuronal ISR in particular, can be elicited in multiple ways, which are categorized and discussed below. Under the neuronal ISR conditions, principally as a result of phosphorylation of eIF2α at its Ser51 residue, the complexity of the cellular translational landscape as well as its pattern undergo dramatic metamorphosis [13,14,15,16,17,18,19,20,21,22]. As a result, the bulk of the cellular protein synthesis is suppressed. Among the proteins whose production is inhibited under ISR conditions are, apparently, the AβPP and enzymes that are required for its proteolytic processing, namely beta- and gamma-secretases [10,11]. Consequently, the production of AβPP-derived C99 and Aβ is also suppressed under the neuronal ISR conditions [10]. On the other hand, a cap-independent translation of a small subset of cellular proteins is activated. Among those are components that are essential for the operation of the AβPP-independent C99 production pathway. The presumed mechanism of AβPP-independent generation of C99 is described in Section 22 below. Briefly, the 3′-terminal portion of human AβPP mRNA is amplified via RNA-dependent mRNA synthesis. The resulting RNA molecule is AβPP mRNA, which is severely truncated at the 5′ end. The AUG normally encoding Met671 of AβPP becomes the first in-frame AUG codon within the 5′ truncated AβPP mRNA molecule generated in the amplification process. Moreover, this AUG is situated within the optimal translation initiation nucleotide context. Translation initiated from this AUG yields the C100 fragment of AβPP (i.e., N-terminal Met-C99). The N-terminal methionine of C100 is removed post-translationally by aminopeptidases with broad specificity; this transforms C100 into C99. Since gamma-secretase is among the cellular proteins whose production is suppressed under the neuronal ISR conditions, C99 generated independently of AβPP is not cleaved into Aβ but largely retains its integrity. The rate of its production is orders of magnitude greater than that of its counterpart produced by the proteolysis of AβPP. It rapidly accumulates, and it is, in fact, C99 generated independently of AβPP that drives AD pathology.

## 2. Conventional Alzheimer’s Disease: The Neuronal ISR Is Triggered by Intraneuronal Aβ Accumulated over the Critical Neuronal ISR-Eliciting Threshold

In the ACH theory of AD [23,24], the disease is caused and driven by secreted extracellular Aβ. At the time of its introduction in 1992 [24], this theory was consistent with the entirety of accumulated data. It was enthusiastically accepted and guided the development of the field in the decades following its formulation. This theory also guided the design of transgenic animals presumed to be models of AD, as well as the development of AD drugs. The underlying rationale for both was simple: produce exogenously sufficient levels of extracellular Aβ and the disease will ensue; deplete extracellular Aβ in transgenic animal models or in AD patients and the disease will be successfully treated. And, indeed, transgenic animals exogenously overexpressing human AβPP exhibited a degree of neurodegeneration and cognitive impairment that were interpreted as symptoms of AD. None of these models, however, were capable of developing a full spectrum of AD pathology: No formation of neurofibrillary tangles, the hallmark of AD, was seen in these models; as argued in Section 28 below, these transgenic animals model not AD but rather the deleterious effects of Aβ-triggered neuronal integrated stress response. On the other hand, Aβ-depleting drugs such as Aβ-specific antibodies and BACE1 inhibitors, which suppress production and subsequent secretion of Aβ in the AβPP proteolytic pathway, were highly successful in animal studies [25,26,27]. They not only reduced neurodegeneration but also restored the impaired cognitive function of animals overproducing Aβ from multiple human AβPP transgenes. 

All these drugs, however, failed completely in human clinical trials [28,29]. Importantly, the drugs’ failure was not due to their mechanistic underperformance. They performed mechanistically (in depleting extracellular Aβ) in humans as well, if not better, than in animal models. These results, therefore, indicated that the ACH theory could be incorrect. With the development of PET scan-based methods that enable evaluation of the deposition of extracellular Aβ, this notion was corroborated. It transpired that in a large fraction of the healthy aged population, levels of extracellular Aβ actually exceed those typically seen in AD patients and that, inversely, no excessive extracellular Aβ deposits can be seen in a fraction of AD patients [30,31,32,33,34,35,36,37]. These findings necessitated the formulation of an alternative theory of AD, dubbed ACH2.0 [1,2,3,4,5,6,7,8,9,10,11].

In the ACH2.0 paradigm, sustained elicitation of the neuronal integrated stress response results in AD. Elicitation of the neuronal ISR can be enacted by a variety of stressors. In most cases of the disease, elicitation of the neuronal ISR is triggered by intraneuronal Aβ (iAβ) accumulated over the critical threshold, a notion consistent with observations that the severity of AD symptoms correlates much better with levels of iAβ than with the extent of depositions of extracellular Aβ [38,39,40,41,42,43,44,45,46,47,48,49,50]. This category of the disease, where the elicitation of the neuronal ISR is triggered by iAβ, has been designated “conventional” AD [8,9,10,11]. Lifelong accumulation of iAβ is a physiological process and involves two distinct mechanisms. One is the intraneuronal retention of a fraction of Aβ derived by the proteolysis of AβPP. Aβ peptide is embedded within the AβPP, and its release requires two proteolytic cleavages, both occurring on cellular membranes. First cleavage, by beta-secretase (beta-site cleavage enzyme, BACE), occurs between amino acid residues 671 and 672 of AβPP and generates the C99 fragment (designated so because it comprises 99 amino acid C-terminal residues). C99 is the immediate precursor of Aβ, and its N-terminus is shared by both C99 and Aβ. The second proteolytic cleavage required for the production of Aβ occurs within C99 and releases Aβ of various lengths, typically 40 or 42 amino acid residues. This cleavage usually occurs on plasma membranes, and the released Aβ is secreted into the extracellular space. A fraction of cleavages, however, occur on intracellular membranes, and in these cases, the resulting Aβ is retained intraneuronally as iAβ [51,52,53,54,55,56,57,58,59].

Another mechanism contributing to the accumulation of intraneuronal Aβ is the cellular uptake of AβPP-derived Aβ secreted into the extracellular space [60,61,62,63,64,65]. Different isoforms of Aβ are internalized with different efficiencies. Thus, Aβ42 is taken up by the cell twice as efficiently as another major isoform, Aβ40. The reason for this became clear when it was elucidated that Aβ is taken up by the cell in the oligomeric form, and its oligomerization is a prerequisite for its internalization. Aβ42 has a substantially greater propensity for oligomerization than Aβ40, and hence, it is internalized with much greater efficiency [64,65]. Internalization of extracellular Aβ is enacted via various receptors and is regulated by ligands of these receptors [66,67,68,69,70,71,72,73,74]. A notable ligand involved in the internalization of extracellular Aβ is ApoE [64]. ApoE occurs in several isoforms, and one of them, namely ApoE4, is much more efficient in the cellular uptake of extracellular Aβ than other ApoE variants. Greater efficiency of Aβ internalization, in turn, accelerates the rate of accumulation of iAβ and thus increases the probability of the occurrence of AD. This is the reason why ApoE4 is considered a risk factor, if not a cause, of AD.

When levels of iAβ cross the critical threshold, designated T1, they trigger elicitation of the neuronal ISR, and AD ensues. This type of cellular stress response is dubbed “integrated” because, while it can be caused by a multitude of stressors, they all lead to the same core event (the integer), namely phosphorylation of eukaryotic translation initiation factor 2alpha (eIF2α) at its amino acid residue Ser51 [13,14,15,16,17,18,19,20,21,22]. This phosphorylation is enacted by the family of eIF2α kinases, which comprises four members: protein kinase double-stranded RNA-dependent (PKR), PKR-like endoplasmic reticulum kinase (PERK), general control non-derepressible-2 kinase (GCN2), and heme-regulated inhibitor kinase (HRI), with each kinase responding to a defined set of stressors. The eIF2α kinases exhibit significant homology in their catalytic domains but contain distinctly defined regulatory domains; this is why they respond in a similar manner (i.e., auto-phosphorylation and dimerization) and with identical results (i.e., phosphorylation of eIF2α) to distinct sets of stressors. It should be mentioned that under ISR conditions, suppression of cap-dependent translation is augmented by induction of 4E-BP (eIF4E binding protein), which is regulated, at least in part, by ATF4, whose production is activated downstream from, and as a result of, eIF2α phosphorylation. 4E-BP inhibits cap-dependent translation. It does this by binding to and thus inhibiting eIF4E, which normally binds to 5′-capG and mediates loading of the 43S ribosome complex. Apparently, 4E-BP fully exerts its translation inhibitory effect if and when phosphorylation of eIF2α is reversed (presumably by GADD34). The overall result, therefore, is the shift toward cap-independent translation under ISR conditions.

When accumulated over the critical T1 threshold, iAβ appears to be capable of activating two members of the eIF2α family of kinases, namely PKR and HRI. The enabling role of Aβ in the activation of PKR is supported by the following observations. Activation of PKR and phosphorylation of eIF2α correlate with levels of iAβ and occur in models overexpressing Aβ and thus accumulating iAβ [75,76,77]. Importantly, both activated PKR and phosphorylated eIF2α were observed in the neurons of AD patients [78,79]. Mechanistically, iAβ can, apparently, enact activation of PKR in two ways. One is via PACT (an abbreviation for PKR ACTivator). This mechanism is indicated by the finding that PACT and activated PKR are co-localized in the neuronal cells of Alzheimer’s patients [80]. An alternative or possible parallel pathway of the activation of PKR involves TNFα (tumor necrosis factor alpha), as seen in transgenic animal models overexpressing Aβ [81].

The iAβ-mediated activation of HRI, on the other hand, apparently follows a distinctly different pathway: it is a consequence of iAβ-caused mitochondrial dysfunction in neuronal cells, an early and comprehensively studied aspect of AD [82,83,84,85,86,87,88,89,90,91,92,93,94,95,96,97,98,99]. When mitochondrial dysfunction is triggered, OMA1, a mitochondrial protease, is activated. One of its substrates is a mitochondrial protein designated as DELE1. Activated OMA1 cleaves DELE1 at a defined position. One of the two resulting fragments of DELE1, exhibiting affinity to HRI, is translocated into the cytosol. When in the cytosol, it finds, binds to, and thus activates HRI. iAβ-mediated activation of either or both PKR and HRI leads to phosphorylation of eIF2α. This, in turn, results in the elicitation of the neuronal ISR [100,101]. Consequently, the AβPP-independent C99 production pathway is activated, and AD pathology commences.

**Figure 1:**
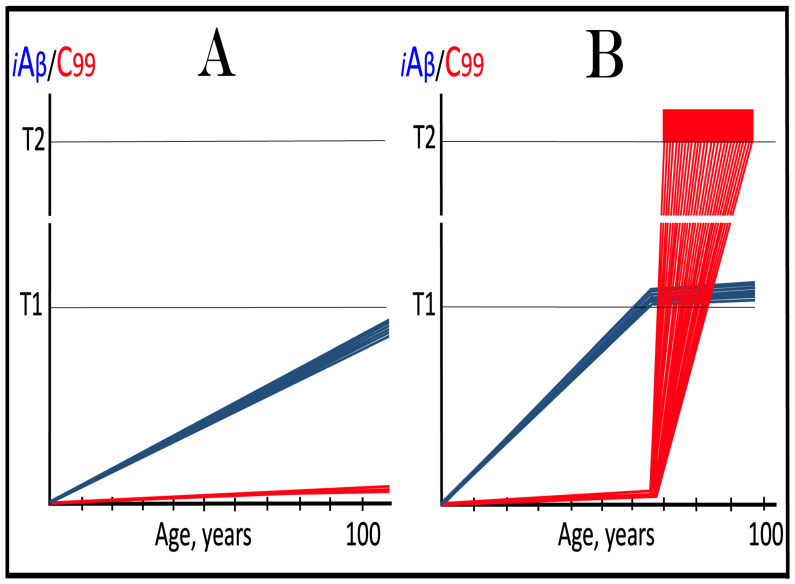
**Dynamics of iAβ and C99 accumulation in health and in conventional Alzheimer’s disease**. *Vertical axis*: Relative levels of iAβ and C99. *Horizontal axis*: Age, in years. *Blue lines*: Dynamics of iAβ. *Red lines*: Dynamics of C99. **T1**: Threshold of intracellular concentration of iAβ or C99 that mediates elicitation of the neuronal integrated stress response, which, in turn, enables operation of the AβPP-independent C99 generation pathway and thus triggers the disease. **T2**: Threshold of the intracellular concentration of C99 that triggers neuronal death. *Red box*: Range of intracellular concentrations of C99, dubbed “Apoptotic Zone”, associated with apoptosis or necroptosis of neuronal cells. Panel (**A**): iAβ accumulates, via cellular uptake of secreted extracellular Aβ and through retention of iAβ resulting from the processing of a fraction of AβPP-derived C99 on intraneuronal membranes, at an exceedingly slow rate. It does not reach the T1 threshold, and no conventional AD occurs within the lifetime of an individual. Even slower accumulation of AβPP-derived C99 reflects its nature as a short half-life intermediate that is cleaved by gamma-secretase to yield Aβ. Panel (**B**): iAβ crosses the T1 threshold. The PKR and/or HRI kinases are activated; eIF2α is phosphorylated at its residue Ser51, and the neuronal ISR is elicited. Under ISR conditions, production of AβPP, BACE enzymes, and gamma-secretase is inhibited, along with that of the bulk of cellular proteins. Consequently, accumulation of iAβ is suppressed. Concurrently, the ISR state enables the AβPP-independent generation of C99. It remains largely intact due to the deficit of gamma-secretase and, as a result, rapidly accumulates and drives AD pathology.

Dynamics of iAβ/C99 accumulation are presented in Figure 1. The process of physiological lifelong accumulation of AβPP-derived iAβ is very slow. In the majority of the general population, its levels do not reach the critical ISR-triggering threshold during a lifetime, and the disease does not occur (panel A of Figure 1). This explains why sporadic AD occurs late in life: Decades are required for iAβ to reach the T1 threshold. Following the crossing of the T1 threshold by iAβ, PKR and/or HRI are activated, eIF2α is phosphorylated, and the neuronal ISR is elicited. As a result, production and, consequently, accumulation of iAβ are suppressed. Simultaneously, the AβPP-independent C99 generation pathway is activated, and AD commences. C99 rapidly accumulates and drives AD pathology (panel B of Figure 1). It should be noted that in familial AD, inherited mutations cause substantial acceleration in the rate of iAβ accumulation and/or reduction in the extent of the critical T1 threshold. As a result, the T1 threshold is crossed much earlier in a lifetime, and the familial disease commences much sooner than its sporadic counterpart (further discussed and illustrated in Section 10 below).

## 3. Unconventional Alzheimer’s Disease: The Neuronal ISR Is Elicited by Stressors Different from Intraneuronal Aβ

As mentioned above and elaborated in our preceding studies, in the ACH2.0 paradigm, AD is a disease of the sustained neuronal integrated stress response. It follows that the disease can be triggered by the sustained neuronal integrated stress response elicited in any way whatsoever. Once the neuronal ISR is elicited, its provenance becomes irrelevant: regardless of its origin, it will supply components essential for the operation of the AβPP-independent C99 generation pathway, which drives the disease [1,2,3,4,5,6,7,8,9,10,11,12]. iAβ (referred to as “conventional stressor”) is only one, albeit prevalent, trigger of the neuronal integrated stress response of many. When accumulated over the critical T1 threshold, it triggers a category of the disease that has been designated “conventional AD”. In contrast, Alzheimer’s disease caused by the neuronal ISR elicited by a multitude of stressors distinct from AβPP-derived iAβ has been designated “unconventional AD” and neuronal ISR-eliciting stressors other than AβPP-derived iAβ have been referred to as “unconventional stressors” [8,9,10,11]. The rationale for grouping all cases of AD triggered by potentially widely distinct unconventional stressors in one category of “unconventional AD” is their common feature: they all commence at levels of AβPP-derived iAβ below the T1 threshold. Indeed, if an unconventional stressor were to appear after AβPP-derived iAβ has crossed the T1 threshold, its occurrence would be redundant, since the neuronal ISR has already been conventionally elicited and the disease has commenced.

For a stressor to qualify as “unconventional”, the only requirement (except being distinct from iAβ) is its capacity to activate one or more of the eIF2α kinases. Such stressors are copious, and so are the conditions that generate them. These conditions include traumatic brain injury (TBI) [102,103,104] and chronic traumatic encephalopathy (CTE) [105]. They also encompass both viral and bacterial infections [106,107,108,109,110,111,112,113,114]. This can be illustrated by findings showing that viral encephalitis increases the likelihood of the occurrence of AD thirtyfold [106,107,108,109,110], and many bacterial infections elevate it more than tenfold [111,112,113,114]. Moreover, interplay was shown to occur between mechanical and infectious sources of unconventional stressors. For example, HSV1, known for its association with AD [115,116,117,118], often establishes a dormant state in nerve tissues [116]. Recent experiments with human brain organoids harboring dormant HSV1 have shown that an equivalent of CTE (repeated mechanical brain injury) activates dormant virus and results in AD-associated phenotypes [119]. 

The unconventional occurrence of AD is also strongly associated with many types of inflammation. Not only does neuroinflammation drastically increases incidence of the disease; so do systemic inflammation and even localized inflammation, such as rheumatoid arthritis and osteoarthritis [120,121,122,123,124,125,126,127,128,129,130,131]. Instances of dementia associated with the conditions listed above are often referred to as ADRD (AD-related dementia) or ADLD (AD-like dementia). In the ACH2.0 paradigm, most, if not all, of these cases fall firmly into the category of unconventional AD.

How signals leading to the activation of eIF2α kinases and elicitation of the ISR in neuronal cells are transmitted under the mechanical, infectious, and inflammatory conditions described above remains to be established. On the other hand, all these conditions share two common features. One is a compromised blood–brain barrier [132]. This could allow unconventional stressors to penetrate the brain and, eventually, the neurons. Another common attribute is that all these conditions are associated with reduced cerebral blood flow (CBF) [133,134,135,136,137,138,139]. CBF can potentially elicit neuronal ISR in many ways. One of those ways is of special interest: the capacity of CBF to trigger mitochondrial dysfunction in neurons [140,141]. As described above, this would induce the OMA1 to DELE1 pathway and activate the HRI kinase [100,101], which, in turn, would phosphorylate eIF2α and elicit neuronal ISR.

**Figure 2:**
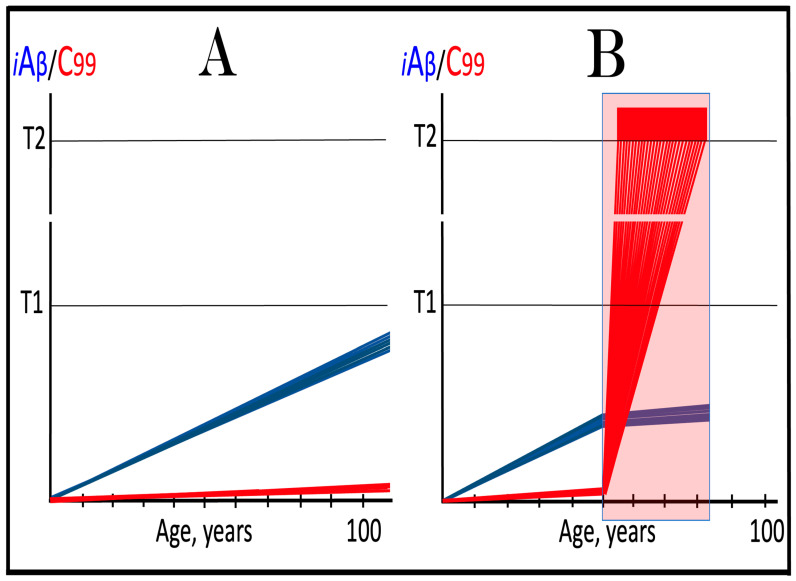
**Dynamics of iAβ and C99 accumulation in health and in unconventional AD**. *Vertical axis*: Relative levels of iAβ and C99. *Horizontal axis*: Age, in years. *Blue lines*: Dynamics of iAβ. *Red lines*: Dynamics of C99. **T1**: Threshold of intracellular concentration of iAβ or C99 that mediates elicitation of the neuronal integrated stress response, which, in turn, enables operation of the AβPP-independent C99 generation pathway and thus triggers the disease. **T2**: Threshold of intracellular concentration of C99 that triggers neuronal death. *Red box*: Range of intracellular concentrations of C99, dubbed “Apoptotic Zone”, associated with apoptosis or necroptosis of neuronal cells. *Pink box*: The duration of the occurrence of unconventional stressors (as defined in the main text) at concentrations sufficient to elicit the neuronal ISR. Panel (**A**): iAβ accumulates, via cellular uptake of secreted extracellular Aβ and through retention of iAβ resulting from the processing of a fraction of AβPP-derived C99 on intraneuronal membranes, at an exceedingly slow rate. It does not reach the T1 threshold, and no unconventional stressors occur sustainably within the lifetime of an individual; neither conventional nor unconventional AD develops in this scenario. Even slower accumulation of AβPP-derived C99 reflects its nature as a short half-life intermediate that is cleaved by gamma-secretase to yield Aβ. Panel (**B**): Unconventional stressors occur at under-T1 levels of iAβ and persist for the remaining portion of the lifespan. The neuronal ISR is unconventionally elicited. Under ISR conditions, production of AβPP, BACE enzymes, and gamma-secretase is inhibited along with that of the bulk of cellular proteins. Consequently, accumulation of iAβ is suppressed. Concurrently, the ISR state enables the AβPP-independent generation of C99. It remains largely intact due to the deficit of gamma-secretase and, as a result, rapidly accumulates. When it crosses the T1 threshold, the AβPP-independent C99 generation pathway is rendered self-sustainable and C99-driven unconventional AD commences.

To summarize the present section, once one or more eIF2α kinases have been sustainably activated by an unconventional stressor at low levels of iAβ, the AβPP-independent C99 production pathway is activated, and the dynamics of accumulation of AβPP-derived iAβ and of C99 generated independently of AβPP are principally the same as in conventional AD: the production and accumulation of the former is suppressed, and the rate of accumulation of the latter drastically increases, resulting in AD pathology. These dynamics are schematically illustrated in Figure 2.

## 4. C99 Generated Independently of AβPP Drives AD Pathology, Propagates the Neuronal ISR, and Perpetuates Its Own Production: The Engine That Enables and Drives the Disease

The two preceding sections describe the dynamics of AD in its conventional and unconventional forms. In both cases, the direct cause of the disease is the neuronal integrated stress response. In conventional AD, the elicitation of neuronal ISR is triggered by AβPP-derived iAβ accumulated over the critical T1 threshold. Two attributes differentiate unconventional AD from its conventional counterpart. One is that in conventional AD, the neuronal ISR is elicited by stressors other than AβPP-derived iAβ (i.e., by “unconventional stressors”). Another attribute is that, while in conventional AD iAβ triggers the elicitation of the neuronal ISR only upon its accumulation over the T1 threshold, unconventional stressors always cause the elicitation of the neuronal ISR at the levels of AβPP-derived iAβ below the T1 threshold; implications of this feature of unconventional AD are discussed below.

When the neuronal ISR is elicited, the production, and therefore the rate of accumulation of AβPP-derived iAβ, is suppressed, along with that of the bulk of cellular proteins. On the other hand, ISR conditions enable, via cap-independent translation, the production of components essential for operation of the AβPP-independent C99 generation pathway. In this pathway, C99 is produced at a rate that is orders of magnitude greater than in the AβPP proteolytic pathway (see below). Since production of gamma-secretase is suppressed under ISR conditions, C99 is not processed into Aβ but remains largely intact. It rapidly accumulates, and it is C99 that is generated independently of AβPP that drives AD pathology, which culminates in the formation of neurofibrillary tangles (in part due to iAβ/C99-mediated inhibition of the proteasome system [142,143,144,145]) and neuronal loss via apoptosis or necroptosis [146]. Indeed, C99, at sufficient levels, is known to interfere with lipid metabolism and to lead to an imbalance of lipid homeostasis. These effects are manifestations of the interaction of C99 with mitochondria-associated ER membranes (MAM) [147]. When accumulated to sufficient levels, C99 also triggers endosomal/autophagic/lysosomal distress (EAL dysfunction), a neuropathological feature of AD [148]. EAL dysfunction is caused by the aggregation of C99 within EAL vesicle membranes. This impedes lysosomal proteolysis and causes autophagic impairment. These conditions, if sustained (and they are sustained as discussed below), have the capacity to initiate and maintain cascades of cellular AD pathology. That this is indeed the case is, in fact, strongly indicated by the observations that (a) accumulation of C99 was indeed detected in AD patients, (b) accumulation of C99 in AD is selective—it accumulates only in disease-affected neurons but not in the healthy regions of the brain—and (c) in AD patients the extent of C99 accumulation in the disease-affected neurons correlates very well with the severity of cognitive impairment [149,150]. The activities of C99 in AD are not limited to its role in AD pathology. Importantly, it also sustains the neuronal ISR state, which is essential for operation of the AβPP-independent pathway of its own production.

**Figure 3:**
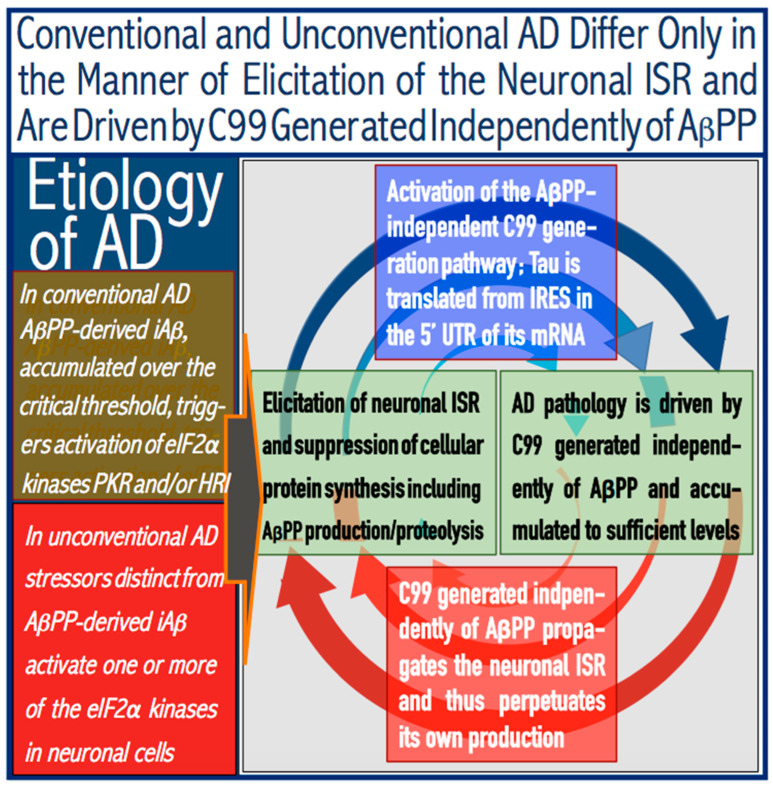
**Etiology of AD: Conventional and unconventional forms of the disease differ only in the manner of elicitation of the neuronal ISR and are driven by C99 produced in the autonomous AβPP-independent pathway**. *eIF2α:* Eukaryotic translation initiation factor 2*α*. *PKR and HRI:* Kinases capable of phosphorylating eIF2α at its Ser51 residue and thus eliciting the integrated stress response (ISR). *iAβ*: Intraneuronal Aβ accumulated either via cellular uptake of secreted extracellular Aβ or through retention of iAβ resulting from the processing of a fraction of AβPP-derived C99 on intraneuronal membranes. *C99*: C-terminal, 99 amino acid residues-long fragment of AβPP derived by proteolysis of the latter or produced in the AβPP-independent pathway. C99 can be further processed proteolytically by gamma-cleavage; this yields the Aβ and AβPP intracellular domain (AICD). AICD is known to interfere with various intraneuronal processes [154,155,156,157,158,159,160,161,162,163,164,165,166,167,168,169], but its involvement in AD remains to be fully elucidated. *IRES*: Internal ribosome entry site, which enables the internal cap-independent initiation of translation. In conventional AD, elicitation of the neuronal ISR is triggered by iAβ (“conventional stressor”) accumulated over the T1 threshold. In unconventional AD, stressors other than iAβ (“unconventional stressors”) elicit the neuronal ISR at levels of iAβ below the T1 threshold. In both forms of the disease, the neuronal ISR, once elicited, provides components essential for, and thus activates, the RNA-dependent mRNA amplification pathway. Human AβPP mRNA is eligible for the amplification process; in this case it occurs asymmetrically and yields severely 5′-truncated AβPP mRNA encoding C99. The efficiency of C99 production in this pathway is orders of magnitude greater than that of its production by AβPP proteolysis, and it rapidly accumulates. C99, accumulated over the T1 threshold, has two principal functions. The first is that it drives AD pathology. The second is that it propagates and sustains the neuronal ISR state and thus perpetuates both the AβPP-independent pathway of its own production and progression of the disease. Importantly, both principal actors of AD are produced in the disease in the ISR-compatible, cap-independent manner: C99 is translated from heavily modified 5′-truncated AβPP mRNA generated in the RNA-dependent AβPP mRNA amplification process, and tau protein is translated from an IRES positioned within the 5′UTR of its mRNA.

C99, in fact, has a dual function in AD: Not only does it drive the disease, but it also propagates the neuronal ISR state, and thus, it perpetuates its own production in the AβPP-independent pathway. Thus, when the latter is rendered operational, in the absence of therapeutic intervention the disease becomes self-sustaining and irreversible. To propagate the neuronal ISR conditions, one or more eIF2α kinases have to be maintained in the activated state. C99 can do it in more than one way. Thus, at sufficient levels, it was shown to cause ER stress [151]. ER stress activates PERK, a member of the family of eIF2α kinases. C99, at sufficient levels, was also shown to cause mitochondrial dysfunction [152]. This initiates the OMA1 to DELE1 signaling pathway and, consequently, activates HRI, another member of the family of eIF2α kinases. Sustainably activated eIF2α kinases keep eIF2α phosphorylated at its Ser51 amino acid residue and maintain the neuronal ISR state. This supports the operation of the AβPP-independent C99 generation pathway. These constantly escalating cycles constitute an engine, in fact the Engine that drives AD pathology. This is illustrated below in Figure 3.

It should be noted that in conventional AD, the AβPP-independent C99 generation pathway is self-sufficient from the moment it becomes operational. This is because at this point, AβPP-derived iAβ is over the T1 threshold and sustains the neuronal ISR state. When C99 also crosses the T1 threshold, it assumes propagation of ISR conditions. In contrast, this aspect is quite different in unconventional AD. In this category of the disease, the neuronal ISR is elicited, and the AβPP-independent C99 generation pathway is activated when levels of AβPP-derived iAβ are well below the ISR-triggering T1 threshold. In this case, there is a lag period between the activation of the AβPP-independent C99 generation pathway and the commencement of AD. Initially, until they cross the T1 threshold, levels of C99 produced independently of AβPP are insufficient to propagate the neuronal ISR, and the AβPP-independent production pathway is not self-sustaining. At this stage, if an unconventional stressor were withdrawn, the neuronal ISR state would reverse, the activity of the AβPP-independent C99 production pathway would cease, and no AD would occur. This is, potentially, what happens multiple times (possibly during viral or bacterial infections or during transient inflammations) in the lifetime of healthy individuals (who never develop AD or before they develop the disease). Only when levels of C99 produced independently of AβPP exceed the T1 threshold does the pathway become autonomous and self-sustainable, and AD commences.

It should also be noted that since the neuronal ISR state persists in the AD-affected neurons throughout the course of the disease, production of all proteins involved in the progression of AD must be unaffected by the neuronal ISR-mediated suppression of the global cellular protein synthesis. This is, in fact, a verifiable prediction. The case in point is tau protein, which comprises neurofibrillary tangles, the hallmark of the disease. Since it is evidently produced in the course of the disease, i.e., under ISR conditions, its production must be insensitive to the ISR-mediated inhibition of the regular cap-dependent initiation of translation. This, in fact, was recently shown to be the case. Synthesis of tau protein in AD is indeed unaffected by ISR conditions because its translation is regulated by an IRES element positioned within the 5′ UTR of its mRNA [153].

## 5. Aging-Associated Cognitive Decline: Origins, Duration, and Relationship to AD

The decisive event in the occurrence of conventional AD is the crossing of the T1 threshold by AβPP-derived iAβ; the sooner the T1 threshold is crossed, the sooner the disease commences. As discussed above, the timing of this event is influenced, to a large degree, by the rate of accumulation of AβPP-derived iAβ; the greater the latter, the shorter the former (inverse proportionality). Another factor that defines the timing of the T1 crossing by AβPP-derived iAβ is the extent of the T1 threshold. Indeed, at a given rate of accumulation of AβPP-derived iAβ, the greater the extent of the T1 threshold, the longer the timing of the commencement of AD (direct proportionality). iAβ has a propensity for aggregation, which is potentially capable of cellular damage. If the extent of the T1 threshold is sufficiently great, iAβ aggregation-caused neuronal damage may occur prior to the crossing of the T1 threshold, before elicitation of the neuronal ISR, activation of the AβPP-independent C99 generation pathway, and commencement of AD.

In the ACH2.0 paradigm, the neuronal damage caused by AβPP-derived iAβ at levels below the T1 threshold but above a certain level, designated as the T^0^ threshold, manifests as aging-associated cognitive decline, AACD [1,2,3,4,5,6,7,8,9,10,11]. The age of onset of AACD is greater than the age of onset of sporadic conventional AD [170,171]. This indicates that in the AACD-predisposed population, the extent of the T^0^ threshold (and the T1 threshold, which is always greater than T^0^ in AACD) is greater than the extent of the T1 threshold in the population predisposed to conventional sporadic AD. It follows that conventional sporadic AD is unlikely to be preceded by AACD. On the other hand, it also follows that the occurrence of AACD implies certain resistance to AD (due to the great extent of the T1 threshold), and that AACD patients, if they develop sporadic conventional AD at all, would develop it significantly later than the general age of the onset of sporadic AD (at about sixty-five years of age).

**Figure 4:**
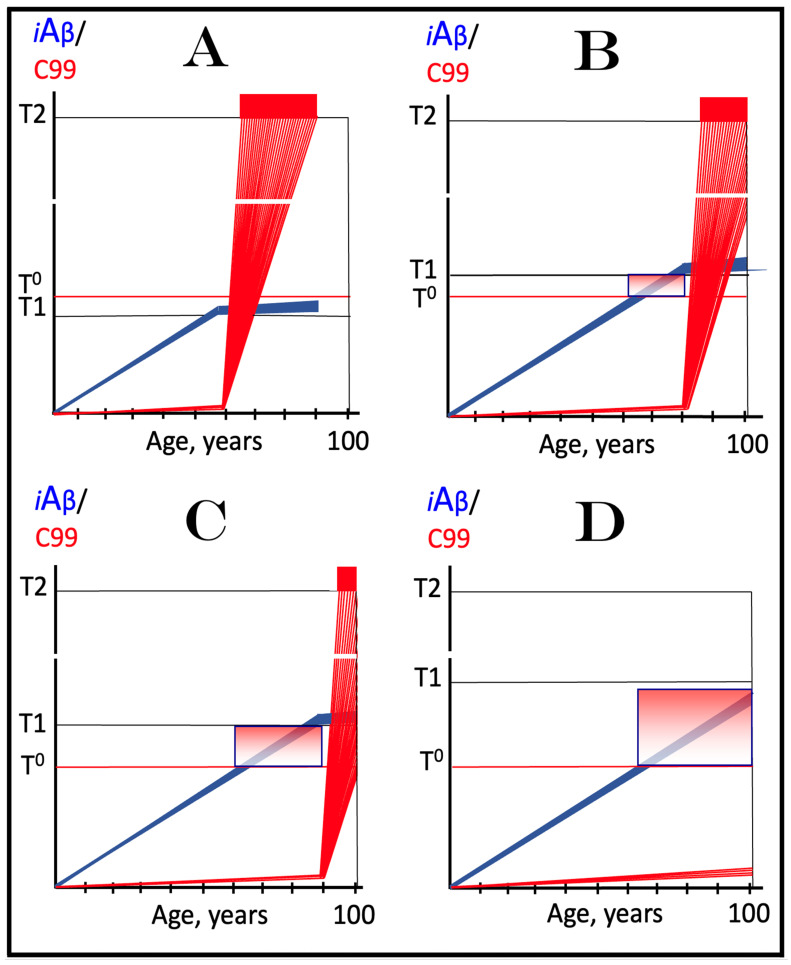
**Aging-associated cognitive decline (AACD): Etiology and relationship to Alzheimer’s disease**. *Vertical axis*: Relative levels of iAβ and C99. *Horizontal axis*: Age, in years. *Blue lines*: Dynamics of iAβ. *Red lines*: Dynamics of C99. **T^0^**: Threshold of cellular concentration of AβPP-derived *i*Aβ, which triggers neurodegeneration manifesting as AACD. **T1**: Threshold of intracellular concentration of iAβ or C99 that mediates elicitation of the neuronal integrated stress response, which, in turn, enables operation of the AβPP-independent C99 generation pathway and thus triggers the disease. **T2**: Threshold of intracellular concentration of C99 that triggers neuronal death. *Red boxes*: Range of intracellular concentrations of C99, dubbed “Apoptotic Zone”, associated with apoptosis or necroptosis of neuronal cells. *Pink boxes*: The duration and extent of AACD; it initiates when iAβ crosses the T^0^ threshold and morphs into AD when the T1 threshold is crossed (unless iAβ does not reach the T1 threshold, in which case AACD persists for the remaining portion of the lifetime). Thus, AACD can occur only if the extent of the T^0^ threshold is smaller than that of the T1 threshold. The following panels illustrate the effects of variable extents of the T1 threshold with all other parameters constant. Panel (**A**): The extent of the T^0^ threshold is greater than that of the T1 threshold. Conventional AD commences when iAβ crosses the T1 threshold, and no AACD occurs. Panel (**B**): The extent of the T^0^ is less than that of the T1 threshold. When iAβ crosses the former, AACD commences and morphs into AD with the crossing of the latter. Panel (**C**): The further elevation of the extent of the T1 threshold increases the duration of AACD. Panel (**D**): iAβ does not reach the T1 threshold within the lifetime of the individual. No AD occurs, but AACD commences when iAβ crosses the T^0^ threshold and persists for the remaining portion of the lifespan.

Origins of AACD, its duration, and its relationship to sporadic conventional AD are illustrated in Figure 4. Panel A of Figure 4 reflects a situation characteristic of the population predisposed to sporadic AD. The extent of the T1 threshold is below that of the T^0^ threshold. When AβPP-derived iAβ crosses the T1 threshold, the neuronal ISR is elicited, the AβPP-independent C99 generation pathway is activated, and AD commences. Simultaneously, under ISR conditions, the accumulation of AβPP-derived iAβ is suppressed. It will not reach the T^0^ threshold, and no AACD will occur. In panel B of Figure 4, the extent of the T1 threshold increases, and it is greater than that of the T^0^ threshold (in this Figure, the only variable parameter is the extent of the T1 threshold). AACD commences upon the crossing of the T^0^ threshold by AβPP-derived iAβ and morphs into conventional AD when the T1 threshold is reached and crossed. In panel C of Figure 4, the extent of the T1 threshold is increased even more. AACD commences at the same time as in panel B (since the extent of the T^0^ threshold is the same), but its duration is longer, and it morphs into conventional AD later. In panel D of Figure 4, the extent of the T1 threshold is so high that it is not reached by AβPP-derived iAβ within the lifetime of an individual, and no AD occurs. On the other hand, AACD commences with the T^0^ crossing and persists for the remaining lifetime of an individual.

Since AACD manifests before the neuronal ISR is elicited and the AβPP-independent C99 generation pathway is activated, this condition can be relatively easily addressed therapeutically. Strategies for the prevention of AACD and for its cure at symptomatic stages are discussed in Section 11 below.

## 6. Prospective AD Drugs in the ACH2.0 Paradigm: A Brief Overview

In the ACH2.0 paradigm, the key therapeutic target is clearly defined. It is the activity of the AβPP-independent C99 production pathway. This pathway is the essence, the active core of Alzheimer’s disease. With this pathway inoperative, neither conventional nor unconventional AD can occur. It follows that any effective AD drug must affect this pathway. To prevent AD, its activation must be precluded. To treat the disease, its activity must be suppressed. The present perspective considers three categories of potential AD drugs capable of accomplishing this either on their own or in combination. One such category comprises inhibitors of the neuronal integrated stress response. The neuronal ISR supplies components crucial for the activity of the AβPP-independent C99 production pathway and thus is essential for its operation. Suppression of the elicitation of the neuronal ISR would preclude activation of the AβPP-independent C99 production pathway and prevent the occurrence of AD. Reversal of the neuronal ISR state by ISR inhibitors would disable the AβPP-independent C99 generation pathway and arrest progression of the disease.

Another category of potential AD drugs includes agents capable of depleting or removing substances that elicit and/or sustain the neuronal ISR state and thus enable operation of the AβPP-independent C99 production pathway. In conventional AD, these substances include iAβ and C99. Indeed, conventional AD is triggered, via elicitation of the neuronal ISR, by iAβ accumulated over the T1 threshold. If the accumulation of iAβ is suppressed and the T1 crossing does not occur, no neuronal ISR would be elicited, no AβPP-independent C99 production pathway would be activated, and the conventional disease would be prevented. C99, on the other hand, not only drives both conventional AD and its unconventional counterpart but also propagates the neuronal ISR state and perpetuates its own production in the AβPP-independent pathway. In conventional AD, sustained depletion of C99 and iAβ to levels below the T1 threshold would reverse the neuronal ISR state, disable the AβPP-independent C99 generation pathway, and effectively treat the disease. In unconventional AD, the neuronal ISR is elicited, and the AβPP-independent C99 production pathway is activated by unconventional stressors at levels of iAβ well below the T1 threshold. Therefore, both prevention and treatment of this form of AD entail not only the sustained depletion of C99 but, potentially, also that of an unconventional stressor.

The third category of potential AD drugs consists of agents interfering directly with mechanistic components of the AβPP-independent C99 production pathway. This approach, however, is challenging. As described elsewhere [12] and summarized below, the most plausible molecular mechanism underpinning the activity of the AβPP-independent C99 production pathway is asymmetric RNA-dependent amplification of AβPP mRNA, resulting in a severely 5′-truncated molecule encoding, as its primary translation product, the C100 fragment of AβPP, which is subsequently processed into C99. The challenge arises from the fact that mammalian RNA-dependent mRNA amplification is a physiological mechanism widely employed in processes such as erythropoiesis [172,173] and the deposition of the extracellular matrix [174]. Therefore, any systemic interference with this process would, potentially, be highly deleterious. On the other hand, specific inhibition of this mechanism via the interference with its AβPP mRNA substrate could be immensely promising. This category of potential AD drugs comprises agents that either interfere with the amplification of human AβPP mRNA or make it ineligible for the amplification process. Such agents would be equally efficient for both conventional and unconventional AD and are described in Section 22, Section 23, Section 24 and Section 25 below.

## 7. Inhibitors of the Neuronal ISR as Potential AD Drugs

In the ACH2.0 paradigm, in both conventional and unconventional forms of AD, the direct cause of the disease is the sustained neuronal ISR state. It provides components that are essential for operation of the AβPP-independent C99 production pathway. Without it, the pathway is inoperative and the disease cannot occur. It stands to reason, therefore, that the prevention of the neuronal ISR would preclude the occurrence of AD, and the reversal of the neuronal ISR state would disable the AβPP-independent C99 generation pathway and would thus constitute an effective treatment for both conventional and unconventional forms of AD. The simplest way to accomplish this is to administer ISR inhibitors.

As discussed below, to exert their therapeutic effect, both in the prevention and treatment of AD, ISR inhibitors would have to be administered long-term. Small-molecule ISR inhibitors suitable for such applications, such as ISRIB, are available. Their effect is systemic rather than tissue- or cell-type-specific. However, the long-term systemic inhibition of the ISR appears not to be feasible. The ISR phenomenon constitutes a major physiological survival tool, and its long-term systemic suppression is bound to be detrimental. This notion is supported by the observations made in eIF2α Ser51Ala KI mice [175]. In this transgenic mouse model, the serine residue at position 51 of eIF2α is replaced by alanine. However, whereas Ser51 can be phosphorylated, thus triggering the ISR, Ala51 of eIF2α cannot be phosphorylated, and the ISR cannot be elicited. Such transgenically modified mice can be regarded, therefore, as a model for long-term systemic inhibition of the ISR. Results obtained with such a model confirm the predicted deleterious effect of the long-term administration of ISR inhibitors. Indeed, mice with homozygous eIF2α Ser51Ala modification survived only eighteen hours following their birth [175]. Thus, systemic inhibitors of the ISR are highly unlikely to be suitable for long-term applications. On the other hand, cell-type-specific ISR inhibitors in general and neuron- or only AD-affected neuron-specific ISR inhibitors (or general ISR inhibitors that can be delivered in a cell-type-specific manner to neurons or even only to the AD-affected neurons) in particular hold a substantial promise as therapeutic agents for AD. Their anticipated effects in the prevention and treatment of AD are discussed in the following sections.

## 8. Suppression of the Neuronal ISR in the Prevention and Treatment of Conventional AD

### 8.1. Suppression of the Neuronal ISR in the Prevention of Conventional AD

**Figure 5:**
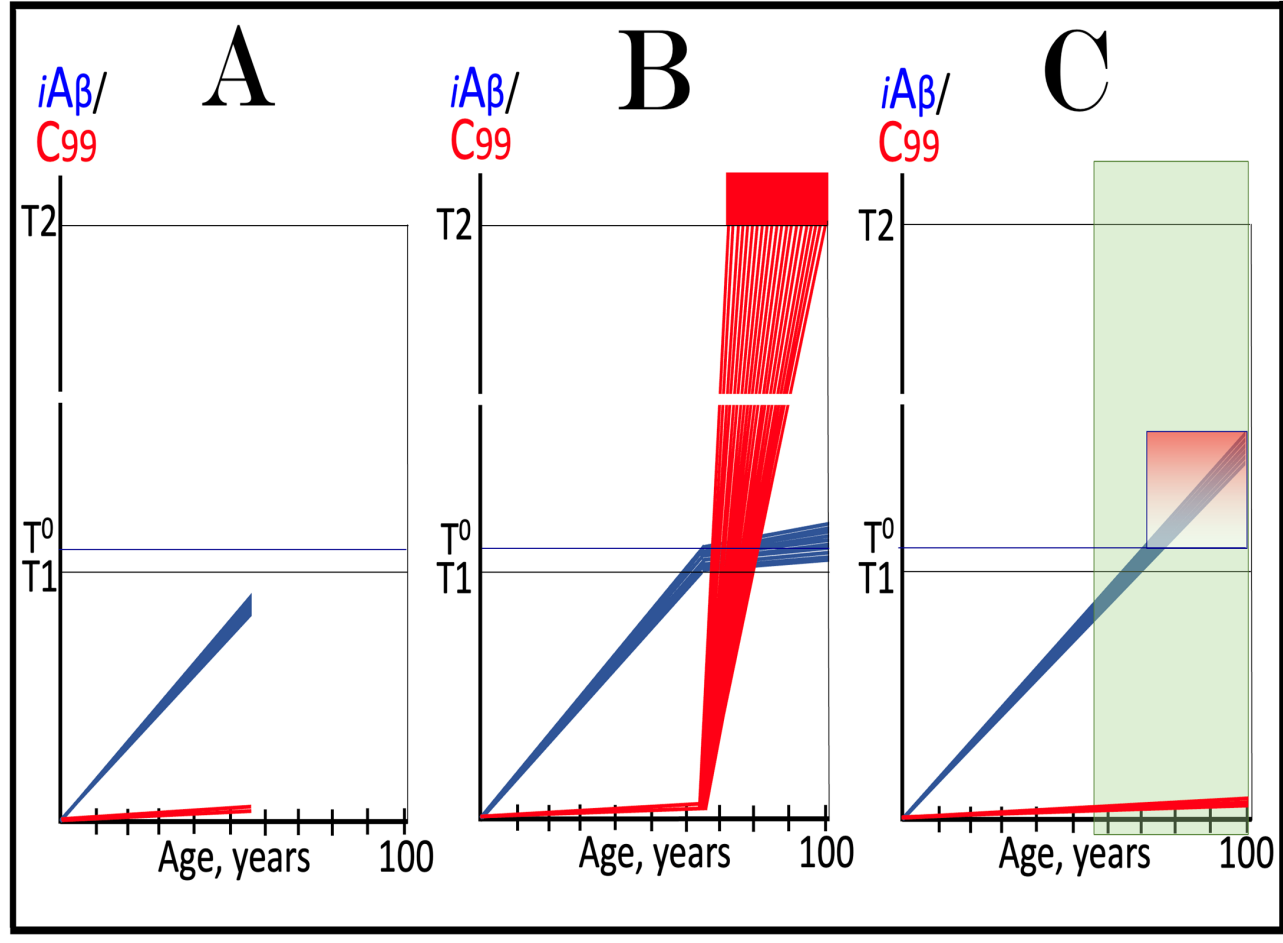
**Suppression of the neuronal ISR in the prevention of conventional AD**. *Vertical axis*: Relative levels of iAβ and C99. *Horizontal axis*: Age, in years. *Blue lines*: Dynamics of iAβ. *Red lines*: Dynamics of C99. **T^0^**: Threshold of cellular concentration of AβPP-derived *i*Aβ, which triggers neurodegeneration manifesting as AACD. **T1**: Threshold of intracellular concentration of iAβ or C99 that mediates elicitation of the neuronal integrated stress response, which, in turn, enables operation of the AβPP-independent C99 generation pathway and thus triggers the disease. **T2**: Threshold of intracellular concentration of C99 that triggers neuronal death. *Red box*: Range of intracellular concentrations of C99, dubbed “Apoptotic Zone”, associated with apoptosis or necroptosis of neuronal cells. *Pink box*: The duration and extent of AACD. *Green box*: Duration of the ISR suppression treatment. Panel (**A**): The initial state of levels of iAβ and C99 in the affected neurons at the commencement of the ISR suppression treatment. iAβ has not yet reached the T1 threshold. Neither the neuronal ISR has been elicited nor the AβPP-independent C99 production pathway activated. Panel (**B**): Evolution of the initial states of iAβ and C99 in the absence of the ISR suppression treatment. iAβ crosses the T1 threshold. PKR and/or HRI are activated, eIF2α phosphorylated, and the neuronal IRS is elicited. The production of iAβ in the AβPP proteolytic pathway and its accumulation are suppressed. Concurrently, under ISR conditions, the AβPP-independent C99 generation pathway is activated, and AD commences and progresses. Panel (**C**): Evolution of the initial states of iAβ and C99 under the ISR suppression treatment. AβPP-derived iAβ crosses the T1 threshold. Under the treatment, the neuronal ISR cannot be elicited, and the AβPP-independent C99 generation pathway remains inoperative. Accumulation of AβPP-derived iAβ and C99 continues at the pre-treatment rates. Their levels do not reach the AD pathology-driving range, and the disease does not occur for the duration of the treatment. When iAβ crosses the T^0^ threshold, AACD commences and persists for the duration of the treatment.

As discussed above, if the activation of the AβPP-independent C99 generation pathway is precluded, AD would be prevented. This pathway can operate only under the neuronal ISR conditions. It follows that preventing the elicitation of neuronal ISR equals preventing the disease. Effects of suppression of the neuronal ISR in the prevention of conventional AD are illustrated schematically in Figure 5. Panel A of Figure 5 depicts the initial states of the levels of iAβ and C99 at the commencement of administration of ISR inhibitors. At this point, the levels of AβPP-derived iAβ have not yet reached the T1 threshold, the neuronal ISR has not been elicited, the AβPP-independent C99 generation pathway has not been activated, and the individual is healthy.

Panel B of Figure 5 shows the evolution of the initial states of iAβ and C99 in the absence of the ISR suppression treatment. In this scenario, the accumulation of AβPP-derived iAβ continues, and it reaches and crosses the T1 threshold. PKR and/or HRI kinases are activated, eIF2α is phosphorylated, and the neuronal IRS is elicited. Under neuronal ISR conditions, the production of iAβ in the AβPP proteolytic pathway is suppressed, and so is its accumulation. Simultaneously, the AβPP-independent C99 generation pathway is initiated. AD commences and progresses; when C99 crosses the T2 apoptotic threshold in a sufficient fraction of the neurons, the disease reaches its end stage.

Panel C of Figure 5 shows the anticipated effects of the neuronal ISR suppression treatment. AβPP-derived iAβ reaches and crosses the T1 threshold. Under the treatment, the neuronal ISR cannot be elicited, and the AβPP-independent C99 generation pathway remains inoperative. Accumulation of AβPP-derived iAβ and C99 continues unhindered. Their levels do not reach the AD pathology-driving range, and the disease does not occur for the duration of the treatment. If, however, AβPP-derived iAβ were to cross the T^0^ threshold, AACD would commence and persist for the duration of the treatment. Prevention of AD requires a lifelong duration of the treatment. If the treatment is discontinued, the neuronal ISR would be elicited, and AD would commence.

### 8.2. Suppression of the Neuronal ISR in the Treatment of Conventional AD

The logic of the preceding section also applies to the treatment of conventional AD. Indeed, the goal of such a treatment is suppression or cessation of the operation of the AβPP-independent C99 generation pathway. This pathway is operational only under the neuronal ISR conditions; indeed, the latter provides the former with its essential components. It follows that the reversal of the neuronal ISR state would deprive the AβPP-independent C99 generation pathway of its vital components and thus would disable it. Consequently, with the influx of the driver of AD pathology (i.e., C99) interrupted, the progression of the disease would cease.

**Figure 6:**
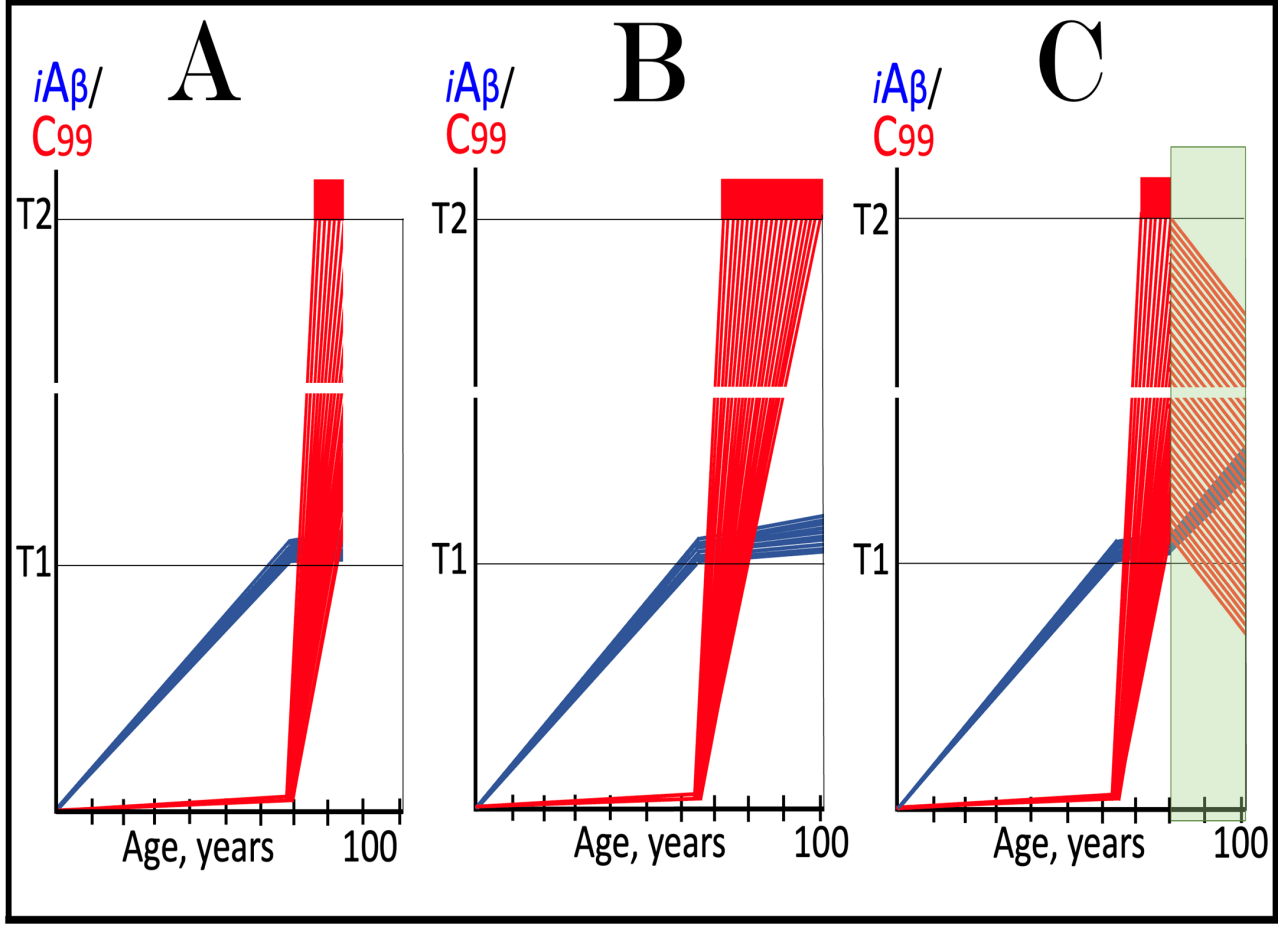
**Suppression of the neuronal ISR in the treatment of conventional AD**. *Vertical axis*: Relative levels of iAβ and C99. *Horizontal axis*: Age, in years. *Blue lines*: Dynamics of iAβ. *Red lines*: Dynamics of C99. **T1**: Threshold of intracellular concentration of iAβ or C99 that mediates elicitation of the neuronal integrated stress response, which, in turn, enables operation of the AβPP-independent C99 generation pathway and thus triggers the disease. **T2**: Threshold of intracellular concentration of C99 that triggers neuronal death. *Red box*: Range of intracellular concentrations of C99, dubbed “Apoptotic Zone”, associated with apoptosis or necroptosis of neuronal cells. *Green box*: Duration of the ISR suppression treatment. Panel (**A**): The initial state of levels of iAβ and C99 in the affected neurons at the commencement of the ISR suppression treatment. AβPP-derived iAβ has crossed the T1 threshold. The neuronal ISR has been elicited, and the production and accumulation of iAβ have been suppressed. Concurrently, the neuronal ISR provided components essential for the AβPP-independent C99 generation pathway and enabled its operation. Levels of C99 have rapidly increased. They crossed the T2 threshold in a fraction of the affected neurons, and AD symptoms have manifested. Panel (**B**): Evolution of the initial states of iAβ and C99 in the untreated AD patient. The accumulation of C99 and progression of the disease proceed unimpeded; the disease reaches its end stage. Panel (**C**): Evolution of the initial states of iAβ and C99 under the ISR suppression treatment. Under the treatment, the neuronal ISR state is reversed. With its essential components no longer supplied, the AβPP-independent C99 generation pathway is disabled. The influx of C99 is stopped, and the progression of the disease is arrested for the duration of the treatment. In the absence of the ISR, the operation of the AβPP proteolytic pathway is enabled, and accumulation of AβPP-derived iAβ resumes at the pre-T1 crossing rate but its levels do not reach the AD pathology-driving range. The disease will not recur for the duration of the treatment.

This therapeutic strategy is presented schematically in Figure 6. The initial state of the levels of iAβ and C99 at the commencement of the neuronal ISR suppression treatment is shown in panel A of Figure 6. At this point, AβPP-derived iAβ has already crossed the T1 threshold. The neuronal ISR has been elicited and, as a result, the production and accumulation of iAβ have been suppressed. Concurrently, the neuronal ISR provided components essential for the AβPP-independent C99 generation pathway and thus enabled its operation. Levels of C99 have rapidly increased. They crossed the apoptotic T2 threshold in a fraction of the neurons, and AD symptoms have manifested.

Panel B of Figure 6 depicts the evolution of the initial state in the untreated patient. The disease progresses unimpeded. C99 crosses the T2 threshold in more neurons, and eventually the disease reaches its end stage. Panel C of Figure 6 depicts anticipated outcomes of the neuronal ISR suppression treatment. Under the treatment, the neuronal ISR state is reversed. With its essential components no longer supplied, the AβPP-independent C99 generation pathway is disabled and its operation ceases. This stops the influx of the driver of AD pathology, i.e., C99, and arrests the progression of the disease for the duration of the treatment. In the absence of the ISR, the operation of the AβPP proteolytic pathway is enabled, and accumulation of AβPP-derived iAβ resumes at the pre-T1 crossing rate. It will not, however, reach the AD pathology-driving range. It should be emphasized that, to be effective, the treatment should continue for the remaining portion of the lifespan. If it is terminated, the neuronal ISR would be re-elicited by over-the-T1 iAβ, and the disease would recur.

## 9. Suppression of the Neuronal ISR in the Prevention and Treatment of Unconventional AD

### 9.1. Suppression of the ISR in the Prevention of Unconventional AD

As discussed above, two features differentiate unconventional AD from its conventional counterpart. One is that the neuronal ISR is elicited by stressors other than AβPP-derived iAβ. Another feature specific to unconventional AD is that the neuronal ISR, which triggers the disease, is always elicited at the levels of AβPP-derived iAβ below the T1 threshold. Despite these differences, the mechanisms that cause and drive AD are identical in both conventional and unconventional forms of the disease: In both, it is caused by the sustained neuronal ISR, which supplies the essential components and thus enables operation of the AβPP-independent C99 generation pathway, and is driven by C99 produced independently of AβPP. Therefore, the long-term suppression of the neuronal ISR would be as effective in the prevention and treatment of unconventional AD as in its conventional counterpart.

**Figure 7:**
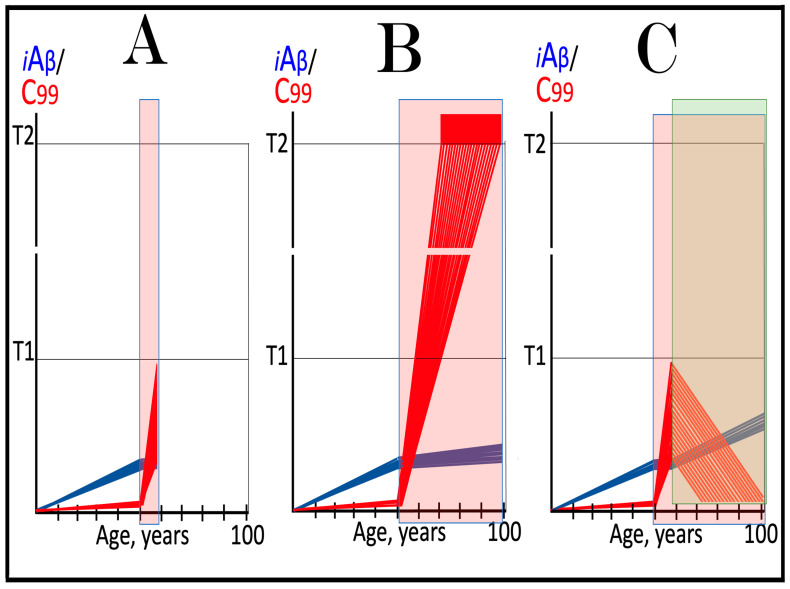
**Suppression of the neuronal ISR in the prevention of unconventional AD**. *Vertical axis*: Relative levels of iAβ and C99. *Horizontal axis*: Age, in years. *Blue lines*: Dynamics of iAβ. *Red lines*: Dynamics of C99. **T1**: Threshold of intracellular concentration of iAβ or C99 that mediates elicitation of the neuronal integrated stress response, which, in turn, enables operation of the AβPP-independent C99 generation pathway and thus triggers the disease. **T2**: Threshold of intracellular concentration of C99 that triggers neuronal death. *Red box*: Range of intracellular concentrations of C99, dubbed “Apoptotic Zone”, associated with apoptosis or necroptosis of neuronal cells. *Pink box*: The duration of the occurrence of unconventional stressors (as defined in the main text) at concentrations sufficient to elicit the neuronal ISR. *Green box*: Duration of the ISR suppression treatment. Panel (**A**): The initial state of levels of iAβ and C99 in the affected neurons at the commencement of the ISR suppression treatment. The neuronal ISR has been elicited by an unconventional stressor at the levels of iAβ below the T1 threshold, and the production and accumulation of AβPP-derived iAβ have been suppressed. Concurrently, the AβPP-independent C99 generation pathway has been activated. C99 produced independently of AβPP has rapidly accumulated but has not yet reached the T1 threshold. Panel (**B**): Evolution of the initial state in the absence of the treatment. Accumulation of C99 produced independently of AβPP continues unimpeded. When it crosses the T1 threshold, the AβPP-independent C99 generation pathway is rendered autonomous and AD commences and progresses. Panel (**C**): Evolution of the initial states of iAβ and C99 under the ISR suppression treatment. Under the treatment, the neuronal ISR state is reversed. Accumulation of AβPP-derived iAβ resumes at the pre-ISR elicitation rate and will not reach the T1 threshold. Supply of the essential components of the AβPP-independent C99 generation pathway ceases, the pathway is disabled, and levels of C99 decline. They would not reach the AD pathology-causing range, and the disease would not occur for the duration of the treatment.

The effect of suppression of the neuronal ISR in the prevention of unconventional AD is illustrated schematically in Figure 7. Panel A of Figure 7 depicts the initial states of the levels of iAβ and C99 at the commencement of administration of ISR inhibitors. At this point, the neuronal ISR has been elicited by an unconventional stressor at the levels of AβPP-derived iAβ well below the T1 threshold. As a result, the production and accumulation of AβPP-derived iAβ have been suppressed. Concurrently, under the neuronal ISR state, the AβPP-independent C99 generation pathway has been activated. C99 produced independently of AβPP has rapidly accumulated but has not yet reached the T1 threshold.

Panel B of Figure 7 depicts the evolution of the initial state in the untreated individual. The rapid accumulation of C99 produced independently of AβPP continues unimpeded. When it crosses the T1 threshold, the AβPP-independent C99 generation pathway becomes self-sustainable and AD commences. When C99 crosses the T2 threshold in a sufficient neuronal fraction, the disease reaches its end stage. Panel C of Figure 7 shows the evolution of the initial state resulting from the neuronal ISR suppression therapy. The neuronal ISR state is reversed. The AβPP proteolytic pathway becomes operational, and the accumulation of AβPP-derived iAβ resumes at the pre-ISR elicitation rate. At the same time, the reversal of the ISR state ceases the supply of the essential components of the AβPP-independent C99 generation pathway, and the pathway is disabled. As a result, levels of C99 decline. With the AβPP-independent C99 production pathway inoperative, the disease will not occur for the duration of the treatment.

### 9.2. Suppression of the ISR in the Treatment of Unconventional AD

**Figure 8:**
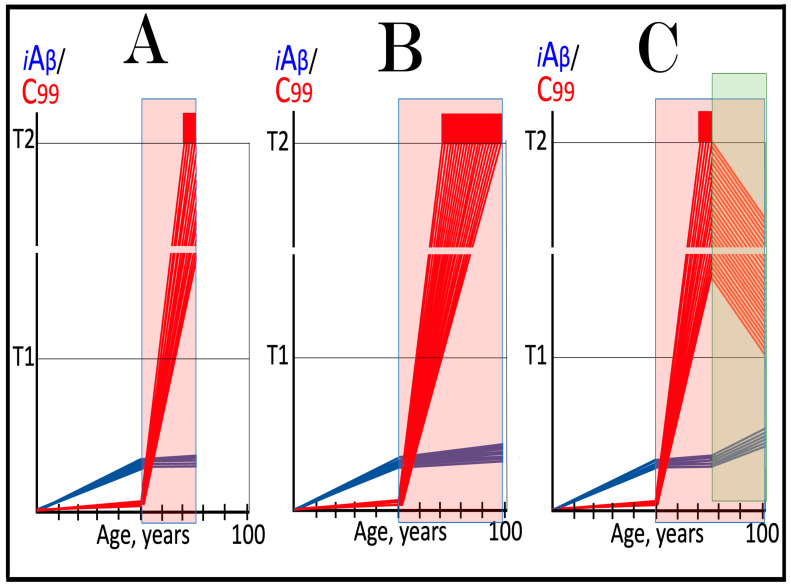
**Suppression of the neuronal ISR in the treatment of unconventional AD**. *Vertical axis*: Relative levels of iAβ and C99. *Horizontal axis*: Age, in years. *Blue lines*: Dynamics of iAβ. *Red lines*: Dynamics of C99. **T1**: Threshold of intracellular concentration of iAβ or C99 that mediates elicitation of the neuronal integrated stress response, which, in turn, enables operation of the AβPP-independent C99 generation pathway and thus triggers the disease. **T2**: Threshold of intracellular concentration of C99 that triggers neuronal death. *Red box*: Range of intracellular concentrations of C99, dubbed “Apoptotic Zone”, associated with apoptosis or necroptosis of neuronal cells. *Pink box*: The duration of the occurrence of unconventional stressors (as defined in the main text) at concentrations sufficient to elicit the neuronal ISR. *Green box*: Duration of the ISR suppression treatment. Panel (**A**): The initial state of levels of iAβ and C99 in the affected neurons at the commencement of the ISR suppression treatment. The neuronal ISR has been unconventionally elicited at the levels of AβPP-derived iAβ below the T1 threshold. Consequently, production and accumulation of AβPP-derived iAβ have been suppressed. Concurrently, the AβPP-independent C99 generation pathway has been unconventionally activated. Levels of C99 have rapidly increased and crossed the T1 threshold. The AβPP-independent C99 production pathway became self-sufficient and AD commenced. When C99 crossed the T2 threshold in a fraction of the neurons, AD symptoms manifested. Panel (**B**): Evolution of the initial state in the untreated patient. The accumulation of C99 produced in the AβPP-independent pathway continues unimpeded, and the disease progresses. Panel (**C**): Evolution of the initial state under the ISR suppression treatment. The neuronal ISR state is reversed. The AβPP proteolytic pathway becomes operational, and accumulation of AβPP-derived iAβ resumes at the pre-ISR elicitation rate. Components essential for the operation of the AβPP-independent C99 generation pathway are no longer supplied, and the pathway is disabled. Levels of C99 are steadily decreasing. The progression of AD is arrested, and the disease will not recur for the duration of the treatment.

The effect of suppression of the neuronal ISR in the treatment of unconventional AD is illustrated schematically in Figure 8. Panel A of Figure 8 shows the initial state of the levels of iAβ and C99 at the commencement of administration of the ISR suppression therapy. At this stage, the neuronal ISR has been unconventionally elicited, and the AβPP-independent C99 generation pathway has been unconventionally activated at the levels of AβPP-derived iAβ well below the T1 threshold. As a result, the AβPP proteolytic pathway and accumulation of AβPP-derived iAβ have been suppressed. At the same time, generation of C99 in the AβPP-independent pathway has been activated. Its levels have rapidly increased and crossed the T1 threshold. The AβPP-independent C99 production pathway became self-sufficient and AD commenced. C99 continued its accumulation, crossed the T2 threshold in a fraction of the neurons, and AD symptoms manifested.

Panel B of Figure 8 depicts the evolution of the initial state in the untreated patient. The accumulation of C99 produced in the AβPP-independent pathway continues unimpeded, and the disease progresses. When the T2 threshold is crossed in a sufficient fraction of the neurons, the disease reaches its end stage. Panel C of Figure 8 shows the evolution of the initial state in the patient undergoing the neuronal ISR suppression therapy. In this scenario, the neuronal ISR state is reversed. The AβPP proteolytic pathway becomes operational, and accumulation of AβPP-derived iAβ resumes at the pre-ISR elicitation rate. Components essential for the operation of the AβPP-independent C99 generation pathway are no longer supplied, and the pathway is disabled. Levels of C99 are steadily decreasing. The progression of AD is arrested, and the disease would not recur for the duration of the treatment.

It should be emphasized that the above outcomes require continuous treatment for the remaining portion of the patient’s lifetime. If the treatment is discontinued, the neuronal ISR would be unconventionally re-elicited, the AβPP-independent C99 production pathway re-activated, and the disease would recur. It should also be emphasized that the currently available systemic ISR inhibitors are, apparently, not suitable for long-term treatment. For successful ISR suppression therapy, ISR inhibitors should be either neuron-specific or be delivered specifically to the neurons, or, even better, to the AD-affected neurons.

## 10. Targeted Degradation of iAβ and Aβ Segment of C99 in the Prevention of Conventional AD

### 10.1. Icelandic AD-Preventing Mutation and Flemish FAD-Causing Mutation as Guides to the Prevention of Conventional AD

As stated above, in the ACH2.0 paradigm, the conventional form of AD is triggered by iAβ accumulated over the critical, ISR-eliciting, T1 threshold. It follows that in the framework of the ACH2.0, the incidence of the conventional disease is a function of the rate of accumulation of iAβ and of the extent of the T1 threshold, and that manipulation of these parameters can either accelerate or delay/prevent the occurrence of conventional AD. This notion is validated by the outcomes of naturally occurring Aβ and presenilin mutations. Indeed, without a single exception, all mutations causing early-onset familial AD [176] either accelerate the rate of accumulation of iAβ or reduce the extent of the T1 threshold, whereas the naturally occurring mutation that protects from both AD and AACD decreases the rate of the accumulation of iAβ [1,2,3,4,5,6,7,8,9,10,11]. Two representative Aβ mutations, as well as the molecular mechanisms through which they accomplish their outcomes, are discussed below.

One such mutation is known as the Icelandic Aβ mutation, and it exerts its effect by increasing the efficiency of BACE1 cleavage within iAβ [177,178]. The primary BACE1 cleavage occurs at the so-called “β-site”, namely between the Met671 and Asp672 of AβPP, thus forming the N-terminus of C99 and of Aβ. BACE1 also possesses a secondary activity, which cleaves at a site ten amino acids downstream from the β-site; this secondary cleavage location is designated the β’-site. The Icelandic mutation, which occurs between the β- and β’-sites, markedly increases the efficiency of BACE1 cleavage at the latter. This cleavage prevents the formation of C99 and degrades already formed C99 and iAβ. As a result, less iAβ is formed and more iAβ (and its precursors AβPP and C99) is degraded. Consequently, its influx decreases, its efflux increases, and its rate of accumulation declines. The T^0^ and T1 crossings do not occur within the individual’s lifetime or occur later than in wild-type AβPP carriers. Accordingly, both AD and AACD are either prevented or delayed.

Another naturally occurring mutation of interest occurs at the position Aβ21 and is designated as the “Flemish” Aβ mutation [179]. This mutation affects the major activity of BACE2. As suggested by its designation, BACE2 is capable of cleaving AβPP at the β-site. This, however, is its minor activity. Its primary and major activity is cleavages at the Aβ19 and Aβ20 sites. Physiologically, this activity controls levels of iAβ and maintains its homeostasis. Being in the proximity of the major BACE2 cleavage sites, the Flemish Aβ mutation interferes with and substantially decreases the efficiency of the primary activity of the enzyme. As a result, more AβPP is being processed into C99, and C99 into iAβ, and less of the already formed iAβ is being degraded. Consequently, the influx of the latter rises, whereas its efflux decreases, and its rate of accumulation increases. The T1 threshold is reached sooner, and early-onset conventional AD, starting at thirty-five years of age [180,181,182,183,184,185,186], ensues.

It should be noted that the Flemish mutation is a representative of a type of FAD mutation that increases the influx of iAβ. This type also includes the Swedish Aβ mutation [187] and certain PSEN mutations [188]. These two mutations result in the same outcome as seen in carriers of the Flemish mutation, albeit via a distinctly different mechanism. Both mutations increase the proportion of C99 undergoing gamma-cleavages on the intraneuronal membranes. Since Aβ resulting from these gamma-cleavages is retained intraneuronally as iAβ, its influx is greater than in individuals carrying wild-type AβPP genes. Consequently, its levels reach the T1 threshold sooner, resulting in the early-onset disease.

**Figure 9:**
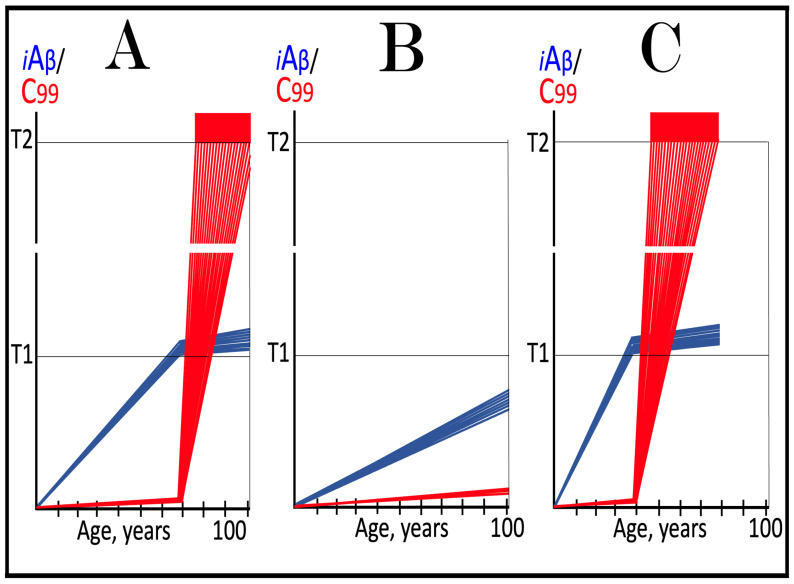
**FAD-causing mutations as guides to the prevention of conventional AD**. *Vertical axis*: Relative levels of iAβ and C99. *Horizontal axis*: Age, in years. *Blue lines*: Dynamics of iAβ. *Red lines*: Dynamics of C99. **T1**: Threshold of intracellular concentration of iAβ or C99 that mediates elicitation of the neuronal integrated stress response, which, in turn, enables operation of the AβPP-independent C99 generation pathway and thus triggers the disease. **T2**: Threshold of intracellular concentration of C99 that triggers neuronal death. *Red boxes*: Range of intracellular concentrations of C99, dubbed “Apoptotic Zone”, associated with apoptosis or necroptosis of neuronal cells. Panel (**A**): Etiology of late-onset conventional AD (“sporadic AD”). The rate of accumulation of AβPP-derived iAβ is such that it crosses the T1 threshold in the late sixties. This triggers activation of PKR and/or HRI kinases, phosphorylation of eIF2α, and elicitation of the neuronal ISR. Under ISR conditions, production and accumulation of iAβ are suppressed along with that of the bulk of cellular proteins. Concurrently, the ISR state enables the AβPP-independent generation of C99. It remains largely intact due to the deficit of gamma-secretase, rapidly accumulates, and drives AD pathology. Panel (**B**): Icelandic Aβ mutation reduces the rate of iAβ accumulation and thus protects from conventional AD. Icelandic mutation augments BACE1-mediated intra-Aβ cleavage at the β’-site. This cleavage prevents the formation of C99 and degrades already formed C99 and iAβ. As a result, less iAβ is formed and more iAβ (and its precursors AβPP and C99) is degraded. Consequently, the influx of iAβ decreases, its efflux increases, and its rate of accumulation declines. The T1 threshold is either not crossed or the crossing is delayed. Consequently, conventional AD is either prevented or delayed. Panel (**C**): Flemish Aβ mutation accelerates accumulation of iAβ and thus causes early-onset AD (FAD). Flemish mutation occurs in the proximity of intra-Aβ sites of cleavage by BACE2 and substantially decreases the efficiency of these cleavages. More AβPP is being processed into C99 and C99 into iAβ, and less of the already formed iAβ is being degraded. Consequently, the influx of the latter rises, whereas its efflux decreases, and its rate of accumulation increases. The T1 threshold is reached sooner, and early-onset conventional AD ensues.

The mechanisms and outcomes of the Icelandic and Flemish Aβ mutations are illustrated diagrammatically in Figure 9. Panel A of Figure 9 depicts processes leading to late-onset conventional AD. AβPP-derived iAβ steadily accumulates and crosses the T1 threshold. PKR and/or HRI kinases are activated and the integrated stress response is elicited. The accumulation of iAβ is suppressed, but the AβPP-independent production of C99 is activated. When C99 crosses the T1 threshold, conventional AD commences and progresses. In panel B of Figure 9, the Icelandic Aβ mutation substantially reduces the rate of accumulation of iAβ. It crosses neither the T^0^ nor T1 thresholds within the lifetime of the individual. Neither conventional AD nor AACD occur. In panel C of Figure 9, the Flemish Aβ mutation significantly accelerates the rate of accumulation of AβPP-derived iAβ. The T1 threshold is crossed much sooner than in wild-type, resulting in early-onset conventional AD.

### 10.2. ACH-Based AD Drugs Are Completely Impotent in the Treatment of AD but Can Delay or Prevent the Conventional Disease by Emulating the Action of the Protective AD Mutation

The initial theory of AD, the Amyloid Cascade Hypothesis (ACH), stipulated that the disease is caused and driven by extracellular Aβ derived from the proteolysis of AβPP and secreted from the cell. This notion inspired the creation of an array of potential AD drugs (ACH-based drugs) designed to reduce levels of extracellular Aβ. They include agents, such as monoclonal Aβ antibodies, which directly remove or sequester extracellular Aβ. They also include inhibitors of BACE1, which reduce the overall production of AβPP-derived Aβ, and, consequently, its secretion. As discussed above, these drugs succeeded spectacularly when trialed in transgenic mouse models overexpressing Aβ. However, as mentioned above and further discussed below, the AβPP-independent C99 production pathway is inoperative in these model systems. When the same drugs were trialed in symptomatic AD, where the AβPP-independent C99 generation pathway is operative, they failed no less spectacularly than they succeeded in model systems.

ACH2.0 provides a rational explanation of the contrasting outcomes outlined above. In animal models, expression of numerous, in some cases over a hundred, human AβPP transgenes results in vast overexpression and secretion of Aβ. A fraction of extracellular Aβ is taken up by neurons via mechanisms described in the preceding sections. In addition, a fraction of C99 is processed on the intracellular membranes; this fraction is retained intraneuronally. Due to the vastness of the production of exogenous AβPP, iAβ rapidly accumulates, crosses the T1 threshold, and triggers elicitation of the neuronal integrated stress response. Despite the neuronal ISR state, however, for the reasons elaborated below, the AβPP-independent C99 production pathway remains inoperative, and AD does not develop. A degree of neurodegeneration that is seen in these models and was previously interpreted as an AD symptom is due, in fact, to the ISR-caused global reduction in neuronal protein synthesis, and so are the observed cognitive impairments, such as defects in learning, in memory formation, and in neuronal plasticity, all requiring new neuronal protein synthesis [189,190,191,192,193,194,195,196,197,198,199,200,201,202,203,204]. These symptoms, therefore, result not from AD but rather from the sustained neuronal ISR. Prevention of ISR in these models, by ISR inhibitors such as ISRIB, prevents these symptoms, and inhibition of neuronal ISR after it occurs abrogates these symptoms. ACH-based AD drugs reduce levels of extracellular Aβ (and those of intraneuronally retained Aβ in the case of BACE1 inhibitors) and thus decrease or reverse the rate of iAβ accumulation. When its levels fall to those below T1, the ISR state is reversed, neuronal protein synthesis is restored, and symptoms are reduced or rescinded [205,206,207,208,209,210,211,212,213].

In contrast, in human AD patients, the iAβ-mediated neuronal ISR activates the AβPP-independent C99 production pathway, which drives AD pathology. This pathway is autonomous and self-sustaining. Reduction in iAβ, even to the levels below the T1 threshold, is completely inconsequential at this point since the neuronal ISR state is sustained by C99 generated independently of AβPP. Therefore, administration of ACH-based AD drugs would be patently futile in the neurons with the operational AβPP-independent C99 generation pathway. It could be argued that the effects of some ACH-based drugs, namely lecanemab and donanemab, observed in early symptomatic AD [214,215,216,217,218], contradict this notion. There is, however, no contradiction. Both drugs deplete extracellular Aβ and are protective only in the neurons where iAβ has not yet crossed the T1 threshold (since drugs were administered at very early stages of AD, defined by a new biomarker not available in preceding clinical trials of AD, a marginal fraction of the affected neurons was still udder-T1) and the AβPP-independent C99 production pathway has not yet been activated. This is the reason why their protective effect was only marginal. Moreover, it can be stated with certainty that any ACH-based AD drug (i.e., extracellular Aβ-depleting drug) would elicit the same effect as lecanemab and donanemad if administered at sufficiently early stages of AD. Thus, the bottom line is that ACH-based AD drugs are completely impotent in the treatment of AD but can delay or prevent the conventional disease if administered prior to the T1 crossing by emulating the action of the protective AD mutation.

### 10.3. A Single Transient Targeted iAβ Degradation Treatment Can Confer Lifelong Protection from Conventional AD

**Figure 10:**
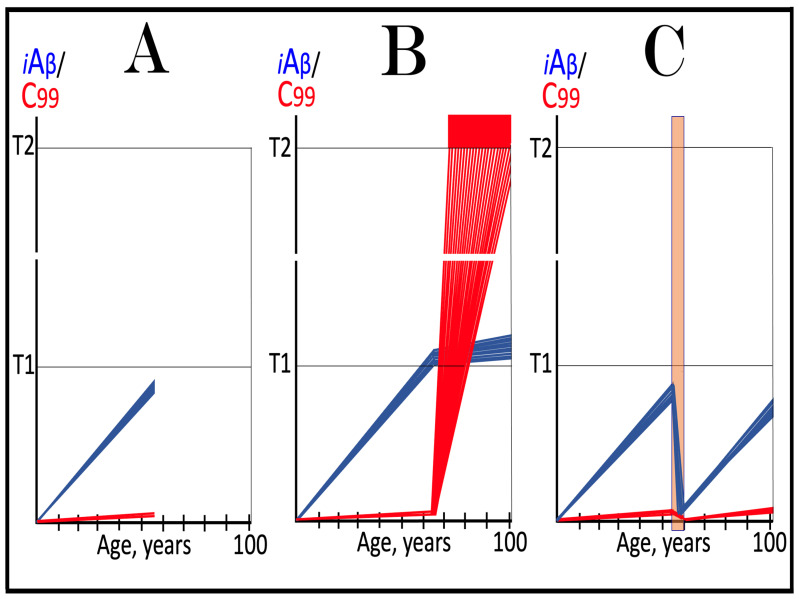
**A single transient targeted iAβ degradation treatment can confer lifelong protection from conventional AD***. Vertical axis*: Relative levels of iAβ and C99. *Horizontal axis*: Age, in years. *Blue lines*: Dynamics of iAβ. *Red lines*: Dynamics of C99. **T1**: Threshold of intracellular concentration of iAβ or C99 that mediates elicitation of the neuronal integrated stress response, which, in turn, enables operation of the AβPP-independent C99 generation pathway and thus triggers the disease. **T2**: Threshold of intracellular concentration of C99 that triggers neuronal death. *Red box*: Range of intracellular concentrations of C99, dubbed “Apoptotic Zone”, associated with apoptosis or necroptosis of neuronal cells. *Orange box*: Duration of the transient iAβ degradation treatment. Panel (**A**): The initial state of levels of iAβ and C99 at the commencement of the transient targeted iAβ degradation treatment. iAβ has not yet reached the T1 threshold. Neither the neuronal ISR has been elicited nor the AβPP-independent C99 production pathway activated. Panel (**B**): Evolution of the initial states of iAβ and C99 in the absence of the ISR suppression treatment. iAβ crosses the T1 threshold. PKR and/or HRI are activated, eIF2α is phosphorylated, and the neuronal IRS is elicited. The production of iAβ in the AβPP proteolytic pathway and its accumulation are suppressed. Concurrently, under ISR conditions, the AβPP-independent C99 generation pathway is activated, and AD commences and progresses. Panel (**C**): Evolution of the initial states of iAβ and C99 under and following the transient targeted iAβ degradation treatment. Under the treatment, iAβ and C99 are depleted. Following the treatment, de novo accumulation of both resumes from low baselines at the pre-treatment rates. Neither would reach the T1 threshold, and no conventional AD would occur within the lifetime of the treated individual.

In the ACH2.0 paradigm, the targeted degradation of iAβ prior to the T1 crossing can potentially have a dramatic and spectacular outcome: a single transient treatment at midlife could be sufficient to provide complete protection from conventional AD and AACD for the remaining lifespan. The rationale for this notion is as follows. Conventional AD is triggered when iAβ crosses the T1 threshold. However, the process of accumulation of iAβ occurs at an exceedingly slow rate. It is so slow that in most individuals, the levels of iAβ do not reach the T1 threshold, and the disease does not occur within their lifetimes. In those individuals who develop late-onset AD, iAβ crosses the T1 threshold not before their mid-sixties. With this in mind, please consider outcomes of a transient but efficient degradation of iAβ implemented at midlife, say in the fifties. Following the treatment, the iAβ population would collapse. Its cellular concentration would be reduced close to basal levels. Its accumulation would resume but from low, nearly basal, levels. It would require decades to reach the T1 threshold, and it could be safely assumed that neither the T1 crossing would occur nor would conventional AD commence within the remaining lifetime of the treated individual. Such an outcome of a single transient iAβ degradation treatment is depicted schematically in Figure 10.

Panel A of Figure 10 depicts the initial state of levels of iAβ and C99 at the commencement of the transient targeted iAβ degradation treatment. Both are below the T1 threshold. Neither the neuronal ISR has been elicited, nor the AβPP-independent C99 production pathway has been activated. Panel B of Figure 10 shows the evolution of the initial state in the untreated individual. iAβ further accumulates and crosses the T1 threshold. As a result, PKR and/or HRI are activated, eIF2α is phosphorylated, and the neuronal IRS is elicited. The production of iAβ in the AβPP proteolytic pathway and its accumulation are suppressed under ISR conditions. Simultaneously, the neuronal ISR induces production of components essential for operation of the AβPP-independent C99 generation pathway. The pathway is activated, C99 rapidly accumulates, and the disease progresses. Panel C of Figure 10 illustrates the evolution of the initial state during and following the targeted transient iAβ degradation treatment. In the presence of the drug, both iAβ and C99 are depleted. When the drug is withdrawn, accumulation of both iAβ and C99 resumes from low baselines and proceeds at the pre-treatment rates. Neither iAβ nor C99 would reach the T1 threshold within the lifetime of the treated individual, and no conventional AD would occur.

## 11. Targeted Degradation of iAβ and/or Aβ Segment of C99 in the Prevention and Treatment of AACD

The logic of the preceding sub-section also applies to aging-associated cognitive decline, and not only to its prevention but also to its cure. This notion is rationalized and illustrated in the following two sub-sections.

### 11.1. A Single Transient iAβ Degradation Treatment Can Prevent AACD for Life

In the ACH2.0 paradigm, AACD is defined as a condition caused and driven by iAβ accumulated above the T^0^ threshold, which morphs into AD upon the T1 crossing. It follows that if the T^0^ crossing by iAβ is prevented, so too would AACD. To prevent the T^0^ crossing, a single transient iAβ degradation treatment can be implemented when the levels of iAβ are still below the T^0^ threshold. As a result of the treatment, the neuronal population of iAβ would collapse. Following the treatment, iAβ accumulation would resume, but from a low, nearly basal baseline. Its levels would not reach the T^0^ threshold, and AACD would not occur within the lifetime of the treated individual. Thus, a single transient iAβ degradation treatment can provide lifelong protection from AACD (and from conventional AD).

**Figure 11:**
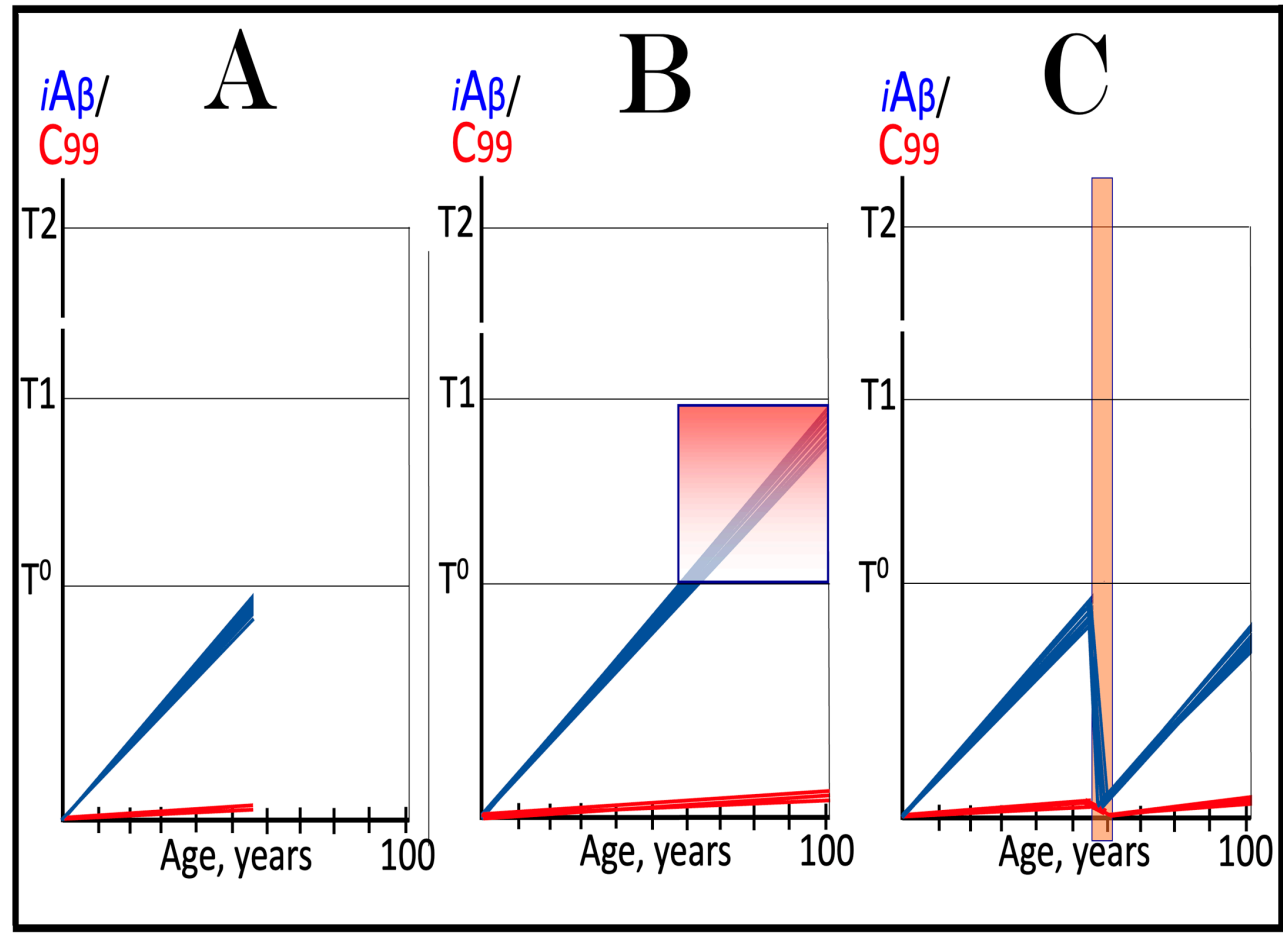
**A single transient iAβ degradation treatment can prevent AACD for life***. Vertical axis*: Relative levels of iAβ and C99. *Horizontal axis*: Age, in years. *Blue lines*: Dynamics of iAβ. *Red lines*: Dynamics of C99. **T^0^**: Threshold of cellular concentration of AβPP-derived *i*Aβ, which triggers neurodegeneration manifesting as AACD. **T1**: Threshold of intracellular concentration of iAβ or C99 that mediates elicitation of the neuronal integrated stress response, which, in turn, enables operation of the AβPP-independent C99 generation pathway and thus triggers the disease. **T2**: Threshold of intracellular concentration of C99 that triggers neuronal death. *Pink box*: The duration and extent of AACD; it initiates when iAβ crosses the T^0^ threshold and morphs into AD when the T1 threshold is crossed, unless iAβ does not reach the T1 threshold, in which case AACD persists for the remaining portion of the lifetime. *Orange box*: Duration of the transient iAβ degradation treatment. Panel (**A**): The initial state of levels of iAβ and C99 at the commencement of the transient targeted iAβ degradation treatment. iAβ has not yet reached the T^0^ threshold. Panel (**B**): Evolution of the initial states of iAβ and C99 in the absence of the iAβ degradation treatment. iAβ crosses the T^0^ threshold. AACD commences and persists for the remaining lifetime of the individual. Panel (**C**): Evolution of the initial states of iAβ and C99 under and following the transient targeted iAβ degradation treatment. Under the treatment levels, iAβ and C99 are depleted. Following the treatment, de novo accumulation of both resumes from low baselines at the pre-treatment rates. iAβ would not reach the T^0^ threshold, and no AACD would occur within the lifetime of the treated individual.

The above scenario is illustrated in Figure 11. Panel A of Figure 11 depicts the initial state of iAβ levels at the commencement of the treatment. They are below the T^0^ threshold. Panel B of Figure 11 shows the evolution of the initial state in the untreated individual. iAβ accumulates unabated. When it crosses the T^0^ threshold, AACD commences and persists for the remaining lifetime of the individual. Panel C of Figure 11 details outcomes of the transient iAβ-degrading treatment. Following the treatment, levels of iAβ are drastically reduced. Its accumulation resumes, but from a low baseline. It would not cross the T^0^ threshold, and AACD would not occur within the lifetime of the treated individual. Thus, a single transient iAβ degradation treatment confers protection from AACD and from conventional AD lasting the lifetime.

### 11.2. A Single Transient iAβ Degradation Treatment Can Cure AACD and Prevent Its Recurrence for Life

As follows from the preceding sections, for the purposes of prevention, a single transient iAβ degradation treatment is equally efficient for AACD and conventional AD: It provides lifelong protection from both conditions. This is not the case, however, when the cure or treatment of both conditions is concerned. As discussed above, a reduction in the levels of iAβ, including its degradation, would be absolutely futile in the neurons where the crossing of the T1 threshold has already occurred. This is because in these neurons, the integrated stress response has already been elicited and the AβPP-independent C99 production pathway activated. This, in fact, is the pathway that drives AD pathology, and it is autonomous and self-sufficient in that its product, C99, sustains the ISR state and thus propagates and perpetuates its own generation. Therefore, a reduction in iAβ, even its complete removal, would have no effect whatsoever on the operation of the AβPP-independent C99 generation pathway and on the progression of AD.

**Figure 12:**
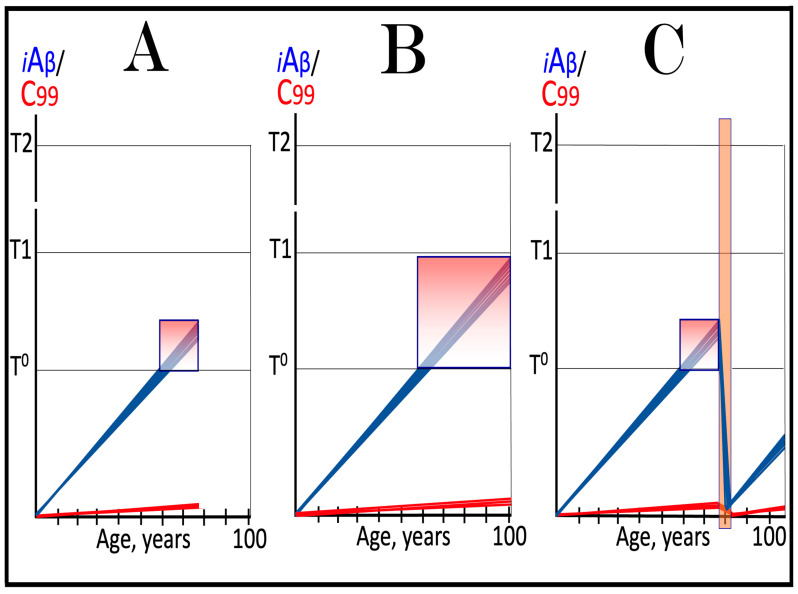
**A single transient iAβ degradation treatment can cure AACD and prevent its recurrence for life***. Vertical axis*: Relative levels of iAβ and C99. *Horizontal axis*: Age, in years. *Blue lines*: Dynamics of iAβ. *Red lines*: Dynamics of C99. **T^0^**: Threshold of cellular concentration of AβPP-derived *i*Aβ, which triggers neurodegeneration manifesting as AACD. **T1**: Threshold of intracellular concentration of iAβ or C99 that mediates elicitation of the neuronal integrated stress response, which, in turn, enables operation of the AβPP-independent C99 generation pathway and thus triggers the disease. **T2**: Threshold of intracellular concentration of C99 that triggers neuronal death. *Pink boxes*: The duration and extent of AACD; it initiates when iAβ crosses the T^0^ threshold and morphs into AD when the T1 threshold is crossed, unless iAβ does not reach the T1 threshold, in which case AACD persists for the remaining portion of the lifetime. *Orange boxes*: Duration of the transient iAβ degradation treatment. Panel (**A**): The initial state of levels of iAβ and C99 at the commencement of the transient targeted iAβ degradation treatment. iAβ has reached and crossed the T^0^ threshold. AACD has commenced and progressed. Panel (**B**): Evolution of the initial states of iAβ and C99 in the absence of the iAβ degradation treatment. iAβ continues to accumulate, and AACD progresses and, in the absence of the T1 crossing, persists for the remaining lifetime of the individual. Panel (**C**): Evolution of the initial states of iAβ and C99 under and following the transient targeted iAβ degradation treatment. Under the treatment iAβ and C99 are depleted, and the condition is cured. Following the treatment, de novo accumulation of both resumes from low baselines at the pre-treatment rates. iAβ would not reach the T^0^ threshold, and no AACD would recur within the lifetime of the treated individual.

In AACD, the situation is markedly different. This condition is caused and driven by iAβ in the range of concentrations between the T^0^ and T1 thresholds (it morphs into AD upon the T1 crossing). In this range, the AβPP-independent C99 production pathway is inoperative, and the reduction in iAβ levels below the T^0^ threshold would remove the driver of AD and cure the condition. This scenario is illustrated in Figure 12. Panel A of Figure 12 depicts the initial state of levels of iAβ at the start of the iAβ degradation treatment. They have crossed the T^0^ threshold in all affected neurons, and AACD has commenced. Panel B of Figure 12 shows the evolution of the initial state in the absence of the treatment. Accumulation of iAβ continues unimpeded, and AACD persists for the remaining lifespan of the individual. Panel C of Figure 12 details the outcome of the transient iAβ degradation treatment. As the levels of iAβ are decreasing, they reverse-cross the T^0^ threshold. With its driver removed, the condition is cured. Cellular concentration of iAβ declines further, to nearly the basal level. Following the transient treatment, accumulation of iAβ resumes, but from a low baseline. It would not re-cross the T^0^ threshold, and AACD would not recur for the remaining lifespan of the treated individual. Thus, a single transient iAβ degradation treatment would cure AACD, protect from its recurrence, and prevent conventional AD for life.

## 12. A Single Transient iAβ/C99 Degradation Therapy Can Effectively Treat Conventional AD Only in the Presence of ISR Inhibitors

### 12.1. iAβ/C99 Degradation Therapy Is Ineffective When the AβPP-Independent C99 Production Pathway Is Operational

As discussed above, in the ACH2.0 paradigm AD pathology is driven by C99 generated in the AβPP-independent pathway. It follows that in order to successfully treat the disease, we must reduce levels of C99 to those below their AD pathology-causing range. Arguably, this appears easy to do. The active, pathology-driving portion of C99 is, apparently, its N-terminal Aβ segment. Indeed, the removal of this segment by gamma-cleavage leaves non-toxic AβPP intracellular domain, and so does the removal of only sixteen N-terminal amino acid residues of C99 by the alpha-cleavage, which leaves non-toxic C83. Therefore, there is every reason to expect that degradation of the iAβ and of the Aβ segment of C99 would be highly effective in the treatment of conventional AD. In fact, the outcome of such therapy would be conceptually similar to that of the preventive iAβ degradation treatment discussed above. Indeed, targeted degradation of the Aβ segment of C99 would remove the driver of AD pathology, and progression of the disease would cease. Moreover, when levels of iAβ are reduced to those below the T1 threshold, the neuronal ISR state would reverse, and the AβPP-independent C99 production pathway would be disabled. Eventually, levels of iAβ and of C99 would be reduced to basal. Following the transient treatment, accumulation of both would resume from low baselines, and neither would cross the T1 threshold nor would conventional AD recur in the treated individuals.

However, the possibility that targeted degradation of iAβ and of the Aβ segment of C99 would, on its own, yield such an outcome is highly unlikely. The reason for this is the extraordinarily high rate of influx of C99 produced in the AβPP-independent pathway. Indeed, as discussed below, C99 generated independently of AβPP is apparently the major translation product in AD-affected neurons. This is in sharp contrast to the preventive application of the iAβ degradation treatment, where the influx of iAβ is very slow and its accumulation can be easily reversed. As an example, the Icelandic Aβ mutation decreases the rate of production of Aβ by only about 30%, yet this is sufficient to confer protection from the occurrence of conventional AD and AACD. To successfully treat AD, the accumulation of C99 must be reversed, but its rate of influx is apparently such that its accumulation can only be slowed down. The result of such a treatment would be slowing down the progression of AD pathology for the duration of the treatment, but the disease would persist.

**Figure 13:**
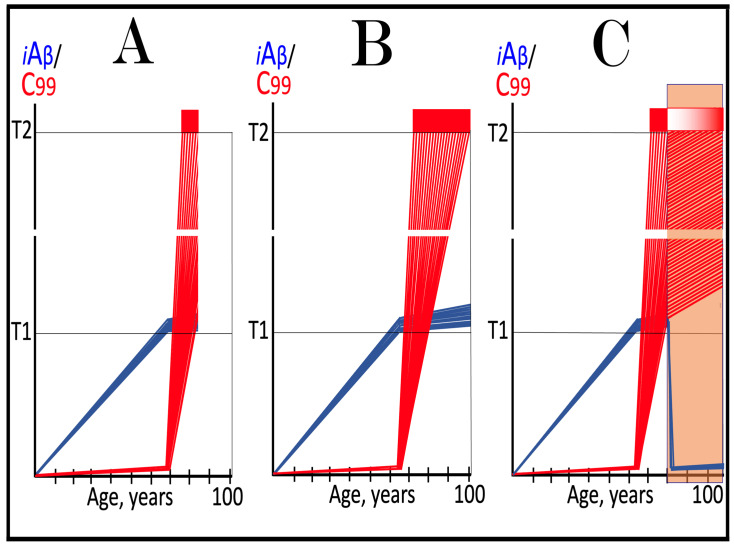
**iAβ/C99 degradation therapy would be ineffective on its own when the AβPP-independent C99 production pathway is operational**. *Vertical axis*: Relative levels of iAβ and C99. *Horizontal axis*: Age, in years. *Blue lines*: Dynamics of iAβ. *Red lines*: Dynamics of C99. **T1**: Threshold of intracellular concentration of iAβ or C99 that mediates elicitation of the neuronal integrated stress response, which, in turn, enables operation of the AβPP-independent C99 generation pathway and thus triggers the disease. **T2**: Threshold of intracellular concentration of C99 that triggers neuronal death. *Red box*: Range of intracellular concentrations of C99, dubbed the “Apoptotic Zone”, associated with apoptosis or necroptosis of neuronal cells. *Orange box*: Duration of the targeted iAβ/C99 degradation treatment. Panel (**A**): The initial state of levels of iAβ and C99 in the affected neurons at the commencement of the iAβ/C99 degradation treatment. AβPP-derived iAβ has crossed the T1 threshold. The neuronal ISR has been elicited, and the production and accumulation of iAβ have been suppressed. Concurrently, the neuronal ISR provided components essential for the AβPP-independent C99 generation pathway and enabled its operation. Levels of C99 have rapidly increased. They crossed the T2 threshold in a fraction of the affected neurons, and AD symptoms have manifested. Panel (**B**): Evolution of the initial states of iAβ and C99 in the untreated AD patient. The accumulation of C99 and progression of the disease proceed unimpeded; the disease reaches its end stage. Panel (**C**): Evolution of the initial states of iAβ and C99 under the iAβ/C99 degradation treatment. iAβ is rapidly depleted, and its levels remain low for the duration of the treatment. On the other hand, the influx of C99 produced in the AβPP-independent pathway is such that its accumulation is only reduced but not reversed. The progression of the disease continues, albeit at a reduced rate.

The above scenario is illustrated in Figure 13. Panel A of Figure 13 depicts the initial state of the levels of iAβ and C99 at the start of the degradation treatment targeting iAβ and the Aβ segment of C99. At this stage, AβPP-derived iAβ has accumulated over the T1 threshold and triggered the elicitation of the neuronal integrated stress response. As a result, production and accumulation of iAβ have been suppressed. Concurrently, the neuronal ISR state provided the essential components of and activated the AβPP-independent C99 generation pathway. C99 has rapidly accumulated and crossed the T1 threshold. Its accumulation has continued; in a fraction of the neurons, it has crossed the apoptotic T2 threshold, and AD symptoms have manifested. Panel B of Figure 13 shows the evolution of the initial state in the absence of the treatment. C99 continues its accumulation unimpeded. It crosses the T2 threshold in more neurons, and the disease enters its end stage. Panel C of Figure 13 details the outcome of the degradation treatment targeting iAβ and the Aβ segment of C99. Due to the decreased rate of influx of iAβ, its accumulation is reversed, and its levels rapidly decline. On the other hand, due to its high rate of influx, the accumulation of C99 only slows down. The rate of progression of AD pathology declines for the duration of the treatment, but the disease persists.

### 12.2. Composite Transient Therapy Comprising Targeted iAβ/C99 Degradation and ISR Inhibition Can Effectively Treat Conventional AD

It follows from the preceding section that the degradation treatment targeting iAβ and the Aβ segment of C99 can potentially slow down the progression of AD but cannot stop it. The underlying problem is clear: A high rate of influx of C99 produced in the AβPP-independent pathway. The solution for this problem is equally clear: Suppress or stop the influx of C99 generated independently of AβPP for the duration of the degradation treatment. As discussed above, this is perfectly doable, and the means to accomplish it is the inhibition of the neuronal integrated stress response. Indeed, with the neuronal ISR suppressed, the supply of the components essential for the operation of the AβPP-independent C99 generation pathway would cease, the pathway would be disabled and the influx of C99 would terminate. In such a setting, degradation of the Aβ segment of C99 would be very effective in rapidly depleting cellular levels of C99 below the T1 threshold and thus stopping both the operation of the AβPP-independent C99 generation pathway and progression of AD pathology. For the reasons discussed above, systemic ISR inhibitors can be used only transiently, but even their transient implementation would be sufficient to allow a substantial reduction in both iAβ and C99 by concurrently deployed Aβ-degrading agents. Following such a composite transient iAβ/C99 depletion therapy, de novo accumulation of iAβ and C99 would resume, but from low baselines. iAβ would not re-cross the T1 threshold, the neuronal ISR would not be re-elicited, the AβPP-independent C99 production pathway would not be re-activated, and conventional AD would not recur for the remaining lifespan of the treated individual.

**Figure 14:**
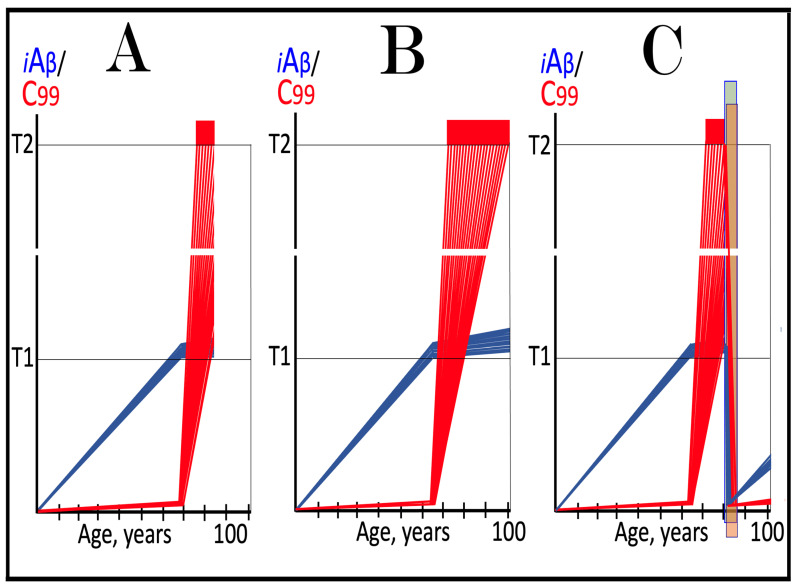
**Composite transient therapy comprising targeted iAβ/C99 degradation and ISR inhibition can effectively treat conventional AD**. *Vertical axis*: Relative levels of iAβ and C99. *Horizontal axis*: Age, in years. *Blue lines*: Dynamics of iAβ. *Red lines*: Dynamics of C99. **T1**: Threshold of intracellular concentration of iAβ or C99 that mediates elicitation of the neuronal integrated stress response, which, in turn, enables operation of the AβPP-independent C99 generation pathway and thus triggers the disease. **T2**: Threshold of intracellular concentration of C99 that triggers neuronal death. *Red box*: Range of intracellular concentrations of C99, dubbed the “Apoptotic Zone”, associated with apoptosis or necroptosis of neuronal cells. *Green box*: Duration of the ISR suppression treatment. *Orange box*: Duration of the targeted iAβ/C99 degradation treatment. Panel (**A**): The initial state of levels of iAβ and C99 in the affected neurons at the commencement of the iAβ/C99 degradation treatment. AβPP-derived iAβ has crossed the T1 threshold. The neuronal ISR has been elicited, and the production and accumulation of iAβ have been suppressed. Concurrently, the neuronal ISR provided components essential for the AβPP-independent C99 generation pathway and enabled its operation. Levels of C99 have rapidly increased. They crossed the T2 threshold in a fraction of the affected neurons, and AD symptoms have manifested. Panel (**B**): Evolution of the initial states of iAβ and C99 in the untreated AD patient. The accumulation of C99 and progression of the disease proceed unimpeded; the disease reaches its end stage. Panel (**C**): Evolution of the initial states of iAβ and C99 under and following the composite iAβ/C99 degradation/ISR inhibition treatment. During the transient treatment, the neuronal ISR state is reversed. With the supply of the essential components discontinued, the AβPP-independent C99 generation pathway is disabled and the influx of C99 produced in this pathway ceases. With no influx of C99 produced independently of AβPP, the implementation of the iAβ/C99 degradation therapy rapidly depletes both to nearly basal levels. Without its C99 driver, the progression of AD pathology stops. Following the treatment, iAβ and C99 resume their accumulation low baselines. They do not reach the T1 threshold, and conventional AD does not recur for the remaining lifetime of the individual.

The above scenario is illustrated in Figure 14. Panel A of Figure 14 shows the initial state of the levels of iAβ and C99 at the commencement of the treatment. It is the same as in the preceding Figure. The T1 threshold has been crossed, the neuronal ISR elicited, accumulation of iAβ suppressed, and the AβPP-independent C99 production pathway activated. In a fraction of the neurons, the T2 threshold has been crossed, and AD symptoms have manifested. Panel B of Figure 14 depicts the evolution of the initial state in the untreated individual. It is again identical to that shown in the preceding Figure. C99 continues to rapidly accumulate, more neurons cross the T2 threshold, and the disease progresses unimpeded. On the other hand, the outcome of the transient composite treatment comprising ISR inhibition and iAβ/C99 degradation (Panel C of Figure 14) is strikingly different from a single iAβ/C99 degradation therapy shown in the preceding Figure. Under ISR inhibitors, the neuronal ISR state is reversed. With the supply of the essential components discontinued, the AβPP-independent C99 generation pathway is disabled and the influx of C99 produced in this pathway ceases. With no influx of C99 produced independently of AβPP, the implementation of the iAβ/C99 degradation therapy would rapidly deplete both to nearly basal levels. The progression of AD pathology would stop, and iAβ would resume its accumulation from a low baseline. It would not reach the T1 threshold, and conventional AD would not recur for the remaining lifetime of the individual.

## 13. Activators of Intra-iAβ Cleaving Capabilities of BACE1 and BACE2 as Potential Drugs for the Prevention and Treatment of Conventional AD and AACD

The preceding sections conclude that agents capable of effective degradation of iAβ and of the Aβ segment of C99 could potentially prevent conventional AD, prevent and cure AACD, and, in combination with ISR inhibitors, treat conventional AD. In other words, such agents are potent AD and AACD drugs. The question is, what are they? iAβ -degrading agents can certainly be designed and manufactured, for example, as proteolysis-targeting chimeras (PROTACs) or molecular-glue degraders (MGDs). Alternatively, we can adopt naturally occurring agents, which perform targeted degradation of iAβ and of the Aβ segment of C99 as their physiological function. Such agents do, in fact, occur physiologically, and they are BACE1 and BACE2, or, rather, the intra-iAβ cleaving activities of both enzymes.

Indeed, as discussed above, both BACE 1 and BACE2 are capable of cleaving within iAβ and the Aβ segments of AβPP and C99. BACE1-enacted cleavages occur at the β’ site, ten amino acids downstream of the N-terminus of Aβ. BACE1 also cleaves at positions Aβ34 and Aβ35 [219,220,221,222]; this generates intermediates in the iAβ clearing process. Potential benefits of BACE1-mediated intra-Aβ cleavages have been convincingly indicated by the outcomes of the Icelandic Aβ mutation, which increases the efficiency of cleavages at the β’ site and protects from conventional AD, and demonstrated in transgenic mouse models and cultured neuronal cells overexpressing BACE1 [219,220,221,222,223,224,225,226,227].

While intra-Aβ cleavages are a minor, secondary activity of BACE1, they are the major, principal physiological activity of BACE2. Indeed, BACE2 cleaves at positions Aβ19 and Aβ20 [227,228]. The therapeutic potential of BACE2 intra-Aβ cleavage activity has been lucidly implied by the outcomes of the Flemish Aβ mutation, which suppresses the intra-Aβ cleaving activity of BACE2 and causes early onset of AD [179,180,181,182,183,184,185,186]. It was also demonstrated in transgenic mouse models, where production and deposition of Aβ were significantly elevated when BACE2 activity was suppressed [228]. Moreover, an increase in the BACE2 activity protected from neuronal death, whereas its suppression was associated with elevated neurodegeneration in human pluripotent cell-derived brain organoids [229].

It follows that activation of the intra-Aβ cleaving capabilities of BACE1 and BACE2 is a plausible therapeutic strategy for conventional AD. Accordingly, enhancers of inta-Aβ-cleaving activities of BACE1 and BACE2 potentially constitute potent AD drugs. Activation of either BACE1 or BACE2 would be effective in the prevention and treatment of conventional AD and AACD. Moreover, the concurrent activation of both may have powerful synergistic effects. This is because not only do these enzymes have different intra-Aβ target sites, but they are also localized in distinct cellular compartments [230]. The bottom line is that activators of the intra-Aβ cleaving capabilities of BACE1 and BACE2 are, apparently, ideal drugs for conventional AD and AACD.

## 14. Therapeutic Options for the Prevention and Treatment of Unconventional AD: A Brief Overview

Therapeutic options described above for conventional AD, or at least some of them, are also applicable to the unconventional form of the disease. For two reasons, their effects and their anticipated outcomes would, however, be different in unconventional AD. One reason is that unconventional AD commences at any level of iAβ below the T1 threshold (if iAβ were to cross the T1 threshold, the disease would commence conventionally). Therefore, any measures precluding iAβ from reaching the T1 threshold would be completely ineffective in the unconventional form of the disease. Another reason is the persistence of the unconventionally elicited neuronal integrated stress response. Unconventional AD is always triggered by the neuronal ISR elicited unconventionally and sustained at least until C99 produced independently of AβPP crosses the T1 threshold. Following the T1 crossing, if the unconventional stressor is withdrawn, the neuronal ISR state would be sustained by C99, and unconventional AD would be converted to its conventional form. In such a case, therapeutic options outlined in the preceding section would fully apply (see the following section). Therefore, the following sections consider only a scenario where an unconventional ISR-eliciting stressor, once it appears, persists for the duration of the individual’s lifespan. In such cases, some options that are effective in conventional AD would become ineffective and would require certain modifications in order to retain their relevance for the unconventional form of the disease.

## 15. Sustained Removal of Unconventional Stressors or Sufficient Reduction in Their Levels Would Reverse the Neuronal ISR and Convert Unconventional AD into Conventional One, Preventable and Treatable by a Single Transient Composite iAβ and C99 Degradation/ISR Inhibition Therapy

The best therapeutic options for conventional AD, outlined above, namely, once-in-a-lifetime transient iAβ degradation treatment for the prevention of the disease and once-only transient composite iAβ degradation/ISR suppression treatment for its treatment, are ineffective in the unconventional form of the disease. This is due to the persistent presence of unconventional ISR-eliciting stressors. Indeed, transient implementation of the Aβ degradation treatment, in conjunction with the ISR suppression, would disable the AβPP-independent C99 production pathway and reduce levels of C99 and iAβ to basal. But as soon as the drugs are withdrawn, the neuronal ISR would be unconventionally re-activated due to the persistent presence of unconventional stressors, the AβPP-independent C99 production pathway would be re-initiated, and rapid accumulation of C99 would resume. The treatment, thus, would provide recourse only for the duration of its transient implementation.

The optimal therapeutic strategy for unconventional AD, therefore, would be to identify and remove unconventional ISR-eliciting stressors or to deplete their levels to those below the ISR-eliciting threshold, and thus to convert the disease into its conventional form. With this accomplished, a single transient iAβ/C99 degradation treatment would be rendered perfectly effective in the prevention of the disease, and a single transient composite iAβ and C99 degradation/ISR suppression treatment would be equally effective in its treatment.

**Figure 15:**
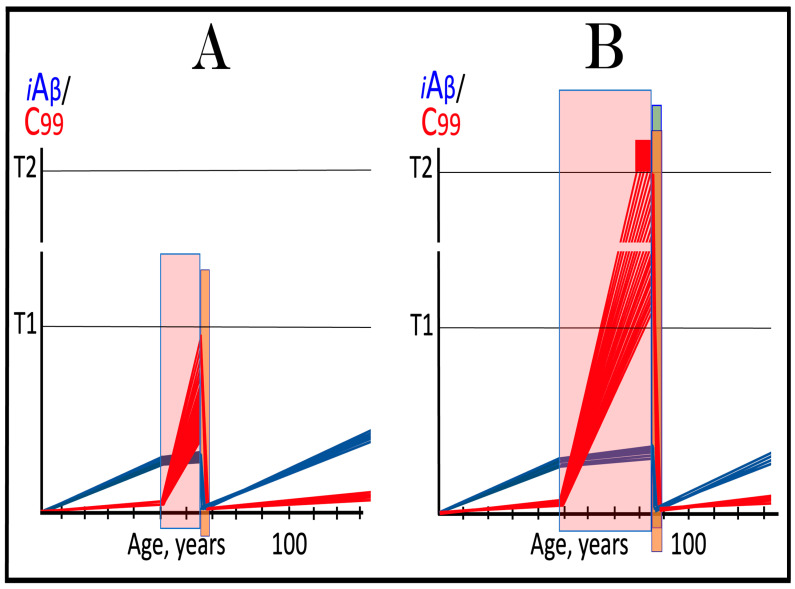
**Sustained removal of unconventional stressors or their depletion below the neuronal ISR-eliciting threshold, followed by targeted degradation of iAβ/C99 as the optimal strategy for the prevention and treatment of unconventional AD**. *Vertical axis*: Relative levels of iAβ and C99. *Horizontal axis*: Age, in years. *Blue lines*: Dynamics of iAβ. *Red lines*: Dynamics of C99. **T1**: Threshold of intracellular concentration of iAβ or C99 that mediates elicitation of the neuronal integrated stress response, which, in turn, enables operation of the AβPP-independent C99 generation pathway and thus triggers the disease. **T2**: Threshold of intracellular concentration of C99 that triggers neuronal death. *Red box*: Range of intracellular concentrations of C99, dubbed the “Apoptotic Zone”, associated with apoptosis or necroptosis of neuronal cells. *Pink box*: Duration of the occurrence of unconventional stressors at concentrations sufficient to elicit the neuronal ISR. *Green box*: Duration of the ISR suppression treatment. *Orange box*: Duration of the targeted iAβ/C99 degradation treatment. Panel (**A**): Sustained removal of unconventional stressors or their depletion below the neuronal ISR-eliciting threshold enables efficient transient iAβ/C99 depletion therapy for the prevention of AD. At the commencement of the treatment, the neuronal ISR has been unconventionally elicited, and production and accumulation of AβPP-derived iAβ have been suppressed. Simultaneously, the AβPP-independent C99 generation pathway has been unconventionally activated. C99 has accumulated but is still below the T1 threshold. The transient administration of the iAβ/C99 degradation therapy is carried out concurrently with the sustained removal of unconventional stressors or their depletion below the neuronal ISR-eliciting levels. With unconventional stressors removed, the ISR state is reversed, the AβPP-independent C99 production pathway is disabled, the influx of C99 produced independently of AβPP ceases, and the transient iAβ/C99 depletion treatment depletes both to nearly basal levels. Following the transient iAβ/C99 degradation treatment, de novo accumulation of both resumes from low baselines and proceeds at pre-ISR elicitation rates. The T1 threshold would not be reached, and the disease would not occur within the lifetime of the treated individual as long as unconventional stressors are maintained below ISR-eliciting levels. Panel (**B**): Sustained removal of unconventional stressors or their depletion below the neuronal ISR-eliciting threshold enables efficient transient composite iAβ/C99 depletion/ISR inhibition therapy for the treatment of AD. At the start of the treatment, the neuronal ISR has been unconventionally elicited, and production and accumulation of AβPP-derived iAβ have been suppressed. Concurrently, the AβPP-independent C99 generation pathway has been unconventionally activated. C99 has accumulated, crossed the T1 threshold, and the disease commenced. When C99 has crossed the T2 threshold in a fraction of the neurons, AD symptoms have manifested. The treatment consists of three parts. The first is the sustained removal of unconventional stressors or their depletion below the neuronal ISR-eliciting levels. The ISR state, however, remains in effect and is sustained by C99 produced in the AβPP-independent pathway. Therefore, the second part is the transient inhibition of the neuronal ISR. This disables the AβPP-independent C99 generation pathway, ceases the influx of C99 produced independently of AβPP, and stops progression of AD. Concurrently, as the third part, transient iAβ/C99 degradation treatment is implemented. This results in depletion of both. Their de novo accumulation resumes from low baselines and proceeds at pre-ISR elicitation rates. The T1 threshold would not be reached, and the disease would not recur within the lifetime of the treated individual as long as unconventional stressors are maintained below neuronal ISR-eliciting levels.

This strategy is illustrated in Figure 15. In Panel A of Figure 15, by the time of the commencement of the treatment (orange box), the neuronal ISR has been elicited by an unconventional stressor (pink box) at low (under-T1) levels of iAβ. Under ISR conditions, the accumulation of iAβ has been suppressed. Contemporaneously, the AβPP-independent C99 production pathway has been activated. Its product (i.e., C99) has rapidly accumulated but has not yet reached the T1 threshold, and the disease has not yet commenced. A composite treatment, comprising sustained removal of the unconventional stressor and transient iAβ/C99 degradation, is implemented at this point. The sustained removal of the unconventional stressor reverses the ISR state and disables the AβPP-independent C99 production pathway. The influx of C99 generated independently of AβPP ceases. Under these conditions, the transient iAβ/C99 degradation treatment effectively depletes both substrates. Their de novo accumulation resumes from low baselines, and neither iAβ nor C99 reaches the T1 threshold within the lifespan of the treated individual. The key to this strategy is the sustained (for life) removal of unconventional stressors. It converts unconventional circumstances into conventional ones.

In panel B of Figure 15, by the time of the initiation of the treatment, the neuronal ISR has been elicited by an unconventional stressor at low, under-T1, levels of iAβ. Under the ISR conditions, accumulation of iAβ has been suppressed, but the production of C99 in an AβPP-independent manner has been activated. C99 has rapidly accumulated, crossed the T1 threshold, and the disease has commenced. C99 has further accumulated, crossed the T2 threshold in a fraction of the neurons, and AD symptoms have manifested. The sustained removal of the unconventional stressor, implemented at this point, converts the disease into a conventional one. It does not, however, reverse the neuronal ISR state because it is sustained by C99 at the over-T1 levels. Therefore, the concurrent transient iAβ/C99 degradation treatment is combined with the transient (for the same duration) suppression of ISR. As a result, the neuronal ISR state is reversed, the AβPP-independent C99 production pathway is disabled, and the influx of C99, generated independently of AβPP, ceases. Under these conditions, C99 and iAβ are rapidly depleted to levels well below the T1 threshold, and the progression of the disease abates. The de novo accumulation of both iAβ and C99 resumes from low baselines and is supported solely by the AβPP proteolysis. Neither would reach the T1 threshold, and the disease would not recur within the remaining lifespan of the treated patient. As in the prevention scenario discussed above, the key to this strategy is the conversion of unconventional AD into the conventional form of the disease.

## 16. Long-Term Targeted Degradation of iAβ and of the Aβ Segment of C99 in the Prevention and Treatment of Unconventional AD

The present section considers the effects of the long-term iAβ/C99 degradation treatment in the prevention and treatment of unconventional AD. The usefulness of this approach is limited by its two attributes. One is its inefficiency. Indeed, as discussed above, the rate of C99 production in the AβPP-independent pathway is apparently significantly greater than the rate of its degradation. Consequently, in any iAβ/C99 degradation therapy, the influx of C99 always exceeds its efflux. Its accumulation would persist under the treatment, and it would continue to sustain the neuronal ISR state and the activity of the AβPP-independent pathway of its own production. Therefore, with the neuronal ISR unconventionally elicited and the AβPP-independent C99 generation pathway activated, the occurrence of the disease would only be delayed but not prevented, and, once the disease commences, its progression would only be slowed down but not stopped.

Another feature limiting the usefulness of the long-term iAβ/C99 degradation therapy is the inapplicability of activators of BACE1 and BACE2. As discussed above, physiologically occurring intra-Aβ cleavage activities of BACE 1 and BACE2 constitute proverbial “presents” offered by nature, and their activators constitute highly promising potential AD drugs. Such drugs, however, are inapplicable on their own in the unconventional form of AD. This is because, as soon as the neuronal ISR is elicited, the production of both BACE1 and BACE2 is suppressed along with that of the bulk of cellular proteins. Accordingly, the present section and the following Section 17 consider only iAβ-degrading agents other than BACE1 and BACE2. It should be mentioned in this context that, in contrast with BACE1 and BACE2, the generation of C99 in the AβPP-independent pathway and production of tau protein in AD are independent from the neuronal ISR state: The former is translated from modified mRNA (see Section 22 below) and the latter from an IRES element in the 5′UTR of tau mRNA [153].

### 16.1. Long-Term Targeted Degradation of iAβ and of the Aβ Segment of C99 in the Prevention of Unconventional AD

**Figure 16:**
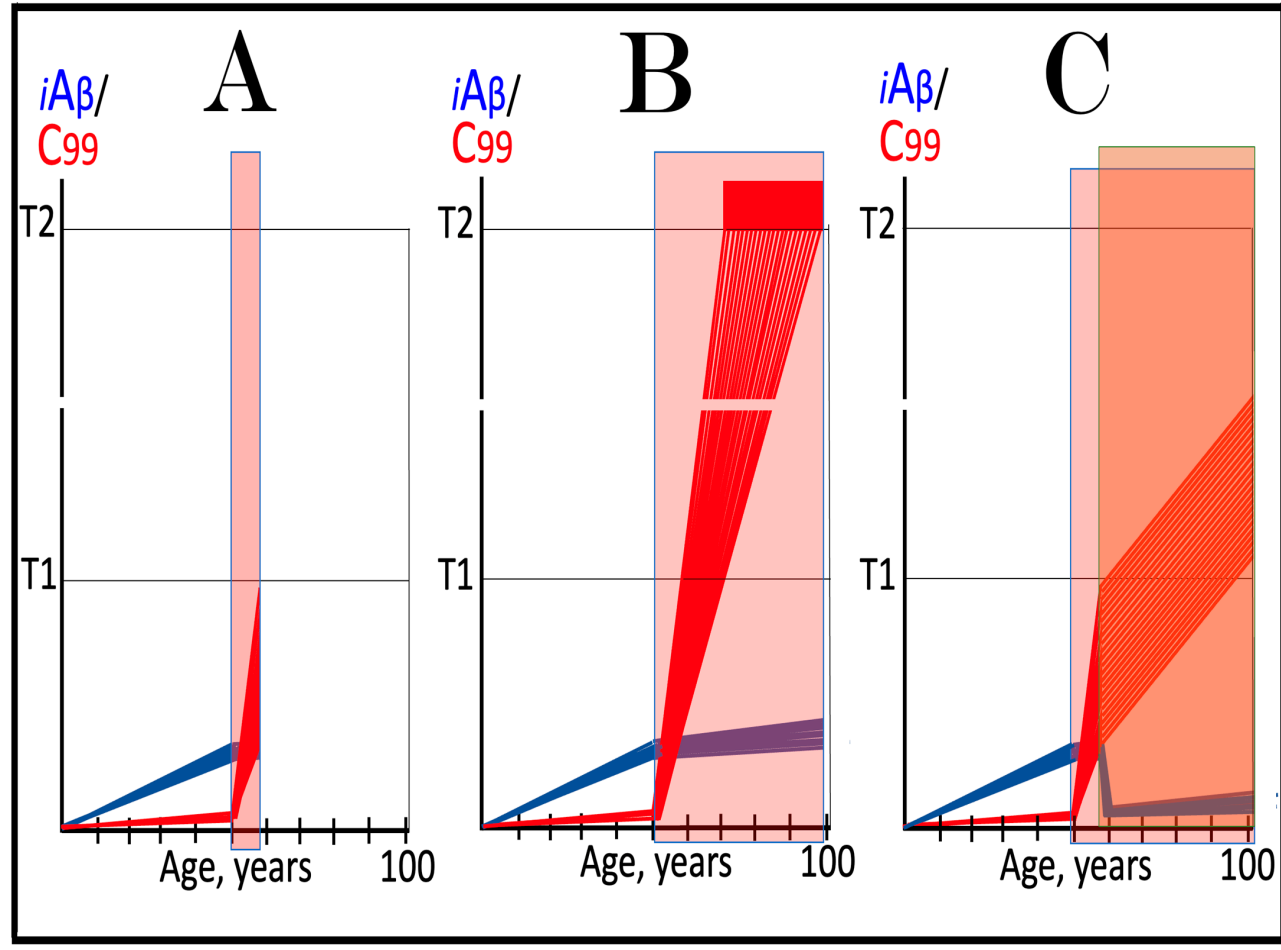
**Long-term targeted degradation of iAβ and C99 in the prevention of unconventional AD**. *Vertical axis*: Relative levels of iAβ and C99. *Horizontal axis*: Age, in years. *Blue lines*: Dynamics of iAβ. *Red lines*: Dynamics of C99. **T1**: Threshold of intracellular concentration of iAβ or C99 that mediates elicitation of the neuronal integrated stress response, which, in turn, enables operation of the AβPP-independent C99 generation pathway and thus triggers the disease. **T2**: Threshold of intracellular concentration of C99 that triggers neuronal death. *Red box*: Range of intracellular concentrations of C99, dubbed the “Apoptotic Zone”, associated with apoptosis or necroptosis of neuronal cells. *Pink boxes*: Duration of the occurrence of unconventional stressors at concentrations sufficient to elicit the neuronal ISR. *Orange box*: Duration of the targeted iAβ/C99 degradation treatment. Panel (**A**): The initial state of levels of iAβ and C99 at the commencement of the treatment. The neuronal ISR has been unconventionally elicited, and production and accumulation of AβPP-derived iAβ have been suppressed. Concurrently, under ISR conditions, the AβPP-independent C99 generation pathway has been unconventionally activated. C99 produced independently of AβPP has rapidly accumulated but is still below the T1 threshold. Panel (**B**): Evolution of the initial state in the absence of the treatment. C99 crosses the T1 threshold and the AβPP-independent C99 production pathway is rendered self-sustained. AD commences and progresses until the end stage is reached. Panel (**C**): Evolution of the initial state under the long-term iAβ/C99 degradation treatment. iAβ is rapidly depleted, and its levels remain low for the duration of the treatment. The rate of C99 degradation cannot match that of its production in the AβPP-independent pathway. Its accumulation continues, but at a lower rate. It crosses the T1 threshold, and the AβPP-independent C99 production pathway becomes autonomous. The disease commences, but its progression occurs at a reduced rate for the duration of the treatment. Thus, under the treatment, the disease is not prevented but its occurrence is delayed and the rate of its progression is reduced.

Effects of the long-term targeted degradation of iAβ and of the Aβ segment of C99 are illustrated in Figure 16. Panel A of Figure 16 depicts the initial state of levels of iAβ and C99 at the start of the treatment. At this point, the neuronal ISR has already been elicited by an unconventional stressor. Production and accumulation of iAβ have been suppressed, but generation of C99 in the AβPP-independent pathway and its rapid accumulation have been activated. C99 levels have rapidly increased but are still below the T1 threshold; no AD has yet commenced. Panel B of Figure 16 shows the evolution of the initial state in the absence of treatment. Accumulation of C99 produced in the AβPP-independent pathway continues unabated. When its levels cross the T1 threshold, the disease commences and progresses until it reaches its end stage.

Panel C of Figure 16 details the evolution of the initial state in the presence of agents specifically targeting iAβ and the Aβ segment of C99 (in all likelihood, an agent specifically degrading iAβ would also be able to cleave within the Aβ segment of C99). As a result, iAβ, produced only in the AβPP proteolytic pathway, is depleted, and its accumulation, already suppressed under ISR conditions, is reversed. Concurrently, the efflux of C99 is increased, but its rate is still less than that of its influx in the AβPP-independent pathway. Consequently, under the treatment, C99 keeps accumulating, albeit at a reduced rate. Its levels would cross the T1 threshold, but the crossing would be delayed. Upon T1 crossing, the disease would commence, but its progression would be slowed down for the duration of the treatment.

### 16.2. Long-Term Targeted Degradation of iAβ and of the Aβ Segment of C99 in the Treatment of Unconventional AD

**Figure 17:**
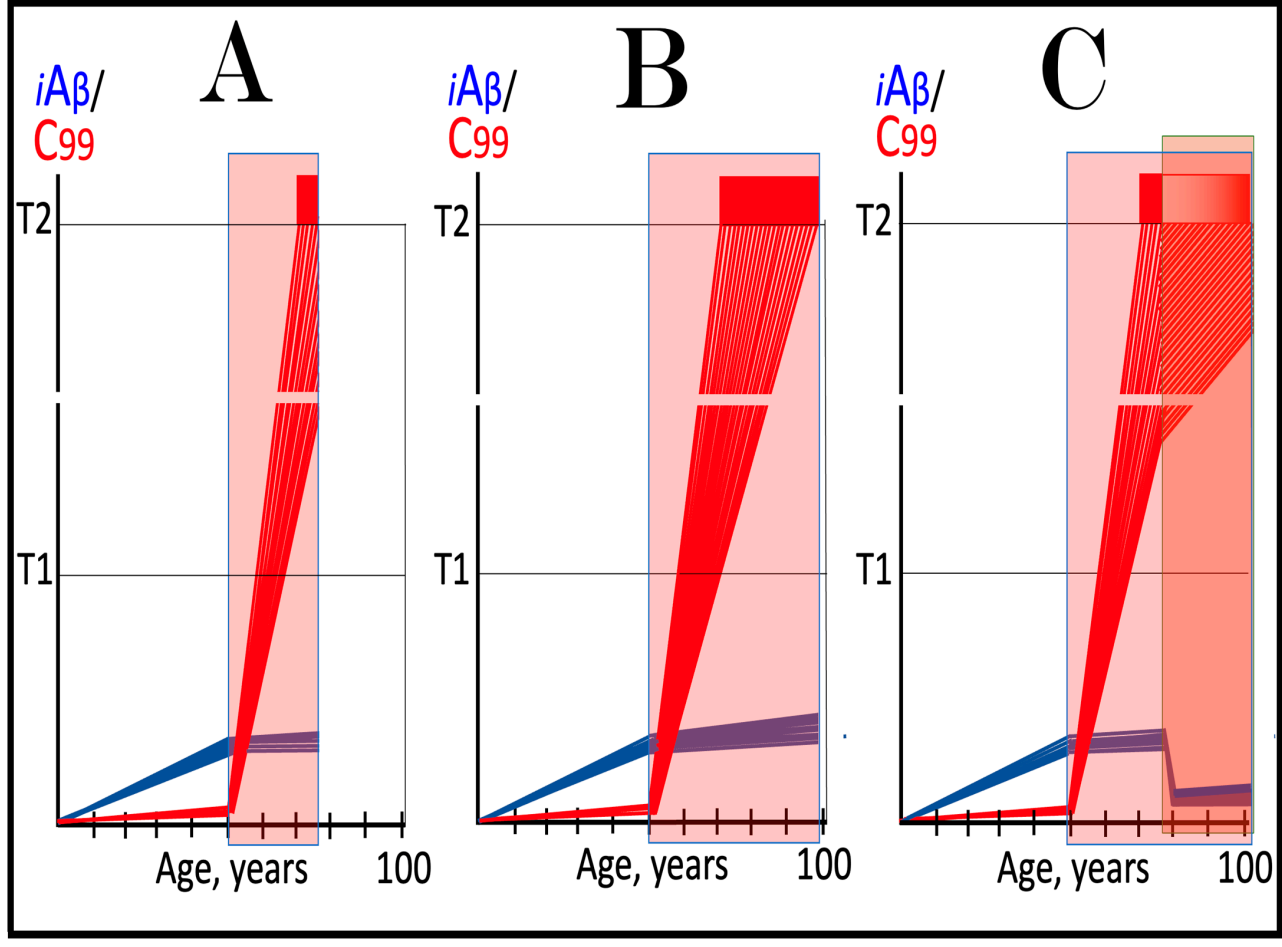
**Long-term targeted degradation of iAβ and C99 in the treatment of unconventional AD**. *Vertical axis*: Relative levels of iAβ and C99. *Horizontal axis*: Age, in years. *Blue lines*: Dynamics of iAβ. *Red lines*: Dynamics of C99. **T1**: Threshold of intracellular concentration of iAβ or C99 that mediates elicitation of the neuronal integrated stress response, which, in turn, enables operation of the AβPP-independent C99 generation pathway and thus triggers the disease. **T2**: Threshold of intracellular concentration of C99 that triggers neuronal death. *Red box*: Range of intracellular concentrations of C99, dubbed the “Apoptotic Zone”, associated with apoptosis or necroptosis of neuronal cells. *Pink boxes*: Duration of the occurrence of unconventional stressors at concentrations sufficient to elicit the neuronal ISR. *Orange box*: Duration of the targeted iAβ/C99 degradation treatment. Panel (**A**): The initial state of levels of iAβ and C99 at the commencement of the treatment. The neuronal ISR has been unconventionally elicited, and the AβPP-independent C99 production pathway has been unconventionally activated at levels of iAβ below the T1 threshold. C99 has rapidly accumulated, crossed the T1 threshold, and AD commenced. The accumulation has continued, C99 levels have reached and crossed the T2 threshold, and AD symptoms have manifested. Panel (**B**): Evolution of the initial state in the absence of the treatment. Accumulation of C99 produced in the AβPP-independent pathway continues unabated. The disease progresses and reaches its end stage. Panel (**C**): Evolution of the initial state under the long-term iAβ/C99 degradation treatment. iAβ is rapidly depleted, and its levels remain low for the duration of the treatment. The rate of C99 degradation is below that of its production in the AβPP-independent pathway. Its accumulation continues, but at a lower rate. The progression of the disease is not stopped. It proceeds, but at a reduced rate, for the duration of the treatment.

Effects of the long-term targeted degradation of iAβ and of the Aβ segment of C99 are illustrated in Figure 17. Panel A of Figure 17 depicts the initial state of levels of iAβ and C99 at the start of the treatment. At this point, the neuronal ISR has been unconventionally elicited, and the AβPP-independent C99 production pathway has been unconventionally activated at levels of iAβ below the T1 threshold. C99 has rapidly accumulated and crossed the T1 threshold, and AD commenced. The accumulation has continued, C99 levels have reached and crossed the T2 threshold, and AD symptoms have manifested. Panel B of Figure 17 shows the evolution of the initial state in untreated patients. In the absence of the treatment, the accumulation of C99 continues unimpeded. The T2 threshold is crossed in additional neurons, and the disease reaches its end stage.

Panel C of Figure 17 depicts the evolution of the initial state in unconventional AD patients treated with an agent capable of the targeted degradation of iAβ and of the Aβ segment of C99. Under the treatment, iAβ is being depleted, and its accumulation is reversed. On the other hand, the rate of production of C99 in the AβPP-independent pathway exceeds that of its degradation. Accordingly, the influx of C99 occurs at a greater rate than its efflux. Consequently, the accumulation of C99 and the progression of the disease continue, albeit at a decreased rate, for the duration of the treatment. The bottom line is that the long-term treatment with an agent capable of targeted degradation of iAβ and the Aβ segment of C99 would certainly be beneficial both preventively and curatively. The benefits, however, would be partial: the treatment, administered prior to the T1 crossing, would delay but not preclude the occurrence of the disease, and, when implemented in symptomatic AD, would only slow down, and not arrest, its progression.

## 17. Transient Targeted Degradation of iAβ and of the Aβ Segment of C99 in the Prevention and Treatment of Unconventional AD

**Figure 18:**
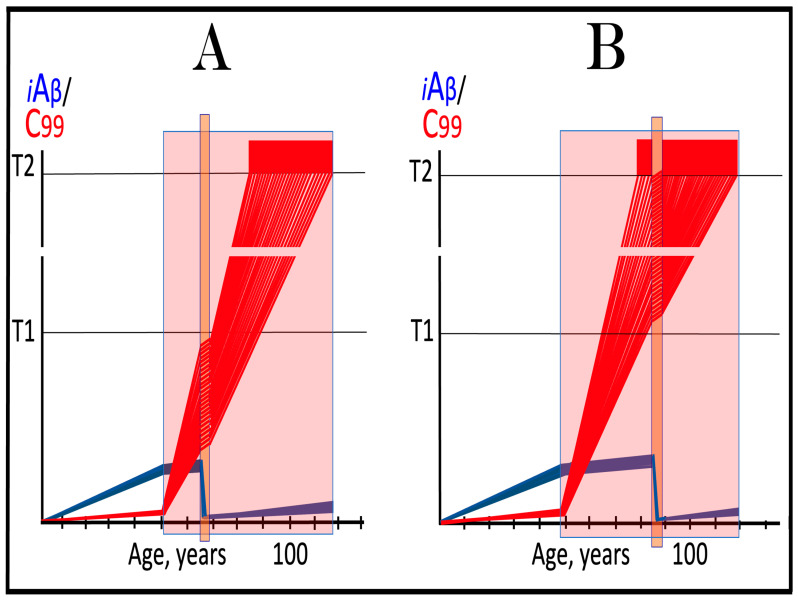
**Transient iAβ/C99 degradation therapy in the prevention and treatment of unconventional AD**. *Vertical axis*: Relative levels of iAβ and C99. *Horizontal axis*: Age, in years. *Blue lines*: Dynamics of iAβ. *Red lines*: Dynamics of C99. **T1**: Threshold of intracellular concentration of iAβ or C99 that mediates elicitation of the neuronal integrated stress response, which, in turn, enables operation of the AβPP-independent C99 generation pathway and thus triggers the disease. **T2**: Threshold of intracellular concentration of C99 that triggers neuronal death. *Red box*: Range of intracellular concentrations of C99, dubbed the “Apoptotic Zone”, associated with apoptosis or necroptosis of neuronal cells. *Pink boxes*: Duration of the occurrence of unconventional stressors at concentrations sufficient to elicit the neuronal ISR. *Orange box*: Duration of the targeted iAβ/C99 degradation treatment. Panel (**A**): Transient iAβ/C99 degradation therapy in the prevention of unconventional AD. At the commencement of the treatment, the neuronal ISR has already been unconventionally elicited, and the AβPP-independent pathway unconventionally activated at low under-T1 levels of iAβ. Under ISR conditions, accumulation of iAβ has been suppressed. Simultaneously, C99 produced independently of AβPP has rapidly accumulated but has not yet reached the T1 threshold. The transient iAβ/C99 degradation treatment depletes iAβ to nearly basal levels, but accumulation of C99 produced independently of AβPP continues, albeit at a reduced rate. Following the transient treatment, accumulation of both iAβ and C99 resumes at the pre-treatment rates. C99 crosses the T1 threshold, and AD commences and progresses. Thus, the treatment only delayed the T1 crossing and the disease by less than its duration (because post-treatment accumulation of C99 resumed from the elevated baseline). Panel (**B**): Transient iAβ/C99 degradation therapy in the treatment of unconventional AD. At the commencement of the treatment, the neuronal ISR has already been unconventionally elicited, and the accumulation of iAβ has been suppressed. Concurrently, the AβPP-independent C99 production pathway has been activated. When C99 crossed the T1 threshold, the disease commenced, and when it reached the T2 threshold in a fraction of the affected neurons, AD symptoms manifested. iAβ and C99 degradation depletes the former but only slows down accumulation of the latter. Following the transient treatment, accumulation of both resumes at the pre-treatment rates. Progression of the disease also resumes at the pre-treatment rate. Thus, the reprieve provided by the treatment lasts no longer than its duration.

While the long-term targeted degradation of iAβ and of the Aβ segment of C99 would be partially beneficial in the prevention and treatment of unconventional AD, the same treatment administered transiently would result in only marginal benefits for treated individuals. The reasons for this are illustrated in Figure 18. Panel A of Figure 18 depicts the effects of the transient targeted iAβ/C99 degradation treatment administered prior to the T1 crossing. At the time of the treatment, the neuronal ISR has already been unconventionally elicited, and the AβPP-independent pathway unconventionally activated at low under-T1 levels of iAβ. Under ISR conditions, accumulation of iAβ has been suppressed. At the same time, C99 produced independently of AβPP has rapidly accumulated but has not yet reached the T1 threshold. When implemented, the transient iAβ/C99 degradation treatment depletes iAβ and causes the reversal of its accumulation. On the other hand, for the reasons discussed above, it only reduces the rate of C99 accumulation but does not reverse it. When the drug is withdrawn, the accumulation of iAβ resumes at the pre-treatment rate. More importantly, the accumulation of C99 also resumes at the pre-treatment rate and from an elevated baseline. It would cross the T1 threshold, and the disease would commence and progress. Thus, the transient targeted iAβ/C99 degradation treatment would provide only marginal preventive benefits lasting no longer than its duration.

Panel B of Figure 18 illustrates the effects of the transient targeted iAβ/C99 degradation treatment administered in symptomatic disease. When the treatment is implemented, the neuronal ISR has already been unconventionally elicited; as a result, the accumulation of iAβ has been suppressed. Simultaneously, the production of C99 in the AβPP-independent pathway has been activated. It rapidly accumulated, crossed the T1 threshold, and the disease commenced. C99 continued to accumulate, crossed the T2 threshold in a fraction of the neurons, and AD symptoms manifested. The iAβ/C99 degradation treatment reverses the accumulation of iAβ. On the other hand, C99 continues to accumulate, albeit at a reduced rate, and the rate of the progression of the disease is reduced accordingly. Upon completion of the transient treatment, accumulation of iAβ resumes at the pre-treatment rate. Importantly, accumulation of C99 also resumes, from an elevated baseline, at the pre-treatment rate. Consequently, the progression of the disease would also resume at the pre-treatment rate. Thus, the transient iAβ/C99 degradation treatment would provide only a marginal curative reprieve lasting no longer than its duration. It should be emphasized that, for the reasons discussed in the preceding section, activators of BACE1 and/or BACE2 are inapplicable as iAβ/C99 degrading agents in the scenario under discussion.

## 18. Transient Composite iAβ and C99 Degradation/ISR Suppression Therapy in the Prevention and Treatment of Unconventional AD

The therapeutic strategy discussed in the preceding section, namely, transient targeted iAβ/C99 degradation treatment, yields only marginal benefits because it does not deplete C99 (actually, it allows its accumulation, albeit at a reduced rate, even during the treatment). The reason for this is that the rate of the influx of C99 produced in the AβPP-independent pathway is such that it cannot be matched by the rate of iAβ/C99 degradation. A solution to this problem is to carry out C99 degradation concurrently with the reduction, and preferably the arrest, of its influx. The latter can be accomplished by suppression of the neuronal ISR. Indeed, the reversal of the neuronal ISR state would deprive the AβPP-independent C99 production pathway of its essential components and its operation, along with the influx of its C99 product, would cease. As described above, no long-term neuronal ISR inhibition is currently feasible (otherwise, as discussed above, it would be therapeutically sufficient for the prevention and treatment of AD, and no C99 degradation therapy would be needed). On the other hand, transient suppression of the ISR is feasible and can be used in combination with targeted iAβ/C99 degradation therapy. Importantly, activators of BACE1 and/or BACE2 are perfectly applicable as iAβ/C99 degradation agents in this approach for the duration of ISR inhibition. The transient component of this composite treatment is the ISR inhibition. iAβ/C99 degrading agents can be administered long-term, but activators of BACE1 and/or BACE2 would be inefficient under the neuronal ISR re-elicited upon the removal of ISR-inhibiting drugs. Therefore, as illustrated below, long-term administration of iAβ/C99 degrading agents in combination with transient suppression of the neuronal ISR would be the preferred therapeutic strategy for the prevention and treatment of unconventional AD.

### 18.1. Transient Composite iAβ and C99 Degradation/ISR Suppression Therapy in the Prevention of Unconventional AD

**Figure 19:**
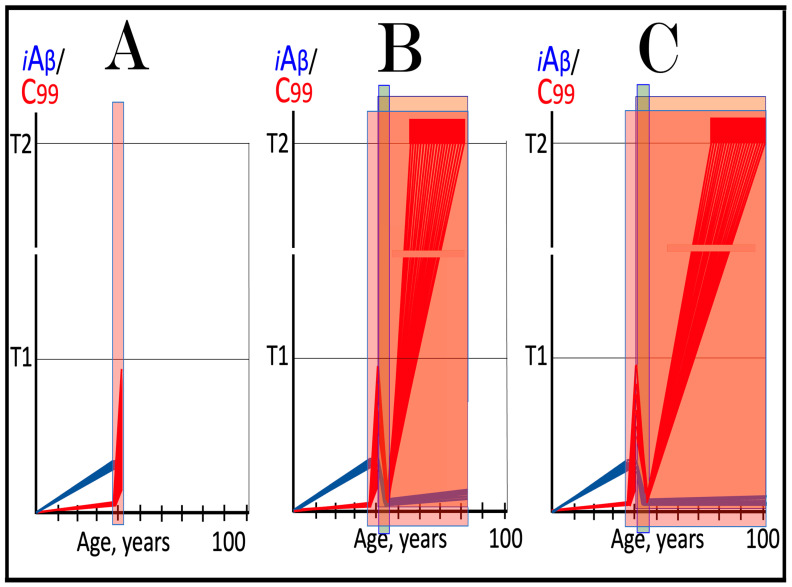
**Transient composite iAβ and C99 degradation/ISR suppression therapy in the prevention of unconventional AD**. *Vertical axis*: Relative levels of iAβ and C99. *Horizontal axis*: Age, in years. *Blue lines*: Dynamics of iAβ. *Red lines*: Dynamics of C99. **T1**: Threshold of intracellular concentration of iAβ or C99 that mediates elicitation of the neuronal integrated stress response, which, in turn, enables operation of the AβPP-independent C99 generation pathway and thus triggers the disease. **T2**: Threshold of intracellular concentration of C99 that triggers neuronal death. *Red box*: Range of intracellular concentrations of C99, dubbed the “Apoptotic Zone”, associated with apoptosis or necroptosis of neuronal cells. *Pink box*: Duration of the occurrence of unconventional stressors at concentrations sufficient to elicit the neuronal ISR. *Green box*: Duration of the ISR suppression treatment. *Orange box*: Duration of the targeted iAβ/C99 degradation treatment. Panel (**A**): The initial state of levels of iAβ and C99 at the commencement of the transient composite iAβ and C99 degradation/ISR suppression treatment. The neuronal ISR has already been elicited by an unconventional stressor at low, under-T1, levels of iAβ, and the accumulation of the latter has been suppressed. Concurrently, the AβPP-independent C99 production pathway has been unconventionally activated. C99 produced independently of AβPP has rapidly accumulated, but its levels are still below the T1 threshold. If no therapeutic treatments were implemented, accumulation of C99 produced in the AβPP-independent pathway would proceed unhindered; when its levels cross the T1 threshold, the disease would commence and progress. Panel (**B**): Evolution of the initial state under and following the transient composite BACE1 and/or BACE2 activation/ISR inhibition treatment. The transient component of this composite treatment is ISR inhibition, which would be deleterious if administered long-term. BACE activators can remain long-term, but would be rendered ineffective by the removal of ISR inhibitors. Under the treatment, the neuronal ISR state is reversed. With the supply of its essential components abrogated, the AβPP-independent C99 production pathway is disabled, and the influx of its product, C99, ceases. Temporary reversal of the neuronal ISR state also restores the production of BACE1 and BACE2. Under these circumstances, the activator-enhanced intra-Aβ cleaving activities of BACE1 and/or BACE2 effectively deplete both iAβ and C99 to basal levels. Upon removal of the ISR-inhibiting drug, the neuronal ISR is re-elicited and the AβPP-independent C99 production pathway is re-activated. Accumulation of iAβ and C99 resumes from low baselines and proceeds at the pre-treatment rates. When levels of C99 cross the T1 threshold, the disease commences. The treatment provides a temporary delay of the disease, potentially measured in years. Panel (**C**): Evolution of the initial state under and following the transient composite iAβ and C99 degradation/ISR inhibition treatment, with a degradation agent other than BACE and/or BACE2 activators. The only difference from Panel (**B**) is that, following the removal of ISR inhibitors, re-elicitation of the neuronal ISR and re-activation of the AβPP-independent C99 production pathway, iAβ/C99 degradation agent remains operational. Consequently, levels of iAβ remain low, and accumulation of C99 resumes at a rate below that prior to the treatment. The delay in commencement of AD would be greater and the progression of the disease slower than with BACE activators as iAβ-degrading agents.

Outcomes of the transient composite iAβ and C99 degradation/ISR inhibition therapy in the prevention of unconventional AD are considered in Figure 19. Panel a of Figure 19 depicts the initial state of levels of iAβ and C99 at the commencement of the transient composite iAβ and C99 degradation/ISR suppression treatment. At this time, the neuronal integrated stress response has already been elicited by an unconventional stressor at low, under-T1, levels of iAβ, and the accumulation of the latter has been suppressed. Concurrently, the AβPP-independent C99 production pathway has been unconventionally activated. C99 produced independently of AβPP has rapidly accumulated, but its levels are still below the T1 threshold. If no therapeutic treatment were implemented, this initial state would evolve as follows: accumulation of C99 produced in the AβPP-independent pathway would proceed unhindered; when its levels cross the T1 threshold, the disease would commence and progress.

Panel B of Figure 19 shows the evolution of the initial state under and following the transient composite BACE1 and/or BACE2 activation/ISR inhibition treatment. Under the treatment, the neuronal ISR state is reversed. With the supply of its essential components abrogated, the AβPP-independent C99 production pathway is disabled, and the influx of its product, C99, ceases. Reversal of the neuronal ISR state also restores the production of BACE1 and BACE2. Under these circumstances, the activator-enhanced intra-Aβ cleaving activities of BACE1 and/or BACE2 effectively deplete both iAβ and, more importantly, C99 to basal levels. The transient component of this composite treatment is ISR inhibition, which would be deleterious if administered long-term. BACE activators can remain for a long duration, but are rendered ineffective by the removal of ISR inhibitors. When the ISR-inhibiting drug is withdrawn, accumulation of iAβ resumes from a low baseline and proceeds at the pre-treatment rate. As for C99, the cessation of ISR inhibition triggers re-elicitation of the neuronal ISR and re-activation of the AβPP-independent C99 production pathway. The accumulation of C99 resumes and, since production of BACE1 and BACE2 is suppressed under ISR conditions and their activators are rendered ineffective, proceeds at the pre-treatment rate. When its levels cross the T1 threshold, the disease commences. Nevertheless, the treatment provides reprieve from AD, potentially measured in years.

Panel C of Figure 19 illustrates the evolution of the initial state under and following the transient composite iAβ and C99 degradation/ISR inhibition treatment, where the degradation agent differs from BACE and/or BACE2 activators. In this scenario, in contrast to the situation described in the preceding paragraph and illustrated in panel B of Figure 19, the iAβ/C99 degrading agent is operational under ISR conditions and retains its activity when the ISR-inhibiting drug is removed following the transient composite treatment (as a reminder, the transient component of the composite treatment is only suppression of the neuronal ISR). In the present scenario, the role of the ISR inhibition is solely to disable the AβPP-independent C99 generation pathway and to abrogate the influx of its product. With this accomplished, the iAβ/C99 degradation agent would effectively deplete both iAβ and C99 to basal levels. Following the withdrawal of the ISR-inhibiting drug, the neuronal ISR is re-elicited, the AβPP-independent C99 production pathway re-activated, and accumulation of both iAβ and C99 resumes from low baselines. Because the iAβ/C99 degrading drug remains in the system and retains its activity under ISR conditions, the rates of accumulation of iAβ and C99 are significantly lower than prior to the composite treatment. Accordingly, the therapeutic benefits conferred by such a treatment would potentially be of much greater duration than those yielded by the composite therapy employing BACE1 and/or BACE2 activators as iAβ-degrading agents.

### 18.2. Transient Composite iAβ and C99 Degradation/ISR Suppression Therapy in the Treatment of Unconventional AD

**Figure 20:**
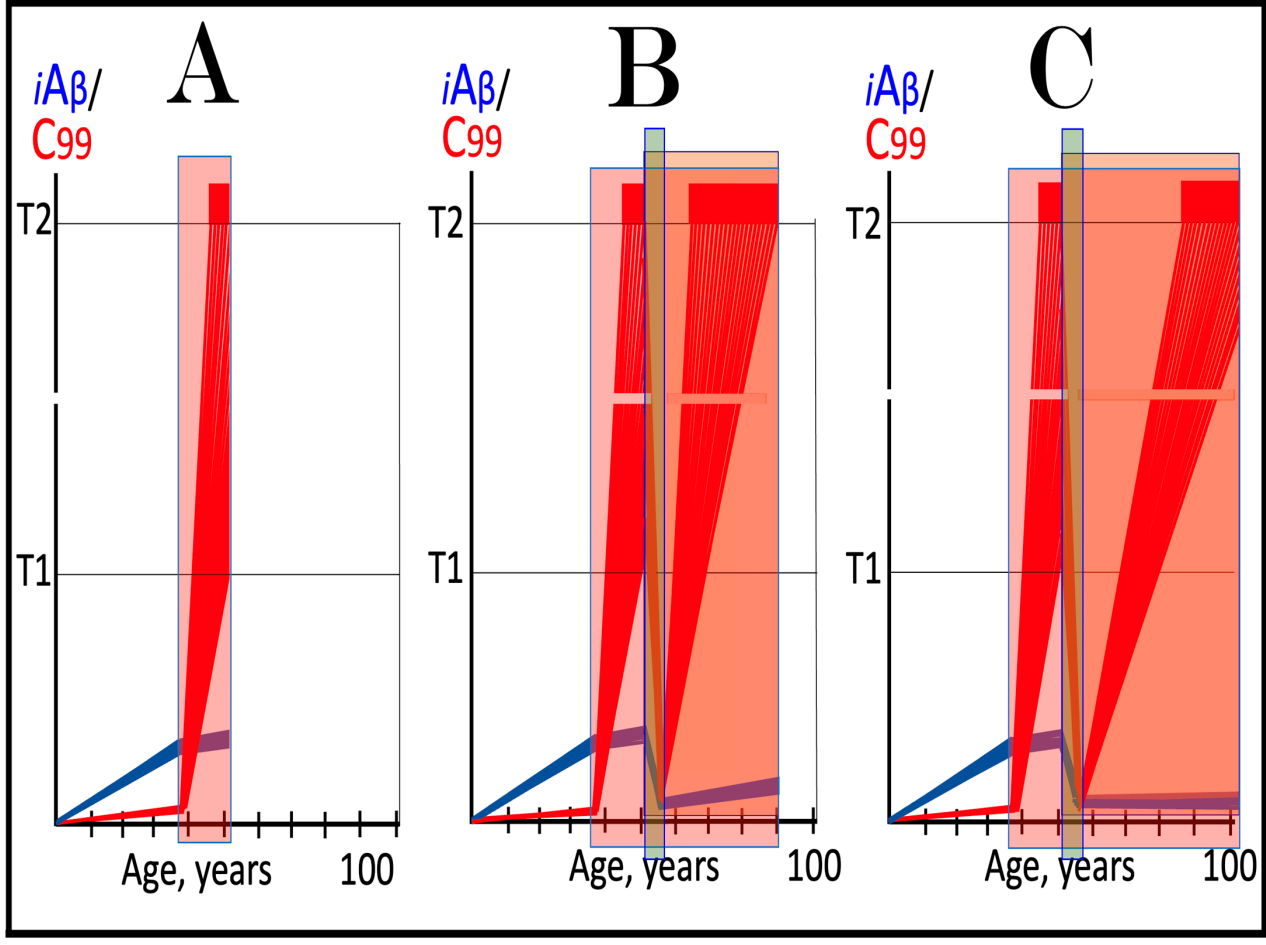
**Transient composite iAβ and C99 degradation/ISR suppression therapy in the treatment of unconventional AD**. *Vertical axis*: Relative levels of iAβ and C99. *Horizontal axis*: Age, in years. *Blue lines*: Dynamics of iAβ. *Red lines*: Dynamics of C99. **T1**: Threshold of intracellular concentration of iAβ or C99 that mediates elicitation of the neuronal integrated stress response, which, in turn, enables operation of the AβPP-independent C99 generation pathway and thus triggers the disease. **T2**: Threshold of intracellular concentration of C99 that triggers neuronal death. *Red box*: Range of intracellular concentrations of C99, dubbed “Apoptotic Zone”, associated with apoptosis or necroptosis of neuronal cells. *Pink box*: Duration of the occurrence of unconventional stressors at concentrations sufficient to elicit the neuronal ISR. *Green box*: Duration of the ISR suppression treatment. *Orange box*: Duration of the targeted iAβ/C99 degradation treatment. Panel (**A**): The initial state of levels of iAβ and C99 at the commencement of the transient composite iAβ and C99 degradation/ISR suppression treatment. The neuronal ISR has already been elicited by an unconventional stressor at low, under-T1, levels of iAβ, and the accumulation of the latter has been suppressed. Concurrently, the AβPP-independent C99 production pathway has been unconventionally activated. C99 produced independently of AβPP has rapidly accumulated, crossed the T1 threshold, and reached the T2 threshold in a fraction of the affected neurons, and AD symptoms have manifested. If the patient were not treated, the disease would progress unimpeded. Panel (**B**): Evolution of the initial state under and following the transient composite BACE1 and/or BACE2 activation/ISR inhibition treatment. The transient component of this composite treatment is ISR inhibition, which would be deleterious if administered long-term. BACE activators can remain long-term but would be rendered ineffective by the removal of ISR inhibitors and re-establishment of the ISR state. Under the treatment, the neuronal ISR state is reversed. With the supply of its essential components abrogated, the AβPP-independent C99 production pathway is disabled, and influx of its product, C99, ceases. Temporary reversal of the neuronal ISR state also restores the production of BACE1 and BACE2. Under these circumstances, the activator-enhanced intra-Aβ cleaving activities of BACE1 and/or BACE2 effectively deplete both iAβ and C99 to basal levels. Upon removal of the ISR-inhibiting drug, the neuronal ISR is re-elicited and the AβPP-independent C99 production pathway is re-activated. Accumulation of iAβ and C99 resumes from low baselines and proceeds at the pre-treatment rates. When levels of C99 cross the T1 threshold, the disease recurs and progresses at the pre-treatment rate. The treatment provides temporary delay of the recurrence of the disease, potentially measured in years. Panel (**C**): Evolution of the initial state under and following the transient composite iAβ and C99 degradation/ISR inhibition treatment with the degradation agent other than BACE and/or BACE2 activators. The only difference from Panel (**B**) is that, following the removal of ISR inhibitors, re-elicitation of the neuronal ISR and re-activation the AβPP-independent C99 production pathway, iAβ/C99 degradation agent remains operational. Consequently, levels of iAβ remain low and accumulation of C99 resumes at the rate below that prior to the treatment. The delay in commencement of AD would be greater and progression of the disease slower than with BACE activators as iAβ-degrading agents.

Effects of the transient composite iAβ and C99 degradation/ISR inhibition therapy in the treatment of unconventional AD are illustrated in Figure 20. As in the preceding sub-section, the transient component of such a treatment is the suppression of the neuronal ISR, which would be detrimental if implemented long-term. The degradation of iAβ and C99 can be carried out long-term, but would be ineffective under ISR conditions if activators of BACE1 and/or BACE2 were employed as degradation agents. Panel A of Figure 20 depicts the initial state of levels of iAβ and C99 at the start of the transient composite iAβ and C99 degradation/ISR inhibition treatment. At this time, the neuronal integrated stress response has already been elicited by an unconventional stressor at low, under-T1, levels of iAβ. The accumulation of iAβ has been suppressed. Simultaneously, the AβPP-independent C99 production pathway has been unconventionally activated. C99 produced independently of AβPP has rapidly accumulated and crossed the T1 threshold, and AD commenced. The rapid accumulation of C99 produced independently of AβPP has continued; it crossed the T2 threshold in a fraction of the neurons, and AD symptoms have manifested. If the patient were not treated, the disease would progress unimpeded; when the T2 threshold is crossed in a sufficient fraction of the neurons, the disease would enter its end stage.

Panel B of Figure 20 shows the evolution of the initial state during and following the transient composite BACE1 and/or BACE2 activation/ISR inhibition treatment. As described above, inhibition of the neuronal ISR disables the AβPP-independent C99 generation pathway by abrogating production of its essential components. As a result, the influx of C99 produced independently of AβPP is interrupted. Inhibition of the neuronal ISR also enables the production of BACE1 and BACE2, which are suppressed under ISR conditions. Activators of BACE1 and/or BACE2, administered concurrently with ISR inhibitors, mediate effective depletion of iAβ and C99. When the ISR-inhibiting drug is removed, the de novo accumulation of iAβ and C99 resumes. The removal of the ISR-inhibiting drug results in the re-elicitation of ISR. Under ISR conditions, the production of BACE1 and BACE2 is suppressed, their activators are ineffective, and accumulation of iAβ and C99 proceeds at the pre-treatment rates. Levels of C99 rapidly increase, and when they cross the T1 threshold, the disease recurs and progresses. The treatment provides only temporary relief, but it is still measured, probably, in years.

Panel C of Figure 20 details the evolution of the initial state when the composite treatment described above employs iAβ and C99 degradation agents other than activators of BACE1 and/or BACE2. The advantage of this type of iAβ and C99 degradation agents is that they fully retain their activity under ISR conditions, and their continuous employment, following transient inhibition of the ISR, can confer significant therapeutic benefits. As shown in panel C of Figure 20, the suppression of the neuronal ISR disables the AβPP-independent C99 generation pathway and thus abrogates the influx of C99 produced independently of AβPP. Under these conditions, iAβ and C99 degradation agents efficiently deplete both iAβ and C99 to basal levels. When ISR inhibitors are withdrawn, the AβPP-independent C99 production pathway is re-activated. Accumulation of iAβ and C99 resumes from low baselines. Since iAβ and C99 degradation agents remain in the system and retain their activity under ISR conditions, the rates of accumulation of iAβ and C99 would be significantly lower than prior to the treatment. Consequently, this approach would yield therapeutic benefits of potentially much greater duration than those conferred by activators of BACE1 and/or BACE2 as iAβ and C99 degradation agents.

## 19. Recurrent Implementation of the Transient Composite iAβ and C99 Degradation/ISR Inhibition Treatment Can Greatly Extend the Duration of Its Therapeutic Benefits

As described in the preceding sections, the principal advantage of the transient composite BACE1 and BACE2 activation/ISR inhibition treatment is that it is capable of depleting both iAβ and C99 to basal levels. When transiently administered, ISR inhibitors are withdrawn, the neuronal ISR is re-elicited, and the AβPP-independent C99 generation pathway is re-activated. However, BACE1 and BACE2 activators are rendered ineffective by the ISR state, and the accumulation of C99 resumes from a low baseline but at the pre-composite treatment rate. Nevertheless, due to its depletion, its de novo accumulation to the T1 level requires considerable time, probably measured in years. This time period constitutes the duration of therapeutic benefits conferred by the treatment. The duration of therapeutic benefits can be significantly extended if iAβ and C99 degradation agents other than BACE1 and BACE2 activators are employed concurrently with and following the withdrawal of ISR inhibitors. This is because, unlike BACE activators, these degradation agents retain their activity under ISR conditions. Therefore, in this approach, the rate of accumulation of C99 produced independently of AβPP following the re-elicitation of the neuronal ISR and re-activation of the AβPP-independent C99 generation pathway is significantly lower, and, consequently, the duration of therapeutic benefits is significantly greater than in the composite treatment employing BACE1 and BACE2 activators as iAβ and C99 degradation agents.

**Figure 21:**
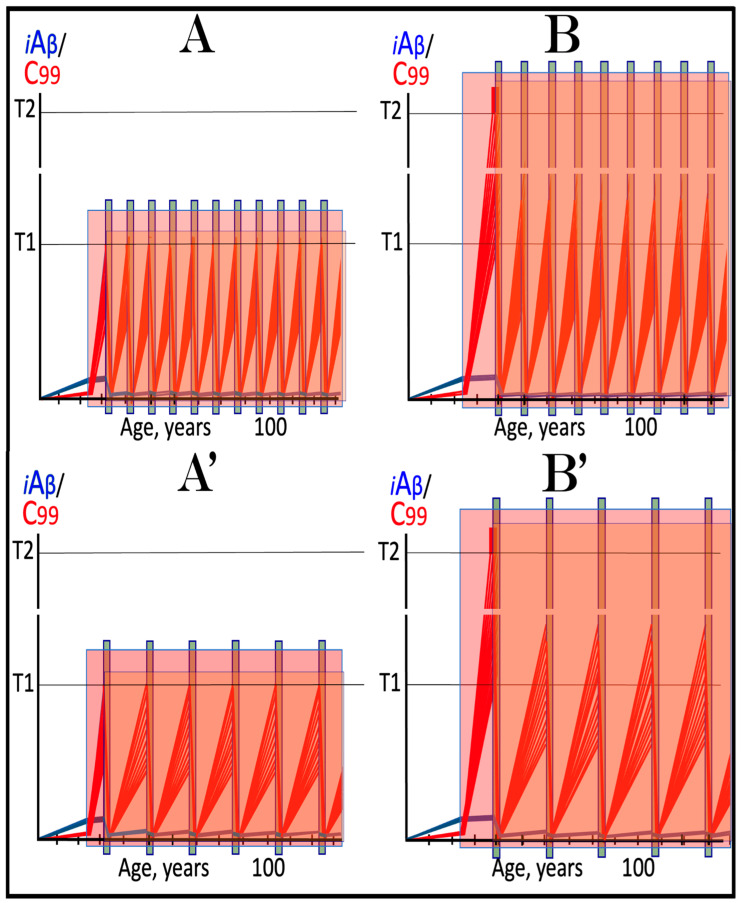
**Recurrent implementation of the transient composite iAβ and C99 degradation/ISR inhibition treatment extends the duration of its therapeutic benefits**. *Vertical axis*: Relative levels of iAβ and C99. *Horizontal axis*: Age, in years. *Blue lines*: Dynamics of iAβ. *Red lines*: Dynamics of C99. **T1**: Threshold of intracellular concentration of iAβ or C99 that mediates elicitation of the neuronal integrated stress response, which, in turn, enables operation of the AβPP-independent C99 generation pathway and thus triggers the disease. **T2**: Threshold of intracellular concentration of C99 that triggers neuronal death. *Red box*: Range of intracellular concentrations of C99, dubbed “Apoptotic Zone”, associated with apoptosis or necroptosis of neuronal cells. *Pink box*: Duration of the occurrence of unconventional stressors at concentrations sufficient to elicit the neuronal ISR. *Green box*: Duration of the ISR suppression treatment. *Orange box*: Duration of the targeted iAβ/C99 degradation treatment. The transient component of this composite treatment is ISR inhibition, which would be deleterious if administered long-term. iAβ/C99 degradation agents are administered long-term, but BACE activators are rendered ineffective by the removal of ISR inhibitors and re-establishment of the ISR state. Panel (**A**): Effects of the recurrent transient composite BACE1 and/or BACE2 activation/ISR inhibition therapy in the prevention of unconventional AD. By the time of the initial implementation of the transient ISR suppression treatment, the neuronal ISR has been unconventionally elicited, and the AβPP-independent C99 production pathway is unconventionally activated. Under ISR conditions, the accumulation of iAβ has been suppressed, but C99 produced independently of AβPP has rapidly accumulated; its levels have not yet reached the T1 threshold. ISR inhibitors administered at this point reverse the ISR state, disable the AβPP-independent C99 production pathway, interrupt the influx of C99, and enable the production of BACE1 and BACE2. Concurrently administered BACE1 and/or BACE2 inhibitors mediate efficient depletion of iAβ and C99. When ISR inhibitors are withdrawn, the neuronal ISR is re-elicited, the AβPP-independent C99 generation pathway is re-activated, BACE activators are rendered ineffective by the ISR state, and the accumulation of iAβ and C99 produced independently of AβPP resumes from low baselines and proceeds at the pre-initial treatment rates. Before C99 reaches the T1 threshold, at the time determined by appropriate biomarkers, the treatment is repeated as many times as needed. As a result, no T1 would be crossed, and no AD would occur within the lifespan of the recurrently treated individual. Panel (**B**): Effects of the recurrent transient composite BACE1 and/or BACE2 activation/ISR inhibition therapy in the treatment of unconventional AD. By the time of the initial implementation of the transient ISR suppression treatment, the neuronal ISR has been unconventionally elicited, and the AβPP-independent C99 production pathway has been unconventionally activated. Under ISR conditions, the accumulation of iAβ has been suppressed, but C99 produced independently of AβPP has rapidly accumulated. When it crossed the T1 threshold, AD commenced; when the T2 threshold was crossed in a fraction of the affected neurons, AD symptoms manifested. With the neuronal ISR reversed by its inhibitors, BACE1 and/or BACE2 activators mediate effective depletion of both iAβ and C99. When ISR inhibitors are withdrawn, the neuronal ISR is re-elicited by unconventional stressors, BACE activators are rendered ineffective, and the AβPP-independent C99 generation pathway is re-activated. Accumulation of iAβ and C99 resumes from low baselines and proceeds at the pre-initial treatment rates. Before its levels reach the AD pathology-causing range, at the time point defined by appropriate biomarkers, the transient composite BACE activation/ISR inhibition treatment is re-administered and then repeated as needed. No AD pathology-driving range of C99 concentrations would be reached, and AD would not recur for the duration of the recurrent treatments. Panels (**A’**) and (**B’**) are conceptually identical, with only one difference, to panels (**A**) and (**B**), respectively. The difference is that the iAβ/C99 degradation agents employed are distinct from BACE activators and presumably retain their activity under ISR conditions. This difference plays out following the withdrawal of transiently administered ISR inhibitors. At this point, the neuronal ISR is re-elicited by unconventional stressors, and the AβPP-independent C99 generation pathway is re-activated, but iAβ/C99 degradation agents remain operational. iAβ remains at low levels, whereas accumulation of C99 resumes from a low baseline and occurs *slower* than prior to the initial treatment due to persistent activity of iAβ/C99 degradation agents. Consequently, the duration of therapeutic benefits for each treatment would be much longer and the number of required recurrent treatments much smaller in this approach, in both its preventive and curative applications, than in the one utilizing BACE activators as iAβ/C99 degradation agents.

The duration of therapeutic benefits of the transient composite iAβ and C99 degradation/ISR inhibition treatment can be greatly extended, in fact, multiplied many times by the recurrent implementation of the treatment. In principle, the treatment can be repeated, as many times as needed, for the remaining lifespan, with the timing of its implementation guided by the appropriate biomarkers. This approach is illustrated in Figure 21. Panels A and B of Figure 21 detail outcomes of the recurrent transient composite BACE1 and/or BACE2 activation/ISR inhibition treatments. As specified above, the transient component of such a treatment is the ISR inhibition, which would be detrimental if administered long-term. BACE activators, on the other hand, could remain in the system long-term but would be rendered ineffective under ISR conditions, which suppress the production of both BACE1 and BACE2. In practical terms, they would probably be withdrawn together with ISR inhibitors when C99 is sufficiently depleted.

In panel A of Figure 21, by the time of the initial administration of the transient composite BACE activation/ISR inhibition treatment, the neuronal ISR has been unconventionally elicited, and the AβPP-independent C99 production pathway has been unconventionally activated. Under ISR conditions, the accumulation of iAβ has been suppressed. On the other hand, C99 produced independently of AβPP has rapidly accumulated but has not yet reached the T1 threshold. ISR inhibitors administered at this point reverse the ISR state, disable the AβPP-independent C99 production pathway, interrupt the influx of C99, and enable the production of BACE1 and BACE2. Under these circumstances, concurrently administered BACE1 and/or BACE2 activators mediate efficient depletion of iAβ and C99. When drugs are withdrawn, the neuronal ISR is re-elicited, the AβPP-independent C99 generation pathway is re-activated, and the accumulation of C99 produced independently of AβPP resumes from a low baseline and proceeds at the pre-treatment rate. Before it reaches the T1 threshold, at the time point determined by appropriate biomarkers, the treatment is repeated recurrently as many times as needed. As a result, no T1 would be crossed, and no AD would occur within the lifespan of the recurrently treated individual.

In panel B of Figure 21, at the time of the initial transient composite BACE activation/ISR inhibition treatment, C99 produced in the unconventionally activated pathway has already crossed the T1 threshold, and AD commenced. C99 continued to accumulate, crossed the T2 threshold in a fraction of the neurons, and AD symptoms manifested. Concurrently administered ISR inhibitors and BACE1 and/or BACE2 activators perform the same functions in this scenario as described in the preceding paragraph above. Following the initial transient treatment, when drugs are withdrawn, the progression of the disease ceases, and the accumulation of C99 produced in the re-activated AβPP-independent pathway resumes de novo from a low baseline and proceeds at the pre-treatment rate. Eventually, it crosses the T1 threshold, but before its levels reach the AD pathology-causing range, at the time point defined by appropriate biomarkers, the transient composite BACE activation/ISR inhibition treatment is re-administered and then repeated as needed. No AD pathology-driving range of C99 concentrations would be reached and AD would not recur for the duration of the recurrent treatments.

Panels A’ and B’ of Figure 21 illustrate effects of the recurrently administered transient composite iAβ and C99 degradation/ISR inhibition treatments where the degradation agents employed are other than BACE1 and/or BACE2 activators. The outcomes of this approach, in both the prevention (panel A’ of Figure 21) and treatment (panel B’ of Figure 21) of unconventional AD, are principally the same as in the strategy utilizing BACE activators as iAβ and C99 degradation agents. The only difference is the length of the time intervals between recurrently administered transient treatments, defined above as the duration of therapeutic benefits. In the presently discussed approach, this duration is substantially longer. This is because iAβ and C99 degradation agents, which are not withdrawn together with ISR inhibitors but are administered long-term, fully retain their activity under ISR conditions. Therefore, following each transient ISR inhibition treatment, when the neuronal ISR is re-elicited and the AβPP-independent C99 production pathway re-activated, the de novo accumulation of C99 resumes from a low baseline and, importantly, proceeds at the rate significantly lower than that prior to the initial treatment. Consequently, the duration of therapeutic benefits for each treatment would be much longer and the number of required recurrent treatments much smaller in this approach than in the one utilizing BACE activators as iAβ and C99 degradation agents.

## 20. Therapeutic Options for the Prevention and Treatment of Both Conventional and Unconventional Forms of AD Based on Direct Interference with the Mechanism Underlying AβPP-Independent Production of C99

All therapeutic strategies for the prevention and treatment of AD, in both its conventional and unconventional forms, have one common goal, namely, to either prevent or abrogate production of C99 in the AβPP-independent pathway. In one approach, considered above, this is achieved through the inhibition of the neuronal integrated stress response. Generation of C99 in the AβPP-independent pathway depends vitally on the neuronal ISR. Indeed, the ISR state enables production of components essential for this pathway. When the ISR state is reversed, the supply of these components is abrogated, and the AβPP-independent C99 generation pathway is disabled. For their full effect in the prevention and treatment of AD, ISR inhibitors have to be administered long-term. This could be possible with ISR inhibitors specifically targeting only the AD-affected neurons. Such inhibitors still need to be developed. Currently available systemic ISR inhibitors cannot be administered long-term due to their deleterious effect in such an application, and their transient implementation would provide only temporary relief lasting no longer than the duration of the treatment.

Another approach to the prevention and treatment of AD, considered above, is the depletion of iAβ and C99. In the conventional form of the disease, this approach is anticipated to be highly efficient. Mechanistically, it works by either preventing the elicitation of, or reversing, the neuronal ISR. In the prevention of conventional AD, transiently activated BACE1 and/or BACE2 or other iAβ/C99 degradation agents, administered transiently, deplete iAβ. Its levels do not reach the T1 threshold, the neuronal ISR is not elicited, the AβPP-independent C99 production pathway is not activated, and the disease does not occur within the remaining lifetime of the treated individual. In the treatment of conventional AD, transiently activated BACE1 and/or BACE2 or other transiently administered iAβ/C99 degradation agents deplete iAβ and C99. In this approach, C99 is depleted with the assistance of the concurrent transient inhibition of ISR, which abrogates its influx for the duration of the treatment. Following the transient treatment, the ISR state is reversed because levels of both iAβ and C99 are below the T1 threshold, the AβPP-independent C99 production pathway is disabled, and the disease does not recur within the remaining lifetime of the treated individual. On the other hand, in unconventional AD, a single C99 depletion treatment, assisted by transiently administered ISR inhibitors, is not sufficient to provide long-term relief from the disease, but, when recurrently repeated, such treatment can accomplish the goal of maintaining the individual at a disease-free state.

Thus, the targets of the therapeutic strategies discussed above are removed from the actual process that these strategies attempt to affect. Indeed, the suppression of the neuronal ISR is once removed from the AβPP-independent production of C99, which occurs downstream from elicitation of the ISR state, whereas the depletion of iAβ and/or C99 is twice removed (it affects the neuronal ISR, which, in turn, impacts production of C99 in the AβPP-independent pathway). In this context, it makes perfect sense to attempt a direct mechanistic interference with the production of C99 in the AβPP-independent pathway. The following sections consider potential molecular mechanisms underlying the AβPP-independent production of C99 and conclude that the most plausible one is RNA-dependent amplification of the 3′-terminal segment of human AβPP mRNA encoding C99. Systemically targeting RNA-dependent mRNA amplification would be problematic. This is a major physiological mechanism, which is pivotal in processes such as erythroid differentiation [172,173] and the deposition of extracellular matrix [174]. Any systemic interference with this process in general is bound to have significant detrimental consequences.

Therefore, only the asymmetric amplification of AβPP mRNA can be targeted, in a specific manner, in an attempt to suppress the AβPP-independent production of C99 in AD. To the extent of our understanding, RNA-dependent amplification of any specific mRNA has two principal requirements. One is sustained elicitation of the integrated stress response, which supplies the essential components of the RNA-dependent mRNA amplification pathway. As discussed above, currently, the ISR can be inhibited only systemically. Development of ISR inhibitors specifically targeting the neurons or, even better, only the AD-affected neurons, would provide an effective means to suppress the AβPP-independent production of C99. Another requirement for the activity of the RNA-dependent mRNA amplification mechanism is the eligibility of mRNA species for the amplification process. Specific requisites that make an mRNA species eligible for amplification are well understood [172,173]. Human AβPP mRNA is an eligible template (i.e., “mRNA progenitor”) for RNA-dependent mRNA amplification. Therefore, any interference with the features of AβPP mRNA that make it eligible for amplification would potentially constitute an effective and highly specific AD therapy. Specific approaches for such interference are discussed below. If successful, these approaches would render AβPP mRNA ineligible for amplification and would suppress production of C99 in the AβPP-independent pathway. The anticipated outcomes of these strategies in the prevention and treatment of AD are conceptually identical to those illustrated in Figure 5 and Figure 6 for the conventional disease and in Figure 7 and Figure 8 for unconventional AD. In these Figures, the AβPP-independent production of C99 is suppressed via inhibition of the ISR, one of two principal requirements for the activity of the RNA-dependent mRNA amplification process. Depriving AβPP mRNA of its amplification eligibility, the second principal requirement for activity of the RNA-dependent mRNA amplification process, would yield exactly the same effect.

## 21. Putative Mechanisms of AβPP-Independent Generation of C99

### 21.1. In All Putative Mechanisms of AβPP-Independent Production of C99, Translation Initiates from the AUG Encoding Met671 of AβPP

In 1987, significant progress was made in understanding the molecular basis of Alzheimer’s disease. This year is when several research groups, almost concurrently, succeeded in cloning and sequencing human AβPP mRNA [231,232,233]. It transpired that human AβPP occurs in several size variants, which reflect alternative splicing of its mRNA. The present discussion considers its variant that is 770 amino acids long. Following its formation, AβPP770 is cleaved by beta-secretase between amino acids 671 and 672. This generates the C-terminal fragment of AβPP. It starts at the N-end with amino acid residue 672 and terminates at the C-end with amino acid residue 770; it contains 99 amino acids and, therefore, is referred to as C99.

The same year, 1987, when the nucleotide sequence of human cDNA became available, two investigators, Breimer and Denny, noticed that the amino acid in position 671 of human AβPP is, in fact, methionine [234]. This opened up a possibility that the C99 fragment of human AβPP can, in principle, be generated not only by the beta cleavage of its precursor but also by the initiation of translation of AβPP mRNA from the AUG encoding Met671 of AβPP. This notion was supported by the observation that the AUG codon encoding Met671 of human AβPP is situated within the optimal translation initiation nucleotide sequence known as the Kozak motif [234]. Moreover, the rarity of such positioning of AUG codon was emphasized by a further observation that human AβPP mRNA contains twenty in-frame AUG codons, but of those, only one, the AUG encoding Met671, is situated within the optimal translation initiation nucleotide sequence; not even the AUG encoding of the conventional translation-initiating methionine is positioned within the optimal translation initiation nucleotide sequence. On the basis of these observations, Breimer and Denny proposed that the position of the AUG encoding Met671 of human AβPP is not random, but reflects its physiological function, and that in AD, translation of AβPP initiates not only conventionally, but also unconventionally, from the AUG encoding Met 671 of AβPP. Breimer and Denny reasoned that in such a case, the N-terminal methionine would be cleaved-off co-translationally by N-terminal methionine aminopeptidase (MAP), and thus C99 and, subsequently, Aβ would be produced independently of AβPP [234]. As discussed below, this attribute of human AβPP mRNA (or of its derivatives), namely, the potential to initiate translation from the AUG encoding Met671 of AβPP, forms the basis for all potential mechanisms of the AβPP-independent production of C99.

### 21.2. The Primary Product of Translation Initiated from the AUG Encoding Met671 of AβPP Is C100: It Is Converted into C99 Post-Translationally

At the time when Breimer and Denny forwarded their proposal (see the preceding sub-section), it was assumed that the translation-initiating methionine is always removed co-translationally by MAP and is never retained in the primary translation product. If this were the case, the primary product of translation initiated at the AUG encoding Met671 of human AβPP would terminate with aspartate in position 672 at its N-end, and thus be identical to, and indistinguishable from, C99 generated by the beta-cleavage of AβPP between amino acids 671 and 672. This, however, is not the case. It transpired that MAP is not always capable of removing the N-terminal translation-initiating methionine. Indeed, for MAP to perform the cleavage, both the translation-initiating methionine and the next downstream amino acid residue should be accommodated within the MAP’s active site. Due to topological constraints, only a few amino acids under a certain threshold size can be accommodated together with methionine in the active site of methionine aminopeptidase. These amino acids are the following: Gly, Ser, Cys, Thr, Pro, and Val (listed in order of increasing size). Any amino acid larger than valine would not fit together with the methionine in the MAP’s active site, and the translation-initiating methionine would be retained in the primary translation product [235,236,237,238,239,240].

As mentioned above, in human AβPP, Met671 is followed by aspartate in position 672. Aspartate is much larger than valine and would not fit together with methionine into the MAP’s active site. Therefore, if translation of human AβPP mRNA were to initiate at the AUG codon encoding Met671, the translation-initiating methionine would not be removed co-translationally by MAP and would be retained in the primary translation product. In such a case, the primary translation product would be C100 (i.e., N-terminal Met-C99), readily distinguishable from C99 generated by the beta-cleavage of AβPP. The N-terminal translation-initiating methionine would not be retained for long but would be removed by one of numerous aminopeptidases with broad specificity [239,240]. Such a removal, however, would occur post- rather than co-translationally, and the pool of C100 would occur within the living cell. It should be noted that C100 was not and could not be seen in postmortem samples from AD patients. This is because in dying cells, protein synthesis ceases well before proteolysis stops. Therefore, with no influx of C100 and with still functioning aminopeptidases, the entire pool of C100 would be converted into C99. On the other hand, C100 pools should be detectable in living AD-affected neurons and in the adequate AD model systems (see below).

### 21.3. Putative Mechanisms of AβPP-Independent Production of C99

As was mentioned above, Breimer and Danny proposed that in AD, translation of the intact AβPP mRNA is initiated internally, from the AUG normally encoding Met671 of AβPP [234]. This proposal was tested experimentally in two studies. The rationale in both was that if the proposed internal initiation of translation of AβPP mRNA does occur, then alterations of mRNA upstream from the AUG encoding Met671 would abrogate the conventional translation of AβPP mRNA but would not affect that initiated internally. Therefore, the reasoning goes, if, following these alterations, the production of Aβ persists, it would indicate the plausibility of the internal initiation of translation of C99. On the other hand, if, following the alterations, the production of Aβ ceases, this would rule out the internal initiation of translation of C99.

In one study testing Breimer and Danny’s proposal, frame-shifting mutations were introduced upstream from the AUG encoding Met671 of AβPP [241]. In another study, a translational stop codon was inserted, also well upstream of the AUG encoding Met671 of AβPP [242]. In both studies, the production of Aβ from altered DNA ceased. Both studies, therefore, ruled out the internal initiation of translation of C99. In our opinion, the AβPP-independent production of C99 is highly unlikely to occur via the internal initiation of translation of AβPP mRNA. However, the conclusions of the studies referenced above [241,242] are patently invalid. Breimer and Denny postulated the AβPP-independent production of iAβ only in the AD-affected, but not in the healthy, neurons. Both studies in question, however, were carried out not in the neuronal cells, and certainly not under AD conditions, and, therefore, their conclusions should be discounted; the occurrence of the internal initiation of translation of AβPP mRNA remains a valid (albeit unlikely) notion, which should be tested in an appropriate experimental model.

On the other hand, there are three additional potential mechanisms of the AβPP-independent production of C99 via its translation from the AUG encoding Met671 of AβPP. In all three mechanisms, translation of C99 occurs conventionally, rather than via internal initiation, from 5′-truncated AβPP mRNA molecules where the AUG encoding Met671 of AβPP is the first, 5′-most translation initiation codon. Principally, therefore, these mechanisms are distinguished from each other mostly in the ways leading to 5′-truncations of AβPP mRNA. One such potential mechanism operates via the internal initiation of transcription of the intact AβPP mRNA deep within its coding region. This mechanism would generate capped 5′-truncated AβPP mRNA, and its conventional translation would produce C100, subsequently converted into C99. In another potential mechanism, the appropriately 5′-truncated AβPP mRNA molecule is generated via site-specific cleavage of AβPP mRNA by a specialized nuclease. This mechanism would generate uncapped C100-encoding mRNA, which would be translatable under ISR conditions. 

The third potential, ISR-enabled mechanism generating severely 5′-truncated, C100-encoding mRNA is the most plausible one. Of all potential mechanisms underlying AβPP-independent production of C99, it is the only one supported by the empirical data. It operates via asymmetrical RNA-dependent amplification of only the 3′-terminal portion of human AβPP mRNA. Human AβPP mRNA is uniquely eligible to serve as the initial template in the amplification process, and the interference with this eligibility forms the foundation for RNA-based therapeutic approaches discussed below. First, however, in the following sections, we briefly summarize the process of mammalian RNA-dependent mRNA amplification, present evidence of the eligibility of human AβPP mRNA for this process, and define features that make human AβPP mRNA an eligible template of the RNA-dependent amplification mechanism and thus constitute the interference targets.

## 22. Principles of Mammalian RNA-Dependent mRNA Amplification: The Chimeric Pathway

**Figure 22:**
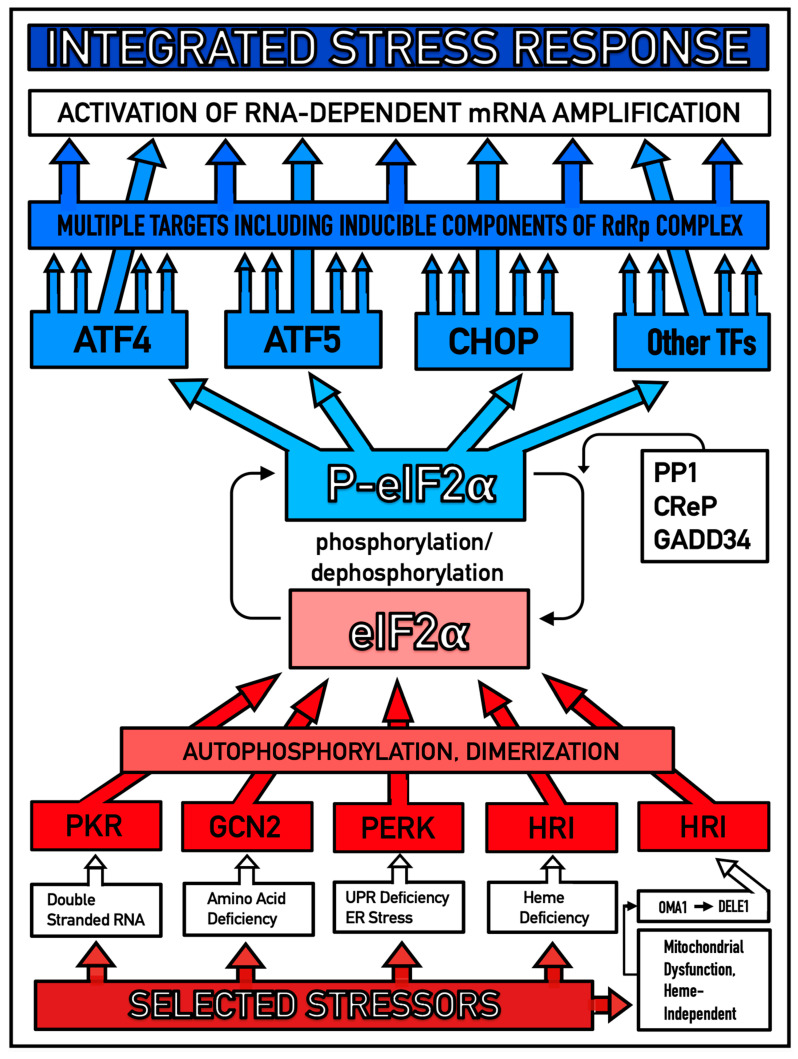
**Role of integrated stress response in induction of mammalian RNA-dependent mRNA amplification: ISR enables production of essential components not present under regular conditions**. The “integer” of the integrated stress response is phosphorylation of eukaryotic translation initiation factor 2alpha (eIF2α) at its amino acid residue Ser51. It is enacted by members of the family of eIF2α kinases, which comprises protein kinase double-stranded RNA-dependent (PKR), PKR-like endoplasmic reticulum kinase (PERK), general control non-derepressible-2 kinase (GCN2), and heme-regulated inhibitor kinase (HRI), with each kinase responding to a defined set of stressors. All eIF2α kinases exhibit significant homology in their catalytic domains but contain distinctly defined regulatory domains; this is why they respond in a similar manner (i.e., auto-phosphorylation and dimerization) and with identical results (i.e., phosphorylation of eIF2α) to distinct sets of stressors. When eIF2α is phosphorylated, ISR ensues. Under the ISR state, global cellular protein synthesis is severely suppressed. Concurrently, production of a subset of cellular proteins, including a number of transcription factors, is activated. Some of these transcription factors and/or products of genes activated by them constitute components that are essential for the operation of the RNA-dependent mRNA amplification mechanism, comprising, presumably, the RNA-dependent RNA polymerase (RdRp) complex and the helicase/RNA cleaving activity complex.

In the mammalian RNA-dependent mRNA amplification process [12,172,173,174,241,242,243,244,245,246,247,248], every eligible mRNA molecule can be repeatedly utilized as a template to produce, after a few intermediate steps, numerous additional mRNA molecules. Such amplification-eligible conventionally genome-transcribed mRNA molecules are referred to as mRNA progenitors (amplification eligibility requirements are discussed below). mRNA progenitors can occur in the cell in hundreds or even thousands of copies, each repeatedly employed in the production of new mRNA molecules. This process, therefore, is extraordinarily powerful and is comparable to a massive gene amplification, with every copy of the mRNA progenitor equivalent to a gene copy. The eligibility of a particular mRNA species is one of the two principal requirements for the occurrence of RNA-dependent mRNA amplification. Another principal requirement is the sustained neuronal ISR state. This is because, as discussed above, the ISR state enables the production of components that are essential for the operation of the RNA-dependent mRNA amplification mechanism, comprising, presumably, the RNA-dependent RNA polymerase (RdRp) complex and the helicase/RNA cleaving activity complex (see below). General principles of the induction of mammalian RNA-dependent mRNA amplification by sustained ISR are outlined schematically in Figure 22.

**Figure 23:**
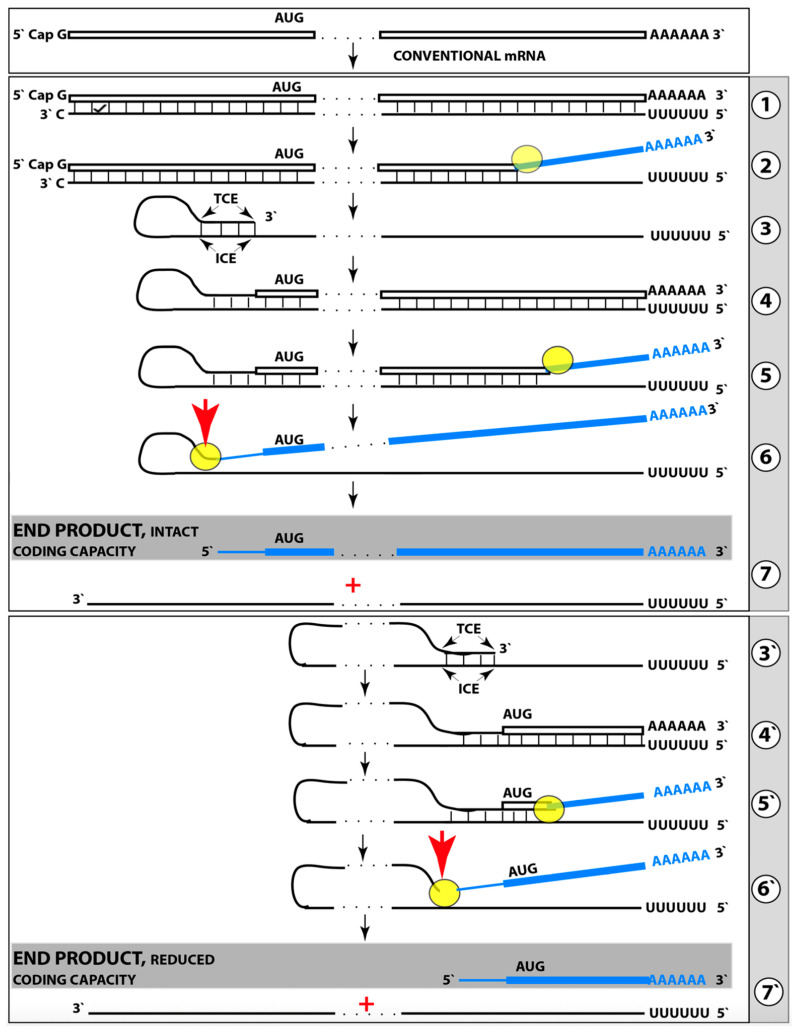
**Principles of mammalian RNA-dependent mRNA amplification: The chimeric pathway**. *Single black lines*: Antisense RNA. *Boxed black lines*: Sense RNA strands. *Wide blue lines*: Sense RNA strands separated from their antisense RNA counterparts by helicase activity. *Single blue lines*: TCE segment of the antisense RNA separated by helicase activity from the complementary ICE segment of the antisense RNA. *Yellow discs*: Enzymatic complex comprising helicase activity, nucleotide-modifying activity, and single-stranded RNA cleaving activity. *Red arrows*: Sites of cleavage by the RNA cleaving activity of the helicase complex. Cleavage occurs when the helicase complex reaches the single-stranded portion of the hairpin-like intermediate of RNA-dependent mRNA amplification. *AUG*: Translation initiation codon of the mRNA molecule. *TCE*: Strictly 3′-terminal complementary element of the antisense RNA. *ICE*: The internal complementary element of the antisense RNA. The TCE and ICE must be sufficiently complementary to form a double-stranded self-priming structure. *Top panel*: Gene-transcribed amplification-eligible mRNA molecule designated as “mRNA progenitor”. *Middle panel*: Stages **1** through **7** of the chimeric RNA-dependent mRNA amplification pathway with the ICE positioned within the segment of the antisense RNA strand corresponding to the 5′UTR of the progenitor mRNA. *Bottom panel*: Defining stages **3′** through **7′** (corresponding to stages 3 through 7) of the chimeric RNA-dependent mRNA amplification pathway with the ICE positioned within a segment of the antisense RNA strand corresponding to the protein-coding region of the progenitor mRNA. (**1**): Antisense RNA is transcribed from the progenitor mRNA template by RNA-dependent RNA polymerase (RdRp). Transcription initiates within the 3′-terminal poly(A) region of the template RNA and terminates with “C” transcribed from the 5′-terminal cap-G. (**2**): Newly transcribed antisense RNA is separated from its mRNA template by the helicase activity. Helicase enzymatic complex mounts the 3′-terminal poly(A) portion of the double-stranded RNA and proceeds along the sense RNA, modifying about every fifth nucleotide. These modifications preclude nucleic acid hybridization and prevent strands from re-annealing. Importantly, the antisense RNA remains unmodified. (**3**): Mutually accessible TCE and ICE complementary elements form a self-priming double-stranded structure. (**4**): The 3′ terminus of self-primed antisense RNA is extended by RdRp, resulting in a hairpin-like chimeric molecule comprising both sense and antisense RNA components and dubbed “chimeric RNA intermediate”. (**5**): Complementary segments of the chimeric RNA intermediate are separated by helicase activity. Helicase complex mounts the 3′-terminal poly(A) portion of the chimeric RNA intermediate and proceeds along the sense RNA, introducing frequent nucleotide modifications. (**6**): Upon reaching single-stranded portion of the chimeric RNA intermediate, cleaving activity of the helicase complex cleaves the RNA molecule. (**7**): Two end products of the chimeric RNA-dependent mRNA amplification pathway. One is the antisense RNA truncated at its 3′ end; it loses either the entire TCE or a 3′ portion of the TCE. The other is the chimeric RNA molecule. It comprises the sense RNA component attached at the 5′ end to a 3′-terminal fragment of the antisense RNA (hence, chimeric). The antisense RNA component of the chimeric RNA end product is either the entire TCE or a 3′ portion of the TCE cleaved-off following separation of strands of the chimeric intermediate. The chimeric end product is 5′-truncated (in comparison with the mRNA progenitor) but contains the entire protein-coding region of the mRNA progenitor. It is also uncapped and heavily modified; these modifications potentially facilitate its translation under ISR conditions. Stages **3′** through **7′** are conceptually identical to stages 3 through 7. The only difference is that the ICE element is positioned within the segment of the antisense RNA corresponding to the protein-coding region of the progenitor mRNA. Consequently, the 5′-truncated chimeric RNA end product contains only a 3′ portion of the coding region of the progenitor mRNA and thus encodes only the C-terminal fragment of the progenitor mRNA-encoded polypeptide.

In every amplification cycle, which is illustrated schematically in Figure 23, the mRNA progenitor molecule serves as a template for RNA-dependent RNA polymerase (RdRp) to produce its antisense RNA complement. Synthesis of the antisense RNA initiates within the poly(A) segment of the mRNA progenitor. Therefore, it contains the poly(U) segment at its 5′ end and terminates with the “C”, a copy of the cap-“G”, at its 3′ end. The antisense RNA is separated from its template by a helicase activity. It mounts the 3′ terminal poly(A) segment of the mRNA template, which is in the double-stranded structure with the poly(U) of the antisense RNA, and proceeds along the mRNA template, separating sense and antisense RNA strands. In the process, it also modifies, on average, every fifth nucleotide of the sense RNA molecule. These modifications preclude nucleic acid hybridization and therefore prevent the re-annealing of the separated RNA strands; as discussed below, they also, presumably, enable mRNA translation under ISR conditions. At the completion of this stage of amplification, the progenitor mRNA can serve again as the amplification template. Importantly (for reasons explained below), the separated antisense strand remains unmodified.

This is where the amplification eligibility requirements for an mRNA species come into play. For the RdRp-mediated production of a new mRNA molecule from the antisense RNA template, the latter must fold into a self-priming configuration. This means that the 3′-terminal segment of the antisense RNA must have an internal complementary counterpart within the antisense RNA molecule. Importantly, since the antisense RNA remains unmodified following its separation from the progenitor mRNA molecule, intramolecular complementary interactions can occur. The complementarity does not have to be complete, just sufficient to form a stable self-priming structure. The 3′-terminal complementary element of the antisense RNA is referred to as the TCE, and its internal complementary element counterpart as the ICE. One of the amplification eligibility requirements is the occurrence in the antisense RNA of the TCE and ICE elements. Another is their mutual accessibility within the folded antisense RNA configuration. The second eligibility requirement appears weak, but, in fact, it could be stronger than the first one. Folded unmodified RNA forms a very dense structure supported by intramolecular complementary interactions, where two random segments are highly unlikely to be precisely aligned. As discussed below, this is especially relevant for AβPP RNA, where the TCE and ICE elements are separated by over two thousand nucleotides. The amplification eligibility requirements for an mRNA species are, of course, in addition to the prerequisite that the cell must be in the sustained ISR state, which enables the production of components presumably essential for the formation and functioning of the RdRp complex.

Following the formation of self-priming structure, the 3′ terminus of the antisense RNA, in fact, its TCE element, is extended by RdRp. The 5′-terminal portion of the antisense strand, which remains single-stranded following the formation of the self-primed structure, is utilized as a template in this transcription process. Such an extension generates the sense RNA strand terminating with the 3′ poly(A) segment transcribed from the 5′-terminal poly(U) of the antisense RNA. The newly generated sense RNA, however, is not a full-size copy of the progenitor mRNA but is truncated in the 5′ portion. This is because, due to the internal position of the ICE element, synthesis of the sense RNA always starts from an internal portion of the self-primed antisense RNA. Thus, the extension of the self-primed antisense RNA yields 5′-truncated sense RNA covalently attached to the 3′ terminus of the antisense RNA, together forming a hairpin-like structure. This structure contains both sense and antisense components and is, therefore, chimeric. This hairpin-like molecule is an intermediate in the amplification process and is referred to as a chimeric intermediate. The junction between the sense and antisense components of the chimeric intermediate marks the starting point of the extension of self-primed antisense RNA and has been named the chimeric junction. Accordingly, this RNA-dependent mRNA amplification pathway has been designated chimeric.

The next stage of the RNA-dependent mRNA amplification process is enacted by the helicase or helicase complex invoked above. Its function at this stage is principally the same as in the separation of the antisense RNA from its mRNA progenitor template. It recognizes and mounts the 3′-terminal poly(A) of the sense RNA component of the chimeric intermediate, which is in double-stranded configuration with the 5′-terminal poly(U) of the antisense RNA component, and proceeds along it in the 5′ direction. It not only separates RNA strands but also introduces frequent modifications, as described above. When it reaches the single-stranded portion of the chimeric intermediate, it cleaves either at the 5′ end of the TCE element or within the TCE element. The former occurs in the unlikely case where the TCE and ICE are fully complementary, and the TCE/ICE double-stranded structure contains no mismatches; such cleavage completes the chimeric amplification process. The latter takes place when the TCE/ICE structure contains mismatches; it occurs at the first encountered mismatch. What follows depends on the interaction of the remaining 5′ portion of the TCE with its ICE counterpart. If it is unstable, this is the end of the chimeric amplification cycle. If, on the other hand, it is stable, it can prime another extension. In such a case, the chimeric junction would move in the 5′ direction from the initial one, a process referred to as the chimeric junction shift [11,12,173]. If the TCE/ICE complex contains multiple mismatches, the above scenario can recur repeatedly until, following the cleavage, neither stable self-priming structure can form nor its extension can occur. It follows that, depending on the number of self-primed extension events, a single amplification cycle can result in multiple chimeric RNA end products.

Thus, the chimeric RNA-dependent mRNA amplification cycle yields two types of the end product. One is the antisense RNA truncated at its 3′ end. Indeed, following the cleavage of the chimeric intermediate, the antisense RNA molecule loses either the entire TCE or a 3′ portion of the TCE. Only one such molecule is produced in a single amplification cycle. The other type of end product is the chimeric RNA molecule. It comprises the sense RNA component attached at the 5′ end to a 3′-terminal fragment of the antisense RNA (hence chimeric). The antisense RNA component of the chimeric RNA end product is either the entire TCE or a 3′ portion of the TCE cleaved-off following separation of strands of the chimeric RNA intermediate. A single amplification cycle can produce multiple chimeric end products; their number is always equal to that of the self-primed extension events discussed above. The chimeric RNA end product is not only 5′-truncated (in comparison with the mRNA progenitor) but also uncapped and heavily modified; these modifications potentially facilitate its translation under ISR conditions [249]. Please note that in a scenario depicted in the middle panel of Figure 23, the ICE is located within the portion of the antisense RNA corresponding to the 5′ untranslated region (5′UTR) of the progenitor mRNA. Therefore, although the sense RNA component of the chimeric intermediate is 5′-truncated, it contains the intact coding region of the progenitor mRNA since the truncation occurs within the 5′UTR.

It should be mentioned that the chimeric RNA-dependent mRNA amplification cycle can morph into a distinctly different type of RNA amplification, namely the polymerase chain reaction process. In this process, observed in the erythroid system [173], the antisense component of the chimeric intermediate is polyadenylated at its 3′ end in conjunction with the cleavage of the chimeric RNA intermediate following separation of the RNA strands. This generates a molecule containing the poly(A) segment at its 3′ end and the poly(U) segment at its 5′ terminus. The presence of 3′-terminal poly(A) makes it a legitimate template for RdRp, and the product of the RdRp-mediated transcription contains the poly(A) segment at its 3′ end again, thus enabling another round of the RdRp-mediated transcription, a typical PCR process. The PCR-like RNA-dependent mRNA amplification process is, however, outside of the scope of the present Perspective; its more detailed description can be found in [11,12,173].

## 23. Asymmetric RNA-Dependent mRNA Amplification Generates 5′-Truncated mRNA Encoding Only the C-Terminal Fragment of the Original Polypeptide

If the chimeric RNA-dependent mRNA amplification mechanism were capable only of producing amplified RNA containing the entire coding region of its mRNA progenitor, such a mechanism would be of little interest in its application to AD in the ACH2.0 paradigm. Indeed, in the framework of the ACH2.0, any potential candidate mechanism enacting the AβPP-independent generation of C99 must be able to produce AβPP mRNA 5′-truncated so that the AUG codon encoding Met671 of AβPP becomes the first functional translation initiation codon. The question, therefore, is as follows: Can the chimeric RNA-dependent AβPP mRNA amplification process accomplish this (provided it occurs in the first place)? And the answer is: Most certainly. To elaborate this answer, the present section analyzes the potential of the RNA-dependent mRNA amplification mechanism to generate the chimeric end product RNA truncated within the coding region of the mRNA progenitor, encoding only the C-terminal fragment of the original polypeptide, whereas the following section examines whether such a mechanism is applicable to human AβPP mRNA.

In the scenario considered in the preceding section, the RNA end product of the chimeric amplification cycle contains the entire coding region of the mRNA progenitor because the ICE element is located in the portion of the antisense RNA corresponding to the 5′UTR of its sense counterpart. In such a case, the 3′ extension of the self-primed antisense RNA generates sense RNA starting with the 3′-terminal segment of the 5′UTR. Consequently, the chimeric RNA end product comprises the TCE or a portion thereof, the 3′-terminal segment of the 5′UTR, and the intact coding region followed by the 3′UTR and the 3′-terminal poly(A) segment.

While the TCE is, by definition, 3′-terminal, the ICE can be positioned anywhere within the antisense RNA molecule. Its position defines the extent of the 5′ truncation in the sense RNA strand generated by the extension of the TCE element. One of the possibilities is that the ICE is situated within the portion of the antisense RNA corresponding to the coding region of the mRNA progenitor. In this case, extension of the TCE element would produce the sense RNA strand truncated within the coding region. Following helicase-mediated strand separation and cleavage of the chimeric RNA intermediate, the newly formed chimeric RNA end product of amplification would comprise the TCE or a portion thereof, followed by the 3-terminal segment of the coding region, the 3′UTR, and the 3′-terminal poly(A) segment. This RNA potentially encodes the C-terminal fragment of the progenitor-encoded polypeptide. However, such a translational outcome requires the presence of the in-frame AUG (or another functional translation initiation codon) within the 5′-truncated portion of the coding region of the chimeric RNA end product. In this scenario, the RNA-dependent mRNA amplification process not only generates the RNA molecule missing the 5′-terminal portion of the mRNA progenitor, but its translation produces only the CTF of the progenitor-encoded polypeptide. Therefore, this scenario, defined by the occurrence of the ICE within the portion of the antisense RNA corresponding to the coding region of the mRNA progenitor, is referred to as the asymmetric RNA-dependent mRNA amplification process; it is schematically depicted in the bottom panel of Figure 23.

Thus, scenarios described in the preceding and the present sections yield conceptually distinct types of the chimeric mRNA amplification product. The former produces an RNA molecule retaining the entire protein-coding capacity of the mRNA progenitor, whereas the latter generates the chimeric RNA end product containing only the 3′-terminal portion of the protein-coding region and, upon translation, yields only the C-terminal fragment of the polypeptide encoded by the mRNA progenitor, provided certain conditions are met. Interestingly, and in sharp contrast, 3′-truncated antisense RNA molecules generated in both scenarios are of conceptually identical types. In both, only the TCE element, or a portion of it, is cleaved off, and sense-orientation complements of both would retain the entire protein-coding region of the mRNA progenitor. Therefore, if the antisense RNA end products, produced in the either “regular” or asymmetric chimeric amplification cycle, are polyadenylated at the 3′ end and utilized as the initial amplification template in the PCR amplification process, sense-orientation RNA containing the entire coding region of the mRNA progenitor, and encoding the entire polypeptide, would be generated in both cases [11,12,173]. It follows that if AβPP mRNA is amplified in the AD-affected neurons in both chimeric and PCR cycles, amplification would be asymmetric and only C99 would be produced in the former, but the entire AβPP would be produced in the latter at the same time.

## 24. Human AβPP mRNA Is an Eligible Template for RNA-Dependent Amplification Yielding 5′ Truncated AβPP mRNA Encoding the C100 Fragment of AβPP

### 24.1. How to Test the Eligibility of an mRNA Species for the RNA-Dependent Amplification Process?

It follows from the preceding sections that in order to qualify as the eligible RNA-dependent mRNA amplification template, an RNA species must meet two prerequisites. First, its antisense RNA counterpart must contain two segments that are sufficiently complementary to form a stable double-stranded structure; one of those must be strictly 3′-terminal. Second, these two complementary segments, the TCE and ICE, must be mutually accessible within the folded configuration of the antisense RNA molecule. Whether human AβPP mRNA, and, actually, any other 3′-terminal poly(A)-containing mRNA species, meets these two prerequisites can, in fact, be assessed in a model experiment. The principal components of such an experiment are as follows: (1) mRNA species of interest; (2) an enzyme capable of transcribing the antisense strand from the mRNA of interest (standing for RdRp); and (3) an activity capable of separating the newly produced antisense strand from its mRNA template (standing for helicase employed in the RNA-dependent mRNA amplification process). The first component is obvious: purified mRNA of interest or even a mixture, such as total poly(A)-containing RNA, which includes the mRNA species of interest. The second component is modeled by RNA-dependent DNA polymerase, RdDp (reverse transcriptase); the resulting cDNA is the antisense strand. The third component, the strand-separating activity, is modeled by RNAse H. All viral preparations of RdDp contain RNAse H activity. If, however, cloned RdDp is utilized in such an experiment, RNAse H activity must be added to the reaction. The reaction mix also contains oligo dT primer as well as dNTPs.

In this experiment, oligo dT hybridizes with the 3′-terminal poly(A) of mRNA and acts as the primer, which is extended by RdDp. RNAse H activity, which targets RNA molecules that are in complex with DNA, degrades the mRNA template and thus releases the antisense product. If the newly synthesized antisense strand (cDNA) contains the TCE and ICE elements, and if these elements are mutually accessible within the folded configuration of the antisense strand, a self-primed structure would be formed, and its 3′ end (the TCE) would be extended by RdDp. Size analysis of the resulting cDNA would indicate whether or not the extension occurred, and nucleotide sequencing would ascertain the occurrence of the extension, determine the extension start site, and define the TCE and ICE elements.

### 24.2. Human AβPP mRNA Is Eligible for the Asymmetric RNA-Dependent Amplification: The Resulting 5′-Truncated AβPP mRNA Encodes the C100 Fragment of AβPP

The assessment of the eligibility of human AβPP mRNA for the RNA-dependent mRNA amplification process, described in principal terms in the preceding sub-section, was, in fact, carried out, albeit inadvertently. As was mentioned above, in 1987, three research groups published, almost simultaneously, the nucleotide sequence of human AβPP cDNA [231,232,233]. The next year, another research group, namely Mita and co-workers, published their nucleotide sequence of human AβPP cDNA that was identical to those published previously in their common portion, but was significantly extended at the 3′ terminus [250]. In their paper, Mita and co-workers proposed that the 3′-extended human AβPP cDNA originated from an mRNA molecule whose transcription was initiated well upstream from the transcription start site (TSS) deduced previously in [231,232,233]. At the time of Mita group’s publication, the nucleotide sequence upstream from the TSS deduced previously was not yet known. However, shortly after Mita group’s findings, this nucleotide sequence was determined and published [251]. At that time, it became evident that the 3′ extension of human AβPP cDNA seen by Mita and co-workers could not have originated from AβPP mRNA transcribed from the TSS well upstream from the previously established one [251]. As a result, Mita and co-workers declared their results an artifact and corrected their paper accordingly [250].

**Figure 24:**
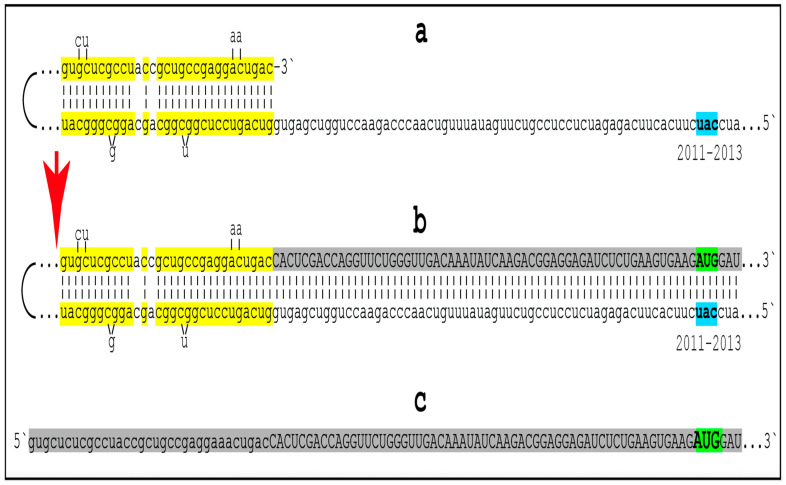
**Human AβPP mRNA is eligible for the asymmetric RNA-dependent amplification process; the resulting 5′-truncated AβPP mRNA encodes the C99 fragment of AβPP**. *Lowercase letters*: Nucleotide sequences of the relevant portions of human antisense AβPP RNA. *Uppercase letters*: Nucleotide sequence of 5′-truncated human AβPP mRNA generated by the extension of the 3′ terminus of self-primed antisense AβPP RNA. Numbers 2011–2013 refer to positions on the antisense RNA counted as the number of nucleotides from the 3′ terminus of the human AβPP antisense RNA. These nucleotides (uac), highlighted in blue, are relevant to the present discourse because they are complements of the nucleotides (AUG), highlighted in green, that form the codon encoding Met671 of human AβPP. (**a**): The antisense molecule is shown in self-primed configuration. The TCE and ICE complementary elements of the antisense RNA molecule, which guide the formation of a self-priming structure, are highlighted in yellow. (**b**): Extension of the 3′ terminus of the human self-primed antisense AβPP RNA generates a severely 5′-truncated AβPP mRNA molecule (highlighted in gray), which constitutes the sense-orientation component of the chimeric sense/antisense RNA intermediate. Complementary segments of this intermediate are separated by helicase activity, and the molecule is cleaved (red arrow) when the helicase complex reaches the single-stranded portion of the intermediate. (**c**): Cleavage of the chimeric RNA intermediate yields the chimeric RNA end product. It comprises the 3′-terminal portion of human AβPP mRNA and the TCE element (or its portion) of the antisense RNA, with the latter forming the 5′ end of the chimeric RNA end product. Translation of the chimeric RNA end product would initiate from the AUG encoding Met671 of AβPP and result in the C100 fragment, subsequently processed into C99.

However, careful consideration of the results obtained by Mita and co-workers in light of the present discourse strongly indicates that these results were not an artifact but reflected the physiological process potentially occurring in AD. It reveals that the 3′ extension of human AβPP cDNA seen by Mita’s group is neither random nor unrelated to AβPP, but is, rather, a segment of the sense AβPP DNA that could have been generated only by the extension of the self-primed AβPP antisense strand exactly as described above. The analysis of the nucleotide sequence of the 3′-extended human AβPP cDNA indeed confirmed the occurrence of a self-primed extension, determined the extension start site (the junction between the antisense- and sense-orientation portions of the extended cDNA), and defined the TCE and ICE elements [252,253,254]. The deduced process of self-primed extension of the human antisense AβPP RNA strand separated from its template is illustrated in Figure 24. The antisense RNA strands contain the TCE and ICE elements highlighted in yellow in Figure 24. These elements are separated by more than two thousand nucleotides, and the ICE element is situated deep within the portion of the antisense strand corresponding to the coding region of human AβPP mRNA. Nevertheless, the TCE and ICE are mutually accessible in the folded configuration of the antisense strand and form a self-priming structure, which is extended into the sense-orientation strand, thus generating the chimeric RNA intermediate. Upon completion of the extension, complementary strands are separated by the helicase complex. When the helicase reaches the single-stranded portion of the chimeric intermediate, it, or an activity associated with it, cleaves the RNA molecule, thus producing the chimeric RNA end product. As described above, the crucial feature determining the outcome of translation of this RNA end product is the location of the first functional translation initiation codon. It is located 58 nucleotides downstream from the chimeric junction, and it is, in fact, the AUG encoding Met671 of AβPP. Thus, the results obtained by Mita and co-workers [250] establish not only that human AβPP mRNA is eligible for the RNA-dependent amplification process but also that chimeric mRNA generated in this process can be translated into the C100 fragment of AβPP.

It could be legitimately asked by the reader why the 3′ extension of human AβPP cDNA occurred in experiments carried out by Mita and co-workers [250], but was not seen in the preceding experiments by the other three research groups [231,232,233]. The answer is trivial and technical. The three research groups that first sequenced human AβPP cDNA and established the location of the TSS of the human AβPP gene utilized cloned RdDp (reverse transcriptase), whereas Mita’s group employed viral preparation of RdDp. The former does not contain RNAse H activity, and the association with its template in a double-stranded structure prevented AβPP cDNA from folding into a self-priming conformation. In contrast, the latter contains RNAse H activity, which, upon completion of reverse transcription, removes the mRNA template and enables the formation of the self-priming structure and its subsequent expansion.

## 25. RNA-Based Therapeutic Approaches for Alzheimer’s Disease: Anti-Antisense Oligonucleotides (AASO) Targeting AβPP Antisense RNA Are Potential AD Drugs

As stated above, in the ACH2.0 paradigm, the ultimate goal of AD therapy, both in the prevention and treatment of the disease, is either to prevent activation of the AβPP-independent C99 production pathway or to disable it if/when it is already operational. The preceding sections defined two principal requirements for the occurrence of the RNA-dependent mRNA amplification process. One is the sustained ISR state, which enables the production of components that are essential for this process. Another is the eligibility of an mRNA species for such a process. The preceding sections also established that human AβPP mRNA is, apparently, eligible for RNA-dependent amplification. All therapeutic approaches for AD discussed in the preceding portion of the present perspective addressed only the first requirement and targeted, either directly or indirectly, the elicitation and/or maintenance of the neuronal ISR state. Therapeutic approaches described in the present and following sections address the second requirement and target the eligibility of human AβPP mRNA for the RNA-dependent amplification process.

One of the guiding principles in any therapeutic strategy for AD is that it should not interfere with the normal physiological production of AβPP and Aβ. Both AβPP and its derivative, Aβ, are physiologically beneficial [255,256,257]; interference, especially long-term interference, with the production of either would potentially be detrimental. This principle has been adhered to in the design of therapeutic strategies described above; therapeutic approaches targeting the eligibility of human AβPP mRNA and described below also abide by it.

The simplest way to interfere with the eligibility of human AβPP mRNA for the RNA-dependent amplification process without affecting the normal physiological production of AβPP and Aβ is to target not AβPP mRNA but its antisense counterpart. Indeed, AβPP mRNA is important in the RNA-dependent amplification process only insofar as it encodes and enables the production of the antisense RNA. It is the latter that actively and intimately enacts the amplification process. At this level, the interference with the amplification process translates into interference with the folding of the antisense RNA into a self-priming double-stranded structure. An effective way to accomplish such an interference is by employing anti-antisense oligonucleotides (AASO). Logically, the prime targets for such AASOs are the TCE or ICE elements; interference with either would prevent the TCE/ICE interaction by making one of them unavailable; this would preclude the formation of the self-priming structure. But the availability of the TCE and ICE is only one prerequisite for the formation of the self-priming structure. Another is their mutual accessibility within the folded antisense RNA molecule. Therefore, *any* AβPP AASO targeting *any* segment of the AβPP antisense RNA other than TCE and ICE could potentially alter the folding of the AβPP antisense RNA, render the TCE and ICE elements mutually inaccessible, and thus avert the formation of the self-priming double-stranded structure. The unpredictability of RNA folding makes it impossible to predict which AβPP AASO would interfere most effectively with the mutual accessibility of the TCE and ICE of the AβPP antisense RNA. However, different AβPP AASOs can be screened for this ability both in an in vitro assay and in cell-based AD models described below.

It should be noted that AASOs would be highly selective drugs for AD in that they would be effective only in the AD-affected neuronal cells, because only in these cells would antisense AβPP RNA occur. Moreover, in the AD-affected neurons, the oligonucleotide strategy would be effective only in the AASO, not ASO format. The reason for this is that, as described above, in neurons where the RNA-dependent AβPP mRNA amplification pathway is operational, both the progenitor (i.e., genome-transcribed) AβPP mRNA and the chimeric RNA end product are heavily modified and are, therefore, not amenable to stable interaction with complementary nucleic acid molecules. In contrast, antisense AβPP RNA produced in the chimeric amplification cycle remains unmodified and thus susceptible to the AASO interference. It follows that the ASOs for AβPP mRNA could interact with its target only in the neurons *not* affected by AD, because only in these cells would AβPP mRNA be unmodified. Therefore, the ASO strategy for AβPP mRNA has only preventive potential. By suppressing production of AβPP and, consequently, of Aβ, it would lower the rate of accumulation of iAβ and extend the time required for the T1 crossing. However, it would interfere with the normal physiological production of AβPP and Aβ, and because of the benefits conferred on neurons by both, such a strategy could actually be detrimental rather than therapeutic.

## 26. RNA-Based Therapeutic Approaches for Alzheimer’s Disease: Shift in the TSS of Human AβPP mRNA

**Figure 25:**
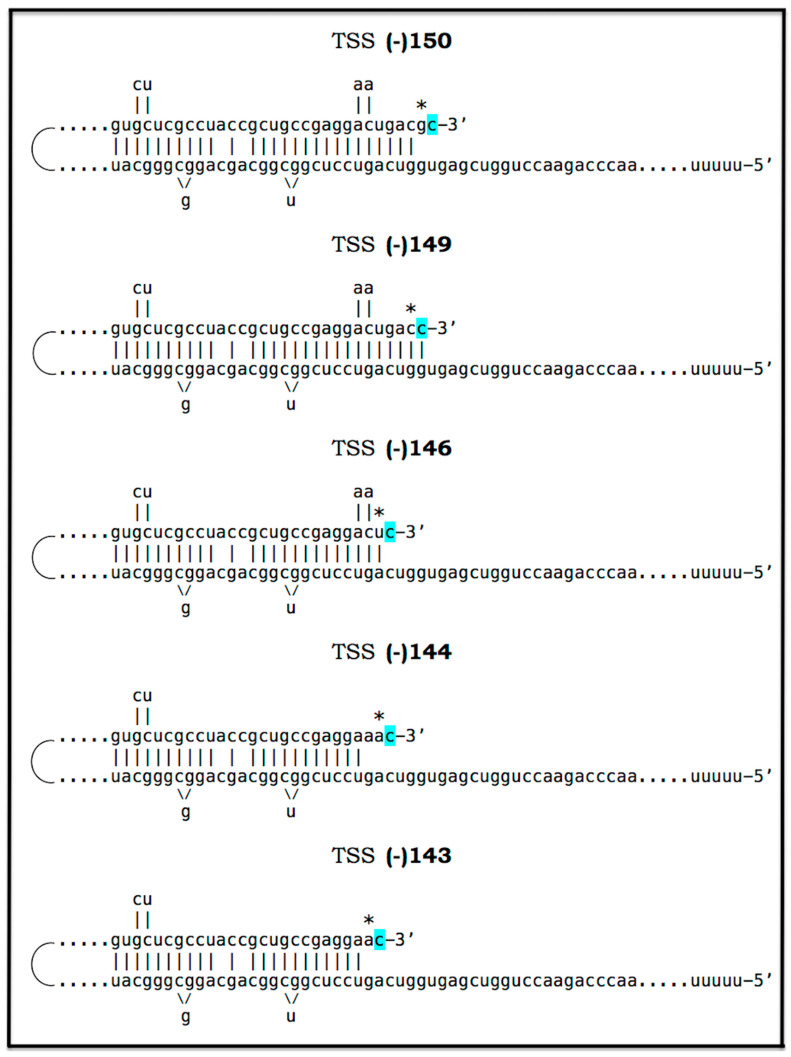
**Putative folding of antisense RNA molecules transcribed from human AβPP mRNA initiated at various TSSs**. *TSS*: Transcription start site. *(-)150, (-)149, (-)146, (-)144, and (-)143*: Identified TSSs of human AβPP mRNA. Numbers represent distances in number of nucleotides upstream from the “A” of the translation-initiating AUG codon encoding Met671 of AβPP. *Asterisk*: The nucleotide of the antisense RNA molecule transcribed from the TSS-forming nucleotide of human AβPP mRNA. *Highlighted in blue*: “C” transcribed from the 5′-terminal cap “G” of AβPP mRNA, which is not encoded in the genome. Only the antisense RNA strand originating from human AβPP mRNA transcribed from the TSS (-)149 is capable of forming a self-primed structure without the 3′-overhang.

An alternative approach to render human AβPP mRNA ineligible for the RNA-dependent amplification process is to interfere with its transcription. The ultimate goal of this approach is the same as formulated in the preceding section, namely, to prevent the AβPP antisense RNA from forming a self-priming double-stranded structure. This can be accomplished by shifting the position of the transcription start site (TSS) of the human AβPP gene. One possible strategy is the modulation of the usage of multiple TSSs of the human AβPP gene. The AβPP gene belongs to the category of TATA-less genes. “TATA” is a promoter element located typically thirty nucleotides upstream from the TSS of a gene. TATA-containing genes usually have a single TSS, and its position is rigidly defined by the TATA element. In contrast, TATA-less genes (the majority of housekeeping genes belong to this category) have multiple TSSs, as exemplified by the human AβPP gene. As shown in Figure 25, the human AβPP gene has five known TSS positions [251], defined as the number of nucleotides (and expressed as negative numbers) upstream from “A” of the ATG corresponding to the AUG translation initiation codon of AβPP mRNA. The TSS positions of the human AβPP gene are the following: (-150), (-149), (-146), (-144), and (-143). Interactions of 3′-terminal segments of the AβPP antisense RNA transcribed by RdRp from human AβPP mRNA initiated at various TSSs are illustrated in Figure 25. This Figure takes into account that RdRp transcribes the capG of mRNA, which is added post-transcriptionally and is not encoded in the genome.

Figure 25 makes it patently clear that only the human AβPP antisense RNA transcribed from mRNA initiated at the position (-149) possesses the authentic 3′TCE element. As a reminder, “T” in the TCE stands for “terminal”. The TCE is defined as strictly 3′-terminal. This is an essential requirement for the extension of the self-priming RNA structure. If this requirement is not met, as shown in Figure 25 for the folded AβPP antisense RNA molecules produced from mRNAs initiated at positions (-150), (-146), (-144), and (-143), the extension would either not occur or occur inefficiently. Moreover, it is not inconceivable that the occurrence of TSS at a position other than (-149) may interfere with the folding of the antisense RNA.

Thus, it appears that only human AβPP mRNA initiated at the position (-149) of the human AβPP gene produces the antisense RNA counterpart possessing the authentic 3′TCE element and, therefore, that only this human AβPP mRNA variant is eligible for the RNA-dependent amplification process. Therefore, small molecule-mediated modulation of the usage of TSSs of the human AβPP gene away from the position (-149) and toward other TSS positions, which would result in AβPP mRNA variants ineligible for or ineffective in the RNA-dependent amplification process, could be a highly effective therapeutic strategy for AD.

Small molecule-mediated modulation of the usage of TSSs of the human AβPP gene requires transcriptional shifts as small as a single nucleotide or, at most, a very few nucleotides, which are potentially easier to enact than shifts over longer distances. However, shifts in TSS of the human AβPP gene over longer distances, both upstream and downstream, could be equally, if not more, effective as therapeutic options for AD. Indeed, a significant shift upstream from the physiological range of TSSs of the human AβPP gene would result in a long 3′ overhang within the folded AβPP antisense RNA (provided that the ICE and its complement, which is no longer the TCE, are still mutually accessible).

Alternatively, TSS shift could drastically change the folding pattern of the AβPP antisense RNA and render the ICE and its complement mutually inaccessible, which would exclude the possibility of the 3’ extension. On the other hand, a significant downstream shift occurring within the segment of the human AβPP gene encoding the 5′UTR of AβPP mRNA would either shorten the TCE of the antisense AβPP RNA so that its association with the ICE would become unstable, or would eliminate the TCE element altogether. Both types of shifts would make the resulting AβPP mRNA ineligible for the RNA-dependent amplification process. Importantly, in all manipulations described in the present sub-section, the resulting AβPP mRNA would retain its coding region, and the conventional production of AβPP and, subsequently, of Aβ would be unaffected.

## 27. RNA-Based Therapeutic Approaches for Alzheimer’s Disease: Genomic Editing-Mediated Restructuring of the 5′ Terminus of AβPP mRNA

The RNA-based therapeutic approach discussed in the present section accomplishes the same goal as the therapeutic strategies described in the preceding sections: it renders human AβPP mRNA ineligible for the RNA-dependent amplification process. This strategy is capable of achieving everything described in the preceding sections, but with greater precision and control, and is principally different only in the manner of its implementation. The essence of this therapeutic approach is genomic editing of the human AβPP gene in its portion encoding the TCE element of the AβPP antisense RNA. Indeed, while the small molecule-mediated modulation of the TSS usage away from the TSS in the position (-149) of the human AβPP gene would only reduce the frequency of the utilization of this TSS and, consequently, the proportion of AβPP mRNA transcripts initiated at this position, the removal or replacement of possibly a single, and certainly no more than a few, nucleotides by genomic editing would ascertain that no AβPP mRNA transcripts are initiated at this position.

In another example, genomic editing tools can remove either a portion, or the entire segment of the human AβPP gene encoding the TCE element of the AβPP antisense RNA. The former would reduce the stability of the TCE/ICE interaction, whereas the latter would eliminate this interaction altogether. Moreover, using the same tools, it is possible to replace either a portion or the entire segment of the human AβPP gene encoding the TCE element of the AβPP antisense RNA with a “neutral” nucleotide sequence in such a way as not only to eliminate the TCE/ICE interaction but also to minimize the effect of editing on the rates of both transcription and translation of AβPP mRNA. Since all manipulations described in the present section would affect, in one way or another, only a minor fragment of the AβPP gene encoding the TCE of the antisense RNA, neither the coding region of AβPP mRNA nor the conventional production of AβPP and Aβ would be impacted. On the other hand, these manipulations would render AβPP mRNA ineligible for the RNA-dependent amplification process.

## 28. Current Transgenic “AD Models” Are Patently Inadequate: They Model Not AD but the Deleterious Effects of the Sustained Neuronal ISR

All therapeutic strategies described in the present perspective have to be tested in an appropriate AD model. However, all current transgenic animal models overexpressing Aβ and widely utilized as AD models are patently inadequate. Moreover, they are simply not AD models. They do achieve an increased deposition of extracellular Aβ, and they do exhibit a degree of neurodegeneration and limited cognitive impairment, but they do not present the major hallmark of the disease, namely, neurofibrillary tangles (NFT). As discussed above, the deposition of extracellular Aβ is not necessarily a feature of AD, and neurodegeneration and cognitive impairment, both not rising to the extent seen in the disease, can be safely attributed to the overexpression of Aβ leading to accelerated accumulation of iAβ above the T1 threshold and the resulting ISR state, but not to AD (further discussed in the present section below).

In the ACH2.0 paradigm, the reason why transgenic animal models overexpressing human AβPP (typically carrying an array of mutations either causing or associated with the disease in humans) are not AD models is that they are incapable of producing C99 in the AβPP-independent pathway. Within the framework of the ACH2.0, this incompetence can potentially be due to two causes. One is the lack of the operational RNA-dependent mRNA amplification mechanism in mice. This cause, however, can be ruled out. Indeed, robust RNA-dependent mRNA amplification was shown to occur in mice in processes such as the terminal differentiation [172,173] and deposition of extracellular matrix [174]. The second possible reason why mice cannot generate C99 via the asymmetric amplification of AβPP mRNA is that the latter is not eligible for the RNA-dependent amplification process. As discussed below, this is, apparently, indeed the case.

**Figure 26:**
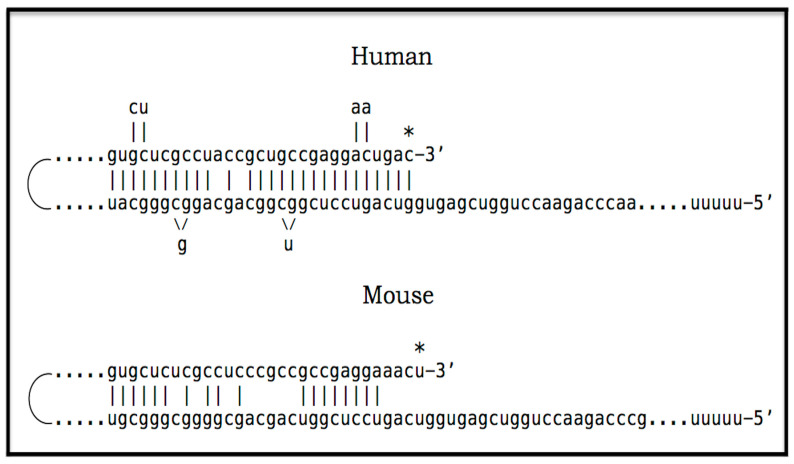
**Mouse AβPP mRNA is illegitimate for the RNA-dependent mRNA amplification process**. *“Human”*: Relevant portions of human antisense AβPP RNA shown in the self-primed conformation. *“Mouse”*: The relationship between portions of the mouse antisense AβPP RNA molecule analogous to the TCE and ICE elements of the human antisense AβPP RNA. *Asterisk:* The nucleotide of the AβPP antisense RNA molecule corresponding to the TSS-forming nucleotide of either human or mouse AβPP mRNA. The TSS (-149) was considered in both cases because in humans it yields amplification-eligible mRNA and in mice it serves as a major transcription initiation site. While substantial complementarity between the TCE and ICE of human antisense AβPP RNA is evident, there is no better than random complementarity between portions of mouse antisense AβPP RNA corresponding to the TCE and ICE elements of its human counterpart, and the 3′ overhang would effectively preclude the extension. The possibility that the 3′-terminal portion of the mouse AβPP antisense RNA constitutes a TCE, with the matching ICE situated somewhere else in the molecule, was excluded by the blast analysis of the 3′-terminal segment of the mouse antisense AβPP RNA with the rest of the molecule, which showed no significant complementarity anywhere. Mouse AβPP mRNA is, consequently, an illegitimate template for the RNA-dependent mRNA amplification process.

As stated above, the two requirements of the eligibility of an mRNA species for RNA-dependent amplification are the occurrence of the TCE and ICE elements within its antisense counterpart and, provided they do occur, mutual accessibility of these elements in the folded conformation of the antisense strand. However, as shown in Figure 26, even the initial requirement, that of the presence of the TCE and ICE, is not met in the mouse AβPP antisense RNA. Figure 26 compares the complementary relationship between the TCE and ICE elements of human AβPP antisense RNA with that of corresponding segments of mouse AβPP antisense RNA. While there is extensive complementarity and no 3′ overhang in the former, complementarity in the latter is no better than random. Even if these segments align in the folded configuration of the antisense strand, it is inconceivable that they will form a stable structure. Moreover, the presence of an extensive 3′ overhang would, in any case, prevent priming and extension. It could be argued that in mouse AβPP antisense RNA, its 3′-terminal segment has the matching ICE, but in a position not corresponding to the position of the human ICE. This, however, is not the case: The blast analysis showed that the 3′-terminal segment of the mouse AβPP antisense RNA has no significant complementarity with the rest of the molecule. It follows, therefore, that mouse AβPP mRNA is ineligible for the RNA-dependent amplification process.

It could be further argued that ineligibility of mouse AβPP mRNA for RNA-dependent amplification is irrelevant because AβPP mRNA molecules overexpressed in mouse models from multiple transgenes are of human origin and, therefore, should ostensibly be capable of functioning as amplification templates. Evidently, they cannot. The reason for this is that during the construction of transgene-containing vectors, the 5′-terminal segment of the human AβPP transgene, which encodes the TCE element of the AβPP antisense RNA, is heavily restructured. As a result, in the human AβPP antisense strands derived from such a transgene the TCE element is either rendered non-terminal or removed. Consequently, the AβPP antisense RNA originated from such a transgene cannot form a self-priming structure, and thus, transgenes-transcribed human AβPP mRNA is ineligible for the RNA-dependent amplification process (principally, for the same reason that mouse AβPP mRNA is).

**Figure 27:**
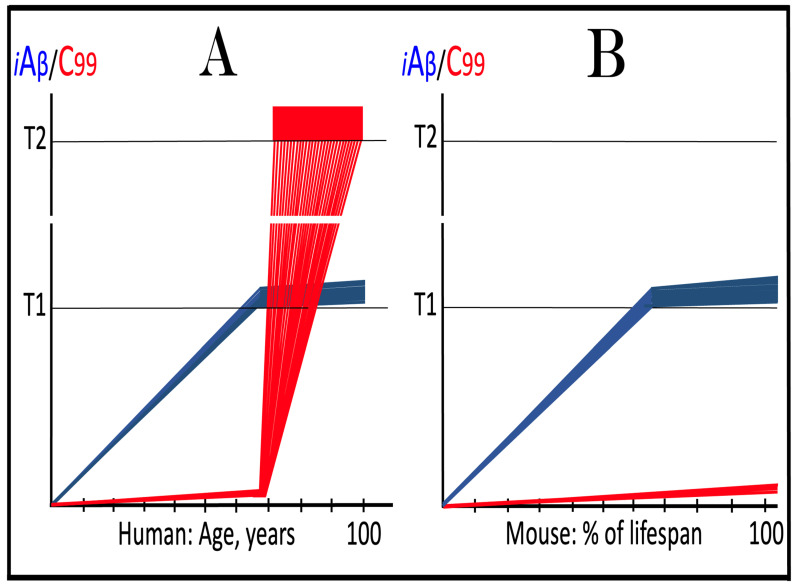
**Comparative dynamics of accumulation of iAβ and C99 in the conventional AD patient and in the mouse overexpressing AβPP from numerous human AβPP mRNA-derived transgenes**. *Vertical axis*: Relative levels of iAβ and C99. *Horizontal axis*: Age, in years (human) or percent of lifespan (mouse). *Blue lines*: Dynamics of iAβ. *Red lines*: Dynamics of C99. **T1**: Threshold of intracellular concentration of iAβ or C99 that mediates elicitation of the neuronal integrated stress response, which, in turn, enables operation of the AβPP-independent C99 generation pathway and thus triggers the disease. **T2**: Threshold of intracellular concentration of C99 that triggers neuronal death. *Red box*: Range of intracellular concentrations of C99, dubbed “Apoptotic Zone”, associated with apoptosis or necroptosis of neuronal cells. Panel (**A**): iAβ accumulates, via cellular uptake of secreted extracellular Aβ and through retention of iAβ resulting from the processing of a fraction of AβPP-derived C99 on intraneuronal membranes. When its levels cross the T1 threshold, the PKR and/or HRI kinases are activated; eIF2α is phosphorylated at its residue Ser51, and the neuronal ISR is elicited. Under ISR conditions, production of AβPP, BACE enzymes, and gamma-secretase is inhibited along with that of the bulk of cellular proteins. Consequently, the accumulation of iAβ is suppressed. Concurrently, under ISR conditions, components essential for the operation of the RNA-dependent mRNA amplification pathway are produced, and the pathway is activated. Human AβPP mRNA is eligible for this process. Its amplification is asymmetric and results in a massive production of the chimeric RNA end product encoding the C100 fragment of AβPP, which is subsequently processed into C99. RNA-dependent human AβPP mRNA amplification pathway is autonomous, with C99 propagating the neuronal ISR state and perpetuating its own production in an AβPP-independent manner. C99 rapidly accumulates and drives AD pathology; when its levels cross the T2 threshold, neuronal loss ensues. Panel (**B**): In mouse neurons, iAβ accumulates via the same two pathways, but, due to the two orders of magnitude greater scale of exogenous AβPP production, its accumulation is much more rapid than in the human neurons. When the T1 threshold is crossed, eIF2α kinases PKR and/or HRI are activated, and the neuronal ISR is elicited. Production of AβPP (both exogenous and endogenous) is inhibited, and accumulation of AβPP-derived iAβ and C99 is suppressed. Presumably, under neuronal ISR conditions, the essential components of the RNA-dependent mRNA amplification pathway are produced, and the pathway is rendered operational. However, neither mouse AβPP mRNA nor human transgene-transcribed AβPP mRNA are eligible for the RNA-dependent amplification process. Such models exhibit a degree of neurodegeneration and cognitive impairment, but both are consequences of the sustained neuronal ISR and are not symptoms of AD (see main text).

The comparative dynamics of accumulation of iAβ and C99 in the conventional AD patient and in the mouse overexpressing AβPP from numerous human AβPP mRNA-derived transgenes are illustrated in Figure 27. In humans (panel A of Figure 27), AβPP-derived iAβ accumulates via two pathways: cellular uptake of secreted extracellular Aβ and intracellular retention of Aβ resulting from gamma-cleavage of AβPP-derived C99 on intracellular membranes. When levels of iAβ reach and cross the T1 threshold, the PKR and/or HRI kinases are activated, eIF2α is phosphorylated at its Ser51 residue, and the neuronal ISR is elicited. Global cellular protein synthesis, including that of AβPP and, consequently, Aβ, is inhibited, and accumulation of iAβ is suppressed. On the other hand, under ISR conditions, components essential for the operation of the RNA-dependent mRNA amplification pathway are produced, and the pathway is activated. Human AβPP mRNA is eligible for this process. Its amplification is asymmetric and results in a massive production of the chimeric RNA end product encoding the C100 fragment of AβPP, which is subsequently processed into C99. RNA-dependent human AβPP mRNA amplification pathway is autonomous, with C99 propagating the neuronal ISR state (see Section 4) and perpetuating its own production in an AβPP-independent manner. C99 rapidly accumulates and drives AD pathology; when its levels cross the T2 threshold, neuronal apoptosis or necroptosis follows.

To a point, similar processes take place in mouse neurons overproducing human AβPP (panel B of Figure 27). iAβ accumulates via the same two pathways, but, due to the two orders of magnitude greater scale of exogenous AβPP production, iAβ accumulates much more rapidly than in human neurons. When the T1 threshold is crossed, eIF2α kinases PKR and/or HRI are activated, and the neuronal ISR is elicited. Production of AβPP (both exogenous and endogenous) is inhibited, and accumulation of AβPP-derived iAβ and C99 is suppressed. Presumably, under neuronal ISR conditions, the essential components of the RNA-dependent mRNA amplification pathway are produced, and the pathway is rendered operational. However, neither mouse AβPP mRNA nor human transgene-transcribed AβPP mRNA are eligible for the RNA-dependent amplification process.

Thus, neither RNA-dependent amplification of AβPP mRNA nor AβPP-independent production of C99 takes place in current transgenic animal “AD models”. Following elicitation of the neuronal ISR, accumulation of AβPP-derived iAβ is suppressed, but presumably it remains at over-T1 levels. It reaches neither the T2 threshold nor the AD pathology-driving range of cellular concentrations, and no AD develops. On the other hand, it does sustain the neuronal ISR state, and it is this state that causes effects, namely, limited neurodegeneration and cognitive impairment that were previously erroneously attributed to AD. The ISR in general and neuronal ISR in particular are manifested as global suppression of cellular protein synthesis. When the ISR state is sustained, protein deficiencies result in cellular degeneration, a phenomenon observed in multiple cell types. The same phenomenon occurs in the neurons under the sustained neuronal ISR and is manifested as neurodegeneration [189,190,191,192,193,194,195,196,197,198,199,200,201,202,203,204]. As for the limited cognitive impairments observed in the current transgenic “models” of AD, they comprise deficiencies in learning, in neuronal plasticity, and in memory formation. All these processes were shown to be dependent on de novo neuronal protein synthesis. Under the sustained neuronal ISR-caused suppression of cellular protein synthesis, both neurodegeneration and cognitive impairment are inevitable.

The bottom line is that both neurodegeneration and limited cognitive impairments are caused by the sustained neuronal ISR and are not symptoms of AD. The cause and effect relationship between the neuronal ISR and cognitive impairments in the current transgenic “models” of AD are strongly indicated by the observation that prevention of the ISR, including the neuronal ISR, by its systemic inhibition in the mouse models precludes cognitive impairments, and that the reversal of the ISR state and restoration of neuronal protein synthesis also abrogates cognitive impairments [205,206,207,208,209,210,211,212,213]. The conclusion, therefore, is that transgenic animals overexpressing human AβPP and that are considered “AD models” model, in fact, the effects of the sustained neuronal ISR but not Alzheimer’s disease.

## 29. Human Neuronal Cells-Based Models for Conventional and Unconventional AD

While the availability of animal models of AD is crucial for the advancement of the field, many fundamental aspects of the disease can be addressed and various therapeutic strategies tested in human neuronal cell-based models of AD. The two principal advantages of such models are that (1) that they originate from the species affected by AD and therefore would present the “authentic” progression of AD pathology, and (2) that, by definition, all molecular mechanisms involved in AD pathology, including, presumably, the RNA-dependent AβPP amplification mechanism, are operational in properly stimulated human neuronal cells. As discussed in the preceding sections of the present perspective, in the ACH2.0 paradigm, the disease is triggered by sustained elicitation of the neuronal integrated stress response. It follows that if the sustained neuronal ISR were elicited by any means in human neuronal cells, AD pathology would ensue. If the elicitation of the neuronal ISR were triggered by the accumulation of iAβ over the T1 threshold, the resulting model would represent conventional AD. On the other hand, if the elicitation of the neuronal ISR occurs at the low, sub-T1 levels of iAβ and is mediated by unconventional stressors, i.e., stressors other than iAβ, the human neuronal cell-based model would represent the unconventional form of the disease. The best possible proof that such models faithfully represent the progression of cellular pathology in AD would be the occurrence of the major marker of the disease, namely, NFTs. The proof of plausibility of such human neuronal cells-based models of AD has been provided, albeit inadvertently, by the results of Choi and co-workers [258].

The aim of the study by Choi and co-workers was to maximize the production, secretion, and deposition of extracellular Aβ by cultured human neuronal cells. The study was designed within the framework of the ACH theory of AD, and the reasoning behind it was that, in the ACH paradigm, increased (in comparison with other models) levels of extracellular Aβ would trigger and stimulate AD pathology, and would drive it to stages unachievable in other experimental models. The major strategic innovation to maximize deposition of extracellular Aβ in close proximity to neurons was to culture human neuronal cells in semi-solid medium, matrigel, in order to minimize diffusion of secreted Aβ. This was accompanied by an experimental approach designed to maximize the exogenous production of AβPP and to augment its AD pathology-causing potential. For these purposes, Choi and co-workers used a multicistronic lentiviral vector to express AβPP, as well as PSEN1, both carrying FAD-causing mutations (two in AβPP and one in PSEN1). The result was nothing short of a triumph. The experimental approaches employed achieved an outcome never seen before in an experimental model, namely the formation of NFTs [258].

Choi and co-workers presented their results [258] as a confirmation of the principal tenets of the ACH theory of AD. In their interpretation, elevated levels of extracellular Aβ somehow drove AD pathology to extents never achieved before in experimental systems. However, the very same results can also be explained, distinctly differently and with a great degree of validity, in the ACH2.0 paradigm. All efforts of the investigators assured rapid accumulation of iAβ. Indeed, expression of exogenous AβPP from lentiviral vectors resulted in greatly increased secretion of Aβ and equally increased retention of iAβ generated from a fraction of C99 processed on intraneuronal membranes. Since cells were cultured in semi-solid medium, the secreted Aβ did not diffuse. Due to its greatly increased extracellular concentration, its cellular uptake increased accordingly, further accelerating the accumulation of iAβ. Moreover, all three FAD mutations employed in this study significantly augmented the accumulation of iAβ. Thus, the two mutations employed, the London mutation of AβPP (V717I) and the ΔE9 mutation of PSEN1, greatly increased production of the Aβ42 variant. As discussed above, this variant is imported by neurons from the extracellular space twice as effectively as other Aβ variants. Moreover, due to its increased cellular toxicity (relative to other iAβ variants), iAβ42 lowered the extent of the T1 threshold [11]. The third mutation employed, the Swedish mutation of AβPP (K670N/M671L), strongly augmented the proportion of C99 undergoing gamma-cleavage on intraneuronal membranes, and thus yielded iAβ. As a result, iAβ, mostly of exogenous origin, rapidly accumulated and crossed the T1 threshold. The neuronal ISR was elicited, and the RNA-dependent mRNA amplification pathway was activated. For reasons explained in the preceding section, exogenous vector-transcribed AβPP mRNA is ineligible for RNA-dependent amplification. However, endogenously produced human AβPP mRNA is eligible for asymmetric RNA-dependent amplification. The resulting C99 fragment of AβPP rapidly accumulated and drove AD pathology, culminating in the formation of NFTs and, eventually, neuronal death. These results, therefore, constitute the proof of plausibility of adequate AD models based on human neuronal cells.

In the ACH2.0 paradigm, the convoluted arrangements employed in [258], including the requirement to culture cells in semi-solid medium, are completely unnecessary in order to achieve a sufficient accumulation of iAβ, eventually leading to the formation of NFTs. Indeed, in terms of the ACH2.0, extracellular Aβ is irrelevant for AD, except as a source of iAβ. To assure rapid and highly efficient accumulation of iAβ in cultured human neuronal cells, Aβ, more specifically Aβ42, alone should be produced exogenously from expression vectors or in an especially constructed stable cell line. To emphasize, Aβ and not AβPP or C99 should be expressed. This is because if AβPP or C99 were expressed, the bulk of Aβ resulting from their processing would be secreted, whereas the entire yield of Aβ42 produced from the expression vector encoding only this peptide would be retained intraneuronally as iAβ. The expression vector can be based on a fragment of AβPP DNA. The ATG encoding Met671 of AβPP, situated in the optimal translation initiation nucleotide context, can be used as the first codon of the coding region of the expression vector and the codon encoding amino acid residue 713 of AβPP as its last. The advantage of expressing the Aβ42 variant in this context is that, as discussed above, due to its toxicity, it lowers the extent of the T1 threshold: less Aβ42 (in comparison with other iAβ variants) is needed to trigger the elicitation of the ISR. This, in turn, enables activation of an autonomous, self-sustaining endogenous RNA-dependent AβPP mRNA amplification pathway. At this point, exogenous expression of Aβ42 becomes redundant (C99 produced from asymmetrically amplified endogenous AβPP mRNA maintains the ISR state and thus perpetuates its own production). In such a model system for conventional AD, C99 would rapidly accumulate and drive AD pathology, and NFTs would ensue. Thus, NFTs constitute the major attribute of this model system; it reports on the occurrence of AD pathology. Additional principal reporters in this system are C100 (N-terminal Met-C99), which denotes initiation of translation from the AUG encoding Met671 of AβPP, and defined sense/antisense RNA junction sequences [11], which report on the operational RNA-dependent AβPP mRNA amplification pathway.

In the ACH2.0 paradigm, the sustained elicitation of the neuronal integrated stress response is all it takes to activate the RNA-dependent asymmetric amplification of the eligible endogenous human AβPP mRNA and thus to ignite AD pathology. This notion defines the way to generate a human neuronal cell-based model of unconventional AD. As a reminder, unconventional AD arises when the ISR is sustainably elicited in the human neurons by stressors other than iAβ (i.e., unconventional stressors). In terms of the ACH2.0, the sustained elicitation of the integrated stress response in cultured human neuronal cells via application of unconventional stressors, such as, for example, stressors triggering mitochondrial distress and activating the OMA1 to DELE1 to HRI signaling pathway, would produce a model of unconventional AD. In such a model, essential components produced under ISR conditions would enable operation of the RNA-dependent mRNA amplification pathway. AβPP mRNA endogenously produced in human neuronal cells is an eligible template for this pathway. Its asymmetric amplification yields the chimeric RNA end product encoding C100 that is subsequently processed into C99. Thus generated C99 rapidly accumulates and crosses the T1 threshold. At this point, unconventional stressors become redundant, and the RNA-dependent AβPP mRNA amplification pathway is rendered self-sustaining. Continuously accumulating C99 drives AD pathology, which culminates in the formation of NFTs and, eventually, neuronal death. As in the conventional AD model described in the preceding paragraph, the principal reporters of the human neuronal cell-based model of unconventional AD are NFTs, C100, and defined sense/antisense RNA junction sequences reporting on the progression of AD pathology, initiation of translation from the AUG encoding Met671 of AβPP, and operation of the asymmetric RNA-dependent AβPP mRNA amplification pathway, respectively.

## 30. Adequate Transgenic Animal Models for Conventional and Unconventional AD

As pointed out above, although human neuronal cell-based systems are sufficient to address many fundamental aspects of, and to evaluate therapeutic strategies for, AD, animal models of Alzheimer’s, in both its conventional and unconventional forms, are essential for the progress of the field. To construct an adequate transgenic animal model for conventional AD, two requirements should be met. One is the expression by neuronal cells of human AβPP mRNA that is unadulterated in any of its portions, including 5′UTR. This mRNA would be eligible for the asymmetric RNA-dependent amplification process. The resulting chimeric RNA end product would encode C99, which would rapidly accumulate and drive AD pathology. Results obtained with human neuronal cells [258] suggest that even one copy of the gene encoding amplification-eligible human AβPP mRNA could be sufficient to drive, via the amplification of its mRNA transcripts, AD pathology. Multiple copies would significantly accelerate the development of the disease.

Another requirement for the generation of an animal model for conventional AD is the accumulation of iAβ in neuronal cells to levels (above the T1 threshold) that trigger elicitation of the neuronal integrated stress response. This, in fact, had already been achieved in numerous current animal models overexpressing human Aβ derived from AβPP translated from human AβPP mRNA. This mRNA is transcribed from multiple transgenes and is, as discussed above, ineligible for RNA-dependent amplification. Transgene-originated iAβ accumulates in the neurons via the importation of secreted extracellular Aβ and through the retention of Aβ resulting from the processing of a fraction of AβPP-derived C99 on intraneuronal membranes. When it crosses the T1 threshold, the neuronal ISR is elicited, which, in turn, activates the RNA-dependent mRNA amplification pathway. Overexpression of Aβ from multiple wild-type human transgenes was shown to be sufficient to elicit the neuronal ISR. Utilization of human AβPP transgenes carrying FAD-associated mutation, as well as concurrent employment of transgenes expressing FAD mutation-carrying PSENs and, finally, ApoE4, all of which accelerate the rate of iAβ accumulation, was shown to elicit the neuronal ISR much sooner. Therefore, one way to generate a transgenic animal model of conventional AD is to use current Aβ-overexpressing animal transgenic models and to add a single or multiple transgenes expressing human AβPP mRNA eligible for the RNA-dependent amplification process.

As discussed above, accumulation of iAβ to the neuronal ISR-eliciting levels can be accomplished much more efficiently by the exogenous overexpression of only Aβ (rather than its production by proteolysis of AβPP or C99), which would not be secreted but retained intraneuronally in its entirety as iAβ. Utilization of Aβ42 would maximize the efficiency of this approach because, due to its cellular toxicity, this Aβ variant reduces the extent of the T1 threshold (see above). Exogenous overexpression of Aβ42 in combination with the introduction of a single or multiple transgenes expressing human AβPP mRNA eligible for the RNA-dependent amplification process would satisfy both requirements stipulated above. Aβ42 would efficiently accumulate to over-T1 levels and trigger elicitation of the neuronal ISR. Under ISR conditions, essential components of the RNA-dependent mRNA amplification pathway would be produced, and the pathway would be activated. Appropriately designed human AβPP transgene(s) would supply amplification-eligible AβPP mRNA, the product of its asymmetric amplification would drive AD pathology, and the disease would ensue.

In practical terms, the fastest way to generate an appropriate transgenic animal model of conventional AD would be to utilize one of the currently available transgenic models overexpressing human AβPP. In these models, the neuronal ISR is sustainably elicited by iAβ accumulated over the T1 threshold, and the RNA-dependent mRNA amplification mechanism is presumably activated, but AD is not developed because transgene-transcribed human AβPP mRNA is adulterated at its 5′ terminus and is, therefore, ineligible for the RNA-dependent amplification process. Thus, the first requirement for an adequate AD model (i.e., sustained elicitation of the neuronal ISR) is satisfied in such a system, but the second requirement (i.e., the eligibility of AβPP mRNA for the RNA-dependent amplification process) is not. If, however, we employ genome-editing tools and modify human transgenes (a portion of them or even a single one) in such a way as to restore the 5′ terminus of human AβPP mRNA, this would restore its eligibility for the RNA-dependent amplification process and satisfy the second requirement. In such a model, exogenously produced AβPP-derived iAβ would trigger elicitation of the neuronal ISR, which, in turn, would activate the RNA-dependent mRNA amplification mechanism. The appropriately edited human AβPP transgenes would supply amplification-eligible AβPP mRNA, and the end product of its amplification would encode C100. C99 derived from C100 would rapidly accumulate, drive AD pathology, and the disease would ensue.

Design of transgenic animal models of unconventional AD presents a conundrum. On one hand, we have to introduce into a model human AβPP transgenes capable of expressing intact, RNA-dependent amplification-eligible AβPP mRNA. In an unconventional AD model, its amplification and the disease would be triggered by the neuronal integrated stress response elicited by unconventional, i.e., not iAβ, stressors. However, if AβPP were expressed constitutively, iAβ would accumulate via both the neuronal uptake of secreted extracellular Aβ and through the retention of Aβ resulting from gamma-cleavages of a fraction of C99 on intraneuronal membranes. Its levels could reach the T1 threshold, and in such a case, the neuronal ISR would be elicited and, consequently, the RNA-dependent AβPP mRNA amplification would be activated, and the disease would occur conventionally. To avoid this, the production of AβPP-derived Aβ and, consequently, the accumulation of iAβ should be prevented, at least prior to the unconventional elicitation of the neuronal ISR and commencement of unconventional AD.

The solution for the above conundrum is obvious: Express human AβPP transgenes capable of generating RNA-dependent amplification-eligible AβPP mRNA under the control of an inducible promoter and activate the promoter concurrently with or following the sustained elicitation of the neuronal integrated stress response by unconventional stressors. In the absence of exogenous iAβ, there would be no doubt regarding the origin of the neuronal ISR; it could be only unconventional. The selection of unconventional stressors would depend on the interest in a specific type of unconventional AD. Indeed, elicitation of the neuronal ISR can be triggered by a variety of stressors, including traumatic brain injury (TBI) and chronic traumatic encephalopathy (CTE). Both TBI and CTE act by lowering cerebral blood flow (CBF). CBF, in turn, was shown to trigger mitochondrial dysfunction in the neurons. This activates the OMA1 to DELE1 to HRI signaling pathway and results in the phosphorylation of eIF2α at its Ser51 residue and the elicitation of the neuronal ISR. The neuronal ISR can also be elicited by a variety of other unconventional stressors, such as direct activators of mitochondrial distress, direct activators of any of the eIF2α kinases, viral and bacterial infection, and different types of inflammation.

Following the unconventional elicitation of the neuronal ISR, the RNA-dependent mRNA amplification pathway would be activated. Simultaneous activation of the inducible promoter would initiate transcription of human AβPP transgenes and provide amplification-eligible AβPP mRNA. The chimeric end product of AβPP mRNA amplification encodes C99, which would rapidly accumulate and drive AD pathology. C99 would also propagate the neuronal ISR state and thus would perpetuate its own production via the AβPP mRNA amplification process. Due to the autonomous nature of the operational RNA-dependent AβPP mRNA amplification pathway, unconventional stressors would become redundant and could be withdrawn at this point. The neuronal ISR state would persist, and conventional translation of AβPP mRNA and subsequent production of AβPP-derived Aβ would be suppressed under ISR conditions. In such a model, therefore, there would be no questions regarding the unconventionality of the neuronal ISR and, consequently, of the disease.

## 31. Conclusions

### 31.1. Summary

The present perspective describes and analyzes therapeutic strategies for Alzheimer’s disease that collectively constitute the current state of the art in the ACH2.0 paradigm. All these strategies are based on our current understanding of AD, which can be briefly summarized as follows. Alzheimer’s disease comprises two categories: conventional and unconventional. In both categories, the event that actually instigates the disease is the sustained elicitation of the neuronal integrated stress response. In conventional AD, this event is triggered by AβPP-derived iAβ (referred to within the framework of the ACH2.0 as “conventional stressor”) accumulated over the critical T1 threshold. At these levels, iAβ mediates activation of the PKR and/or HRI kinases, which phosphorylate eIF2α at its Ser51 residue and thus elicit the ISR state. In unconventional AD, any stressor other than iAβ, and capable of activating one or more of the eIF2α kinases (in terms of the ACH2.0, these stressors are designated “unconventional stressors”), mediates elicitation of the neuronal ISR. Unconventional stressors are provided by multiple events and processes, such as TBI, CTE, bacterial and viral infections, and various types of inflammation (with some, like osteoarthritis, far distant from the brain). One of the apparently common denominators for the unconventional stressor-producing events is reduced CBF, which causes mitochondrial dysfunction resulting in the activation of the HRI kinase and subsequent sustained elicitation of the neuronal ISR.

Thus, the principal difference between the conventional and unconventional forms of AD is the manner of elicitation of the neuronal ISR. The major distinction between the two is that conventional sporadic AD elicitation of the neuronal ISR happens late in life because it takes decades for AβPP-derived iAβ to reach the ISR-eliciting T1 threshold, whereas in unconventional AD, unconventional stressors could be produced and the neuronal ISR elicited at any age. Once the neuronal ISR is sustainably elicited, the two forms of the disease largely merge. Under the neuronal ISR conditions, production of the bulk of cellular proteins (including AβPP and, consequently, AβPP-derived C99 and Aβ, as well as BACE enzymes and gamma-secretase) is suppressed. On the other hand, the production of some cellular proteins that are not normally expressed is enabled under the neuronal ISR. Among these are the components essential for operation of the powerful RNA-dependent mRNA amplification pathway, which is capable of amplifying eligible mRNA species at rates orders of magnitude greater than rates of their conventional gene-originated transcription; as a result, this pathway is activated.

Human AβPP mRNA is eligible for the RNA-dependent amplification process. Its amplification occurs asymmetrically and results in AβPP mRNA, which is severely 5′-truncated within its coding region. This RNA is heavily modified and is freely translatable under ISR conditions. Its first translation initiation codon is the AUG encoding Met671 of AβPP. Translation initiated from this codon yields the C100 (N-terminal Met-C99) fragment of AβPP, which is subsequently processed into C99. Due to the massive influx of C100-encoding mRNA, AβPP-independent production of C99 is highly effective. C99 is the driver of AD (in both its conventional and unconventional forms), and its rapid accumulation propagates progression of the disease, culminating in the formation of NFTs and, eventually, in neuronal loss. Interestingly and fittingly, production of NFT-comprising tau protein is also unaffected by ISR conditions: its translation is regulated by an IRES element situated within the 5′ UTR of its mRNA [153].

Importantly, the AβPP mRNA amplification-based C99 production pathway is autonomous and self-sustainable. This is because C99 generated in this pathway propagates and sustains the neuronal ISR state and thus perpetuates its own production. This implies that when the AβPP mRNA amplification-based C99 production pathway is rendered operational, the removal of the initial neuronal ISR-eliciting stressors, be it Aβ and, consequently, iAβ or unconventional stressors, would not impact in any way its activity and progression of the disease. This is why it is futile to treat conventional AD by removing extracellular Aβ (and thus suppressing its uptake as iAβ) or inhibiting BACE1 (and thus suppressing both the neuronal uptake of Aβ and retention of iAβ resulting from gamma-cleavages of C99 on intraneuronal membranes). This futility was convincingly demonstrated in numerous human clinical trials. As a general principle, the removal of neuronal ICR-eliciting stressors, including iAβ, is a valid therapeutic approach, but it would be effective only preventively, only when the neuronal ISR has not yet been elicited, and the AβPP mRNA amplification-based C99 production pathway is still inoperative. As an example, when lecanemab and donanemab were tested in very early AD [214,215,216,217,218], their therapeutic benefits were only marginal because the drugs only preventively affected the neurons where iAβ had not yet crossed the T1 threshold, but had no effect whatsoever on the neurons where the neuronal ISR had already been elicited and the AβPP mRNA amplification-based C99 production pathway activated.

It follows that the principal target in both the prevention and treatment of AD, in both its conventional and unconventional forms, is the activity of the AβPP mRNA amplification-based C99 production pathway. This target can be approached directly, via interference with the mechanism of amplification of AβPP mRNA, or indirectly, through suppression of the neuronal integrated stress response. Let us first consider the latter. The neuronal ISR is, indeed, a very attractive therapeutic target. Preclude its elicitation, and you will prevent activation of the AβPP mRNA amplification-based C99 production pathway and the disease. Reverse the neuronal ISR state when it is already established, and you will disable the AβPP mRNA amplification-based C99 production pathway and stop progression of the disease. Effective ISR inhibitors are currently available. They are, however, systemic and would be highly detrimental if administered long-term. Conceivably, ISR inhibitors could be developed that are neuron-specific or, even better, specific only for AD-affected neurons, or delivery systems for currently available ISR inhibitors could be constructed that are capable of delivering the drug specifically to the neurons or even only to the AD-affected neurons. In such a case, inhibition of the neuronal ISR state would become a viable therapeutic option for the prevention and treatment of AD.

Thus, currently, direct inhibition of the ISR is not feasible as a therapeutic option for AD. An alternative strategy to prevent or reverse the neuronal ISR state is to deplete stressors that elicit and/or sustain it. As discussed in the present perspective, this strategy is viable, and its application can potentially achieve spectacular outcomes. Pivotal to this approach is the ability to specifically degrade iAβ. Agents capable of degrading iAβ would also be effective in degrading C99, of which iAβ is the integral part; degradation of the Aβ segment of C99 would detoxify the latter and abolish its potential to sustain the neuronal ISR state. Agents capable of targeted degradation of iAβ include proteolysis-targeting chimeras (PROTACs) and molecular-glue degraders (MGDs). Among iAβ/C99 degrading agents are also physiological intra-iAβ cleaving activities of BACE1 and BACE2. Enhancers of these activities would be capable of the rapid depletion of iAβ and/or C99 and would, potentially, constitute powerful AD drugs. As an example, a single transient treatment by BACE1 and/or BACE2 activators or any other iAβ-degrading agents at midlife prior to the T1 crossing would deplete iAβ. Following the treatment, its de novo accumulation would resume from a low baseline. Its levels would not reach the T^0^ and T1 thresholds, and neither AACD nor conventional AD would occur within the remaining lifetime of the treated individual. A single transient application of the iAβ-degrading treatment in AACD patients (where the T^0^ threshold has been crossed but the T1 threshold has not yet been reached) would stop progression of the condition and prevent its recurrence for life.

In AD patients, with the AβPP mRNA amplification-based C99 production pathway operational, the influx of C99 is such that iAβ/C99 degradation alone is insufficient to deplete it. This, however, can be accomplished by a transient composite ISR inhibition and iAβ/C99 degradation treatment. In such a composite treatment, transient inhibition of the neuronal ISR disables the AβPP mRNA amplification-based C99 production pathway and abrogates the influx of C99, and concurrently administered iAβ/C99 degradation agents efficiently deplete both iAβ and C99. As a result, the neuronal ISR state is reversed, the operation of the AβPP mRNA amplification-based C99 production pathway ceases, and progression of the disease is arrested. De novo accumulation of iAβ resumes from a low baseline; its levels would not reach the T1 threshold, and conventional AD would not recur within the remaining lifetime of the treated individual. As described above, application of the composite ISR inhibition and concurrent iAβ/C99 degradation treatment would also be highly effective in the unconventional form of AD.

It should be noted that, for purposes of simplified discussion, the scenario considered in the present study is the one where production of AβPP and BACE enzymes is completely or nearly completely suppressed under neuronal ISR conditions. In such a scenario, when the neuronal ISR has been elicited, either conventionally or unconventionally, the production of AβPP and its processing into C99 and of C99 into iAβ are fully suppressed, and BACE activators can be employed only concurrently with ISR inhibitors. In reality, however, the residual production of AβPP and BACE enzymes probably continues under ISR conditions, and its extent remains to be ascertained. This implies that under ISR conditions, iAβ would continue to be produced not only by AβPP proteolysis but also by gamma-cleavages of a fraction of C99 generated independently of AβPP. This also implies that BACE activators would have some effect on the targeted degradation of iAβ/C99, even under ISR conditions. These considerations are fully consistent with the logic of the therapeutic approaches described above, and the usefulness of the administration of BACE activators under ISR conditions could be assessed when more empirical data are available. It should also be mentioned that one research group reported that AβPP mRNA appears to contain an IRES element within its 5′UTR [259]. This finding was not substantiated by other groups. However, even if it were correct, this would change neither the logic of the present study nor the principal outcomes of therapeutic strategies described above.

As for the direct interference with the RNA-dependent mRNA amplification mechanism in general, it would be unfeasible for the same reasons as the systemic inhibition of the ISR is. RNA-dependent mRNA amplification is one of the major physiological processes. It operates in terminal differentiation, in the deposition of extracellular matrix proteins, and, probably, in many other physiological settings. Therefore, its systemic suppression, especially long-term, would be highly detrimental. Nevertheless, this process can be interfered with, but in highly specific and carefully targeted ways. For RNA-dependent mRNA amplification to occur, two principal requirements must be met. One is that the components essential for the operation of this pathway, which are missing under regular conditions, must be produced. This is accomplished under ISR conditions. Another principal requirement is that, to be amplified, an mRNA species must be eligible for the amplification process. Human AβPP mRNA is, and its amplification eligibility aspect can be specifically, productively, and fruitfully interfered with. Thus, RNA-based therapeutic strategies for AD are simple: deprive human AβPP mRNA of its eligibility for RNA-dependent amplification, and you will either prevent the disease in both its conventional and unconventional forms or successfully treat it.

The eligibility of human AβPP mRNA for RNA-dependent amplification is based on the ability of its antisense RNA counterpart to form a stable self-priming structure. This ability is due to the presence within the antisense AβPP RNA of two complementary elements, the TCE (3′-terminal complementary element) and ICE (internal complementary element), which are mutually accessible in the folded configuration of the RNA molecule. Therefore, depriving human AβPP mRNA of its eligibility for the RNA-dependent amplification process equates to preventing stable TCE/ICE interaction. One way to accomplish this is by employing oligonucleotides complementary to the antisense AβPP RNA, in fact, anti-antisense oligonucleotides (AASO, in contrast to ASO, which are typically utilized for therapeutic purposes). The prime targets for the AASO interference would be the TCE or ICE elements of the antisense AβPP RNA. Interference with either of these elements would block their potential interaction and thus prevent the formation of a self-priming structure. The same result can also be potentially achieved with AASOs targeting any segment of the antisense AβPP RNA if it changes the folding of the RNA molecule in such a way that the TCE and ICE elements become mutually inaccessible. Effective application of the AASO strategy would render human AβPP mRNA ineligible for RNA-dependent amplification and would abrogate production of C99 in this pathway. Among the advantages of this strategy is that AASO-based drugs would be highly specific. Indeed, if properly designed, they would affect only the AD-affected neurons because only in these cells would antisense AβPP RNA occur. Another advantage of AASO-based drugs is that they would not interfere with the physiological production of Aβ in the AβPP proteolytic pathway.

There are two more therapeutically promising approaches to interfere with the eligibility of human AβPP mRNA for the RNA-dependent amplification process. One is to shift the position of the initiation of transcription of AβPP mRNA (i.e., of its transcription start site, TSS). The AβPP gene lacks the TATA promoter element and, consequently, has multiple TSSs. However, human AβPP mRNA initiated from only one of the five known TSSs can be efficiently amplified in the RNA-dependent amplification process. This is because only in its antisense RNA counterpart is the TCE element strictly 3′-terminal. In the antisense RNA counterparts of AβPP mRNA initiated from the other four TSSs, the TCE element is not 3′-terminal, and even if the TCE/ICE interaction occurs, its complex would have a 3′ overhang, which would interfere with self-priming of the folded antisense RNA. Therefore, small molecules capable of modulating the TSS usage of human AβPP mRNA away from the position generating the RNA molecule eligible for RNA-dependent amplification and toward other TSS positions would constitute potential AD drugs. Modulation of the TSS usage would require shifts over short distances, just a single or a few nucleotides. Shifts in TSS of the human AβPP gene over longer distances, both upstream and downstream, could be equally, if not more, effective as therapeutic options for AD. Thus, a significant shift upstream from TSSs of the human AβPP gene would result in a long 3′ overhang within the folded AβPP antisense RNA or would drastically change its folding, with both outcomes excluding the possibility of its self-primed extension. On the other hand, a significant downstream shift occurring within the segment of the human AβPP gene encoding the 5′UTR of AβPP mRNA would either shorten the TCE of the antisense AβPP RNA so that it association with the ICE would become unstable, or would eliminate the TCE element altogether. Both types of shift would make the resulting AβPP mRNA ineligible for the RNA-dependent amplification process. Importantly, such TSS shift strategies would not interfere with the coding region of the AβPP gene.

Another therapeutically promising approach to interfere with the eligibility of human AβPP mRNA for the RNA-dependent amplification process is to employ genomic editing tools with the goal of removing either a portion or the entire segment of the human AβPP gene encoding the TCE element of the AβPP antisense RNA. In either case, the antisense RNA counterpart of altered human AβPP mRNA would be unable to form a self-priming structure because the remaining portion of the TCE element is too short to interact stably with the ICE or the TCE is absent altogether. Moreover, a portion or the entire segment of the human AβPP gene encoding the TCE element of the AβPP antisense RNA can not simply be removed but replaced with a “neutral” nucleotide sequence in such a way as to minimize the effect of editing on the rates of both transcription and translation of AβPP mRNA. As a result, AβPP mRNA would retain its protein-coding potential but would be rendered ineligible for the RNA-dependent amplification process.

It should also be noted that therapeutic approaches analyzed in the present perspective are limited to those pertaining to the generation, degradation, and dynamics of accumulation of iAβ and C99. Therefore, due to the limitation of its scope, this narrative leaves out the potential role of cellular chaperones in AD pathology. Indeed, a large number of studies suggest that dysfunction of cellular chaperones and co-chaperones may contribute significantly to the pathogenesis of AD. Accordingly, cellular chaperones and co-chaperones, as well as pathways regulating their activity, constitute potentially significant therapeutic targets in AD. In this respect, the Reader is referred to excellent reviews of the field (references [260,261]).

### 31.2. Conclusions

To conclude, the present perspective presents our current understanding of the molecular basis of Alzheimer’s disease, defines the neuronal ISR-enabled amplification of human AβPP mRNA as the active core of AD, describes various therapeutic strategies for the prevention and treatment of AD, and details principles of design for adequate human neuronal cells-based and transgenic animal models of the disease. Collectively, therapeutic approaches for Alzheimer’s disease and aging-associated cognitive decline outlined in the present study constitute the state of the art in the ACH2.0 paradigm; if successful, they will render both conditions obsolete.

## Data Availability

No new data were created or analyzed in this study. Data sharing is not applicable to this article.
